# ﻿Taxonomy of *Dianthus* (Caryophyllaceae) – overall phylogenetic relationships and assessment of species diversity based on a first comprehensive checklist of the genus

**DOI:** 10.3897/phytokeys.196.77940

**Published:** 2022-05-23

**Authors:** Georgia Fassou, Nadja Korotkova, Anush Nersesyan, Marcus A. Koch, Panayotis Dimopoulos, Thomas Borsch

**Affiliations:** 1 University of Patras, School of Natural Sciences, Department of Biology, Division of Plant Biology, Laboratory of Botany, Patras, Achaia GR-26504, Greece University of Patras Patras Greece; 2 Botanischer Garten und Botanisches Museum Berlin, Freie Universität Berlin, Königin-Luise-Strasse 6–8, 14195 Berlin, Germany Freie Universität Berlin Berlin Germany; 3 A. Takhtajyan Institute of Botany, Armenian National Academy of Sciences, National Academy of Sciences of the Republic of Armenia, Acharyan st. 1, 0040 Yerevan, Armenia A. Takhtajyan Institute of Botany, Armenian National Academy of Sciences Yerevan Armenia; 4 Centre for Organismal Studies (COS) Heidelberg, Department of Biodiversity and Plant Systematics/Botanic Garden and Herbarium Heidelberg (HEID), University of Heidelberg Im Neuenheimer Feld 345, 69120 Heidelberg, Germany University of Heidelberg Heidelberg Germany

**Keywords:** Caryophyllaceae, Caryophyllales, Caucasus, *Dianthus*, EDIT Platform for Cybertaxonomy, Greece, *Petrorhagia*, phylogeny, taxonomy, *Velezia*, World Flora Online

## Abstract

In this study, we present an overall phylogenetic framework for *Dianthus* using four plastid regions (*matK*-*trnK*-*psbA*, *rpl32*-*trnL*, *trnQ*-*rps16*) and nuclear ITS and a species-level checklist for the genus developed by using all available databases and the literature. The trees from the plastid dataset depict a clade of *Dianthus* that also includes *Velezia* and a few taxa of *Petrorhagia*. New combinations in *Dianthus* are provided for these species. The checklist of *Dianthus* in this new delimitation covers 1781 names, with 384 accepted species, 150 subspecies, 12 heterotypic varieties and two forms (not counting autonyms), 1050 synonyms, 22 hybrid names and 172 unresolved names, 3 names were excluded. Implications for the evolution of flower characters, life forms, biogeography, as well as sectional classification are discussed based on the phylogenetic framework.

## ﻿Introduction

The genus *Dianthus* L. (pinks and carnations) is after *Silene* L. the second largest genus in the family Caryophyllaceae. It it was considered to contain approximately 300 species mainly distributed in the northern hemisphere, with many range-restricted or narrow-endemic species in the Mediterranean area, SW Asia, the Caucasus and the Irano-Turanian region, and with some species occurring in eastern and southern Africa (Bittrich 1993). *Dianthus* is a genus with many taxonomically complex species groups, which is perhaps the reason that no comprehensive treatment of the whole genus exists. Valente et al. (2010) reported *Dianthus* to be one of the lineages with the highest species diversification rates in flowering plants, which may explain the evolution of numerous narrow-endemic taxa and render *Dianthus* an interesting model for understanding the evolution of north-temperate floras.

*Dianthus* consists of mostly perennial and a few annual or biennial herbs or shrubs with oblong to ovate or linear and grass-like leaves. According to the widely used circumscription of Pax and Hoffmann (1934), the genus is characterised by hermaphrodite flowers appearing solitary or in more or less dense terminal cymes. The flowers are subtended by 2 to many epicalyx scales and possess a 5-toothed, tubular calyx with 20–60 well-marked parallel nerves. The inclusion of several species formerly classified as *Petrorhagia* (Ser.) Link and *Velezia* L. as suggested by Madhani et al. (2018) adds taxa with fewer (5–15) nerves [*D.candicus* (P.W.Ball & Heywood) Madhani & Heubl., *D.nudiflorus* Griff., *D.recticaulis* Ledeb., and *D.tunicoides* (Ser. ex DC.) Madhani & Heubl). Similarly, the calyx in three of these taxa possesses scarious commissures (*D.candicus*, *D.recticaulis*, *D.tunicoides*) whereas these are missing in *Dianthus* s. str., e.g. as circumscribed by Bittrich (1993), and in *Velezia*. The corolla consists of five distinct petals, with distinct blade and claw. Contrary to the long and pale claw, the limb is brightly coloured (red/pink/purple, yellow) in most species, rarely white. The limb is entire, dentate to laciniate, fimbriate, and without coronal scales. Peltate seeds with a facial hilum and a straight embryo are a characteristic feature of *Dianthus* including *Veleziarigida* L. and apparently all species of *Petrorhagia* (Madhani et al. 2018).

The first infrageneric classification of *Dianthus* was published by Boissier (1867), who recognised six sections, or “sections naturales”; the names he used are considered unranked (Rabeler 1993): *D.* § 5. *Carthusiani* Boiss., *D.* § 4. *Dentati* Boiss., *D.* § 3. *Fimbriati* Boiss., *D.* § 2. *Leiopetali* Boiss. and *D.* § 1. *Verruculosi* Boiss., but this classification was based only on species occurring in the area of his Flora Orientalis. Williams (1893) developed a more comprehensive infrageneric classification system, but apparently did not consider Boissier’s work. Williams described the morphology of the genus in detail and recognised three subgenera with eight sections and 22 subsections in total. The most widely used classification until today is the one of Pax and Hoffmann (1934), who recognised seven sections. An overview of the taxonomic history of *Dianthus* is given in Table 1.

**Table 1.** Summary of the infrageneric classification in *Dianthus* as presented by the respective treatments. The names are given as they were used by the authors, not considering some existing earlier names not used by them.

Boissier 1867	Williams 1885	Pax and Hoffmann 1934
	D.subg.CarthusianastrumF.N.Williamssect.Armerium F.N.Williams	D.subg.Armeriastrum (Ser.) Pax & K.Hoffm. sect. Armerium F.N.Williams
*D.* § 4. *Dentati* Boiss.	D.subg.CarthusianastrumF.N.Williamssect.Suffruticosi F.N.Williams	D.subg.Armeriastrum (Ser.) Pax & K.Hoffm. sect. Suffruticosi F.N.Williams
*D.* § 5. *Carthusiani* Boiss.	D.subg.CarthusianastrumF.N.Williamssect.Carthusiani (Boiss.) F.N.Williams [as “*Carthusianum*”]	D.subg.Armeriastrum (Ser.) Pax & K.Hoffm. sect. Carthusiani (Boiss.) F.N.Williams
	D.subg.CaryophyllastrumF.N.Williamssect.Barbulatum F.N.Williams	D.subg.Caryophyllum (Ser.) Pax & K.Hoffm. sect. Barbulatum F.N.Williams (D.sect.Chamaegarophalon Griseb.)
	D.subg.Caryophyllastrum F.N.Williams sect. Caryophyllum Ser. in Candolle	D.subg.Caryophyllum (Ser.) Pax & K.Hoffm. sect. Eucaryophyllum Graebn.
	D.subg.CaryophyllastrumF.N.Williamssect.Imparjugum F.N.Williams	
*D.* § 3. *Fimbriati* Boiss.	D.subg.CaryophyllastrumF.N.Williamssect.Fimbriati Boiss. [“*Fimbriatum*”]	D.subg.Caryophyllum (Ser.) Pax & K.Hoffm. sect. “*Plumaria*” Opiz
*D.* § 1. *Verruculosi* Boiss.	D.subg.Caryophyllastrum F.N.Williams sect. “*TetralepidesLeiopetala*” F.N.Williams	D.subg.Caryophyllum (Ser.) Pax & K.Hoffm. sect. “*TetralepidesLeiopetala*” F.N.Williams
*D.* § 2. *Leiopetali* Boiss.	D.subg.Proliferastrum F.N.Williams	in *Tunica* Ludw.

The first overall molecular phylogenetic analysis of the Caryophyllaceae (Fior et al. 2006) included *D.furcatus* Balb. and *Dianthusseguieri* Vill. and showed *Veleziarigida* L. as sister to a *Dianthus* clade. Harbaugh et al. (2010) added *D.armeria* L. and an unidentified species of *Dianthus*, but found *Velezia* within the *Dianthus* clade that in turn was sister to *Petrorhagiasaxifraga* (L.) Link. After including more species in their plastid and ITS datasets, Valente et al. (2010) concluded that *Dianthus* is monophyletic if *Velezia* is also included. However, this conclusion was premature with respect to *Petrorhagia* since Valente et al. represented this genus of > 30 species with only two species (*P.thessala* (Boiss.) P.W.Ball & Heywood and *P.prolifera* (L.) P.W.Ball & Heywood) as outgroup, not even including *P.saxifraga* (L.) Link, the type of *Petrorhagia*. Greenberg and Donoghue (2011) then largely confirmed these findings using five chloroplast regions (*matK*, *ndhF*, *trnL*-*trnF*, *trnQ*-*rps16*, *trnS*-*trnfM*) and nrITS, although their tree lacked crucial lineages within *Dianthus*.

In a recent synopsis of the genera of the tribe Caryophylleae, Madhani et al. (2018) formally extended the circumscription of *Dianthus* by three species of *Petrorhagia* and *Veleziarigida*, based on trees of plastid *rps16* and nrITS sequences and mapping of selected morphological characters. They recovered *P.armerioides* (Ser. ex DC.) P.W.Ball & Heywood as sister to *V.rigida* based on ITS but the support for a position of this lineage within *Dianthus* was weak (0.77 PP), and only six species of *Dianthus* were sampled, again lacking crucial lineages. Their *rps16* tree shows *Petrorhagiaalpina* (Hablitz) P.W.Ball & Heywood, *P.armerioides*, and *P.candica* P.W.Ball & Heywood in a subclade that is nested in a *Dianthus* clade. However, only *Dianthuscarthusianorum* L. (only supported by BI, 0.98 PP) and *D.armeria* were included and resolved as successive sisters, whereas *Velezia* was not sampled at all. Moreover, the fact that the authors used different taxa in their plastid and nuclear datasets, some of them crucial for determining generic concepts but represented in only one of the datasets, further limits firm conclusions. The wider circumscription of *Dianthus* proposed by Madhani et al. (2018) is not supported by a morphological synapomorphy because the peltate seeds and straight embryos mentioned therein as important characters for diagnosing *Dianthus* are also found in *Petrorhagia*. The circumscription of *Dianthus* in particular with respect to *Petrorhagia* therefore remains to be clarified by analyses with an improved taxon sampling of both genera and also an increased character base that allows for better resolved trees and statistical support of relevant nodes. These investigations should also consider the Greek and Levant taxa of *Bolanthus* (Ser.) Rchb.. The genus was found closely related to *Dianthus* and *Petrorhagia* based on ITS and *rps16* (Zografidis et al. 2020), which includes *Bolanthushirsutus* (Labill.) Barkoudah, the type of *Bolanthus*.

Valente et al. (2010) conducted the first comprehensive molecular analysis of *Dianthus*, based on partial *matK*, *psbA-trnK*, *trnH-psbA* and nrITS sequences from 104 species plus some representatives from *Velezia*, *Petrorhagia* and *Saponaria* L. Based on their trees, the authors annotated five major lineages within *Dianthus*. The first branching lineage largely comprised members of D.sect.Armerium F.N.Williams (e.g. *D.armeria*, *D.deltoides* L., *D.viscidus* Bory & Chaub.), followed by a *Velezia* clade (represented by three different samples of *V.rigida*), and a lineage called “Section Verruculosi” with *D.cyri* Fisch & C.A.Mey., *D.strictus* Banks ex Sol. and *D.tripunctatus* Sm. The majority of the species then appeared in a clade annotated as “Eurasian radiation” that was depicted as sister to an African clade. However, the Eurasian clade had almost no internal resolution.

*Dianthus* has received considerable interest from botanists in recent years, focusing on individual species groups and the description of new taxa or treatments in specific geographic areas (Balao et al. 2009; Kuzmina and Nersesyan 2012; Deniz et al. 2016; Ferrer Gallego and Laguna 2018; Oskay 2018; López-Jurado et al. 2019) and conservation (Kołodziejek et al. 2018; Cogoni et al. 2019). As a result, 54 species and 18 subspecies, mostly from the Euro-Mediterranean area, were described as new to science the last 15 years.

The absence of an overall phylogenetic tree of *Dianthus* as a prerequisite to break up the genus into workable units, in which species level relationships then can be studied in more detail with evolutionary methods, has so far limited the analysis of species limits in *Dianthus*. Also lacking is a comprehensive treatment or checklist, which would be fundamental to inform sampling and provide a basis for synthesising the existing information on *Dianthus*. Considering the wealth of new descriptions, we assumed that the diversity of *Dianthus* may in fact be considerably higher than 300 species as cited by Pax and Hoffmann (1934) and Bittrich (1993). *Dianthus* represents one of the major genera of the order Caryophyllales (Hernández-Ledesma et al. 2015), for which a globally consistent species-level taxonomic backbone is developed by the Caryophyllales Network, which also contributes to the treatments for World Flora Online (WFO, www.worldfloraonline.org) (Borsch et al. 2020).

As a starting point for this study, we took advantage of ongoing research activities in the context of the Caucasus Plant Biodiversity Initiative and the Flora of Greece project (http://portal.cybertaxonomy.org/flora-greece/intro), all covering geographic areas with high species diversity of the genus, and in which *Dianthus* is currently studied. In Greece, *Dianthus* is represented by 44 species and 43 subspecies, of which about half are endemic. Some of them are morphologically and geographically divergent, such as the *D.juniperinus* Sm. and *D.fruticosus* L. groups, which are woody chasmophytes occurring mostly on the island of Crete (Dimopoulos et al. 2013). In the Caucasus, about sixty species are native, of which 20 are endemic (Kuzmina and Nersesyan 2012), and five further endemics occur in NE Turkey and SW Iran.

The specific objectives for this paper are therefore twofold: first, to generate an overall phylogenetic hypothesis for *Dianthus* that covers a broad spectrum of taxa, also extending to putative close relatives (e.g. *Petrorhagia*) to further test the monophyly of *Dianthus*. Due to the extremely low genetic diversity encountered in *Dianthus* as compared to other large genera of flowering plants (e.g. *Campanula*), we sequenced four plastid regions (*matK*-*trnK*-*psbA*, *rpl32*-*trnL*, *trnQ*-*rps16*) that were selected for high variability and hierarchical phylogenetic signal (Borsch and Quandt 2009) as well as to match existing datasets of Valente et al. (2010). We added nrITS to obtain data from a nuclear region that allows for some first insights to possible hybridisation and reticulate evolution.

The second specific objective is to provide an up-to-date taxonomic backbone for *Dianthus*. This was done using an import that integrated all electronically available sources from the World Flora Online. In this way, an already comprehensive name source was available that we then matched with recent treatments, e.g. major Floras published in the last thirty years, accounts of specific species groups, etc., to have a clear reference for accepting a name or putting it into synonymy. The checklist of *Dianthus* presented here will be also incorporated into the World Flora Online taxonomic backbone and will be updated whenever changes become necessary.

Considering the difficulty in species delimitation and the species diversity of *Dianthus*, we believe that our integrated approach to develop both a taxonomic backbone that includes all names belonging to the genus in a current, monophyletic circumscription, along with a traceable source for the taxon concepts used at species level, will be crucial to understanding evolutionary relationships and species diversification in space and time. Such information is also urgently needed from applied perspectives such as to assess the conservation status of many *Dianthus* species that are local or regional endemics and/or have a critical conservation status.

## ﻿Methods

### ﻿Taxon sampling and collection of plant material

Plant material was collected in the field or from well-documented accessions in the living collections of the Botanic Garden Berlin, and from herbarium specimens kept at the herbaria B and ERE. Information on the origin of all samples is provided in Appendix 1. Vouchers are deposited in B, ERE, HEID and UPA.

### ﻿DNA extraction, amplification and sequencing

Total genomic DNA was extracted from silica-dried leaf material using the NucleoSpin Plant II kit (Macherey Nagel, Düren, Germany) and from herbarium samples using a CTAB protocol with triple extractions (Borsch et al. 2003). DNA fragments were amplified through polymerase chain reaction (PCR) in 50 µl volumes, containing 4 μl of DNA template (concentration c. 10 ng/μl), 5 μl Taq buffer S (PeqLab, Erlangen, Germany), 2 μl of each primer (20 pm/μl), 10 μl dNTPs (each 1.25 mM), 1.5 units of Hot Taq DNA Polymerase (PeqLab), and ultrapure water.

The *matK-trnK-psbA* region was amplified in overlapping halves using the primer pair trnK-F (Wicke and Quandt 2009) and CARYmatK-1440R (Schäferhoff et al. 2009) for the 3′ fragment and ACmatK500F (Müller and Borsch 2005), and psbA5’R (Shaw et al. 2005) for the 5′ fragment. The use of the reverse primer psbA5′R that anneals to the psbA gene allows the full sequence at the 5′ end of the *trnK* intron to be obtained and additionally covers the *trnK-psbA* intergenic spacer. Amplification conditions were: an initial denaturation step of 1 min 30 sec at 96 °C, followed by 34 cycles of denaturation (30 sec at 95 °C), annealing (1 min at 50 °C), extension (1 min 30 sec at 72 °C), and a final extension step (20 min at 72 °C).

The *rpl32-trnL* IGS was amplified using the primers rpl32-F and trnL-UAG (Shaw et al. 2007) and the *trnQ-rps16* IGS was amplified using the primers trnQ2 (Korotkova et al. 2010) and rpl16x1 (Shaw et al. 2007). The PCR conditions were as for *matK-trnK-psbA*, except that the extension step was only 1 minute. ITS was amplified using the primers ITS5 and ITS4 (White et al. 1990), amplification conditions were: 35 cycles of denaturation (1 min at 97 °C), annealing (1 min at 48 °C) and extension (45 s at 72 °C), and a final extension step (7 min at 72 °C).

All PCR products were electrophoresed for 2 hours on a 2% agarose gel and then excised and purified using the Geneaid Gel/PCR DNA Fragments Extraction Kit (Geneaid Biotech Ltd., New Taipei City, Taiwan) and sequenced via standard Sanger sequencing at Macrogen Europe (Amsterdam, The Netherlands). Chromatograms were inspected by eye, erroneous nucleotide calls were manually corrected, and final sequences were assembled using PhyDE v. 0.9971 (Müller et al. 2005+). All sequences were submitted to the European Nucleotide Archive (ENA) https://www.ebi.ac.uk/ena/browser/home under the projects PRJEB48120, PRJEB43752.

### ﻿Sequence alignment, indel coding and model selection

DNA sequences were aligned in PhyDE following a motif alignment approach and the rules laid out by Löhne and Borsch (2005). Homonucleotide stretches and parts of uncertain homology were excluded from the final matrix prior to the analyses. Indels were coded according to the scheme “Simple Indel Coding” (Simmons and Ochoterena 2000) in SeqState v.1.40 (Müller 2005b).

### ﻿Phylogenetic analyses

Best-fitting models of nucleotide substitution were selected via the Akaike Information Criterion in jModeltest v.2.1.6 (Darriba et al. 2012). The models that were found to best fit the given DNA sequence data are listed in Table 2. Phylogenetic reconstructions were performed via Maximum Likelihood (ML) and Bayesian Inference (BI). Jackknife (JK) node support was additionally calculated under parsimony in PAUP* v.4.0b10 (Swofford 1998) with 10,000 replicates, branch swapping via tree-bisection-reconnection, a deletion of 36.8% of all characters during replicates, and a retention of one tree per replicate (Müller 2005a).

**Table 2.** Sequence characteristics of the individual partitions in the plastid dataset. SD = standard deviation.

	*rpl32-trnL*	*trnQ-rps16*	*trnK intron*	*matK* CDS	*trnK-psbA*	ITS
**Dataset including hotspots**						
Number of sequences	202	202	202	202	202	145
Aligned length	1372	1166	1229	1536	270	714
Mean length (SD)	786 (89)	688 (120)	815 (163)	1406 (332)	195 (95)	674 (33)
**Dataset excluding hotspots**						
Aligned length	1236	1068	1186	1537	270	714
Mean length (SD)	755 (78)	653 (114)	793 (160)	1405 (332)	195 (95)	674 (33)
% variable characters	31.5	23.6	16.8	22.4	16.2	33.3
% informative characters	18.8	8.3	5.3	8.8	4.4	27.5
Number of coded indels	106	102	52	17	10	43
jModeltest v.2.1.6, AIC results	GTR+G	TPM1uf+G	TPM1uf+I+G	TPM1uf+I+G	--	SYM+I+G

Tree inference under ML was conducted on the concatenated alignment with RAxML v.8.2.9 (Stamatakis 2014) using the thorough ML optimisation option. The dataset was analysed as a single partition under the nucleotide substitution model GTR+I+G, with branch lengths linked across partitions. Branch support for the ML inference was calculated via 100 bootstrap (BS) replicates using the rapid BS algorithm (Stamatakis et al. 2008).

Bayesian Inference (BI) was conducted with MrBayes v.3.2.2 (Ronquist and Huelsenbeck 2003), using four parallel Markov Chain Monte Carlo (MCMC) runs for a total of 10 million generations on CIPRES. The initial 25% of all MCMC trees were discarded as burn-in, and post-burn-in trees were summarised as majority rule consensus trees. Datasets for BI comprised both DNA sequence data and coded indels; the binary character model was applied for the indel partition. All trees were visualised via TreeGraph2 (Stöver and Müller 2010).

### ﻿Compilation of the *Dianthus* checklist

The checklist was compiled using the EDIT Platform for Cybertaxonomy (cybertaxonomy.org) (Ciardelli et al. 2009; Berendsohn 2010), which is a suite of open-source software tools and services that covers all aspects of an integrative taxonomic workflow and include tools to capture, process, attribute, document, publish and maintain the data. This way, the already existing interaction with the Global Caryophyllales Synthesis initiative (Borsch et al. 2015) can be used and future dynamic updating is guaranteed.

As a first step, a complete list of *Dianthus* names and their respective World Flora Online (WFO)-IDs were received from the WFO Data Centre in February 2018 and imported into the EDIT Platform. This import included names accepted in the WFO backbone, their synonyms therein, doubtful names, and names of hybrids, and each of these taxonomic states was preliminarily assigned to the imported names. This dataset was then matched with the World Checklist of Vascular Plants dataset received from the Royal Botanic Gardens, Kew in December 2019 (Kew WCVP 2019), which resulted in some 181 additional, mostly infraspecific names, not yet covered by the WFO backbone, which were manually entered into the EDIT platform as well.

This preliminary list of accepted names and their synonyms was then cross-checked with relevant taxonomic treatments (see below). The resulting circumscription of each taxon is indicated by means of a “secundum” (“sec.”) reference (Berendsohn 1995; 1997; Berendsohn and Geoffroy 2007), which is the particular reference for the taxon to be accepted in our taxonomic backbone. For synonyms, the “syn. sec.” reference indicates the assignment of the synonym to the concept of either the accepted name or one of its homotypic synonyms; this may or may not be the same reference as that of the taxon’s secundum. We selected the secundum reference to be the most comprehensive source of information available for a species or subspecies. Ideally, this was a monographic treatment based on morphology and a detailed revision of type material, as carried out for some Floras. In those cases where a newly described species had not yet been included in any subsequent more inclusive treatment, the original publication served as the secundum reference. The references used as secundum for the Euro-Mediterranean area in the widest sense included the treatments of *Dianthus* in the Euro+Med PlantBase (Marhold 2011), Flora Iberica (Bernal et al. 1990), Vascular Plants of Greece: an annotated checklist (Dimopoulos et al. 2013) and Flora Iranica (Rechinger 1988). Several checklists and Floras were examined for Russia and the Caucasus (Czerepanov 1995; Barkalov and Probatova 2006; Chepinoga et al. 2008; Kuzmina and Nersesyan 2012). For eastern Asia, the sources were the Floras of China (Dequan and Turland 2001), Japan (Zoku 1965) and Pakistan (Ghanzafar and Nasir 1986). The African Plant Database version 3.4.0 (2012) was taken as the primary reference for the African species. These references already covered about 90% of the *Dianthus* species. Further publications reporting taxonomic data were used, such as descriptions of new species (Brullo et al. 2000; Shaulo and Erst 2011; Brullo et al. 2015; Son et al. 2017), nomenclatural notes on certain species or species groups (Peruzzi and Gargano 2006; Bacchetta et al. 2010) and studies on species in single countries (Iamonico 2013; Lazkov 2016). Many new *Dianthus* species were described from Turkey in the last 10–15 years, which were considered through the respective publications (Menemen and Hamzaoğlu 2000; İlçim et al. 2013; Hamzaoğlu et al. 2015; 2017; 2018; Deniz et al. 2016).

In the rare cases of differing taxonomic concepts, we accepted the circumscription with the respective secundum reference that was either the most recent one or the one covering the area where the accepted taxon in question is primarily distributed.

Names that were part of the WFO backbone, treated therein as unresolved names and not found in any of the sources cited above, were also treated as unresolved names in our checklist.

## ﻿Results

### ﻿Sequence datasets

The concatenated plastid alignment matrix comprised a total of 202 taxa and 5573 positions, which resulted in a matrix of 5297 nucleotide characters and 287 coded indels. The ITS matrix contained only 136 taxa and was 714 nucleotides in length, with an average length of 674 nucleotides. There were polymorphic sites in about one-third of the generated ITS sequences, including some obvious hybrid sequences which were not readable. For this reason, the ITS matrix only includes unambiguously readable sequences and is therefore smaller than the plastid matrix. Detailed sequence statistics are provided in Table 2.

### ﻿Trees inferred from the concatenated plastid dataset

Our annotation in the phylogenetic trees uses accepted names as available prior to this study; new names or combinations in order to make *Dianthus* monophyletic are provided below.

**Figure 1.**
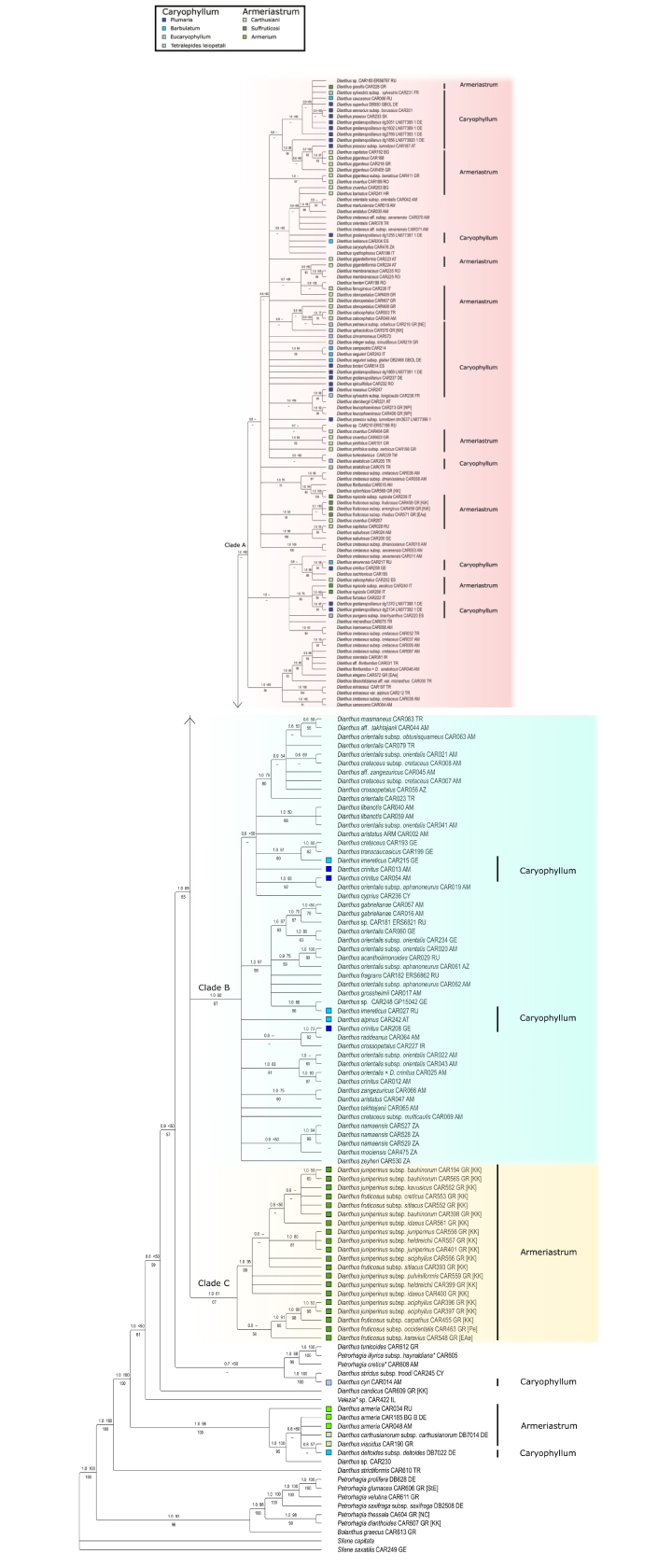
Bayesian majority-rule consensus tree inferred from combined plastid dataset of *trnK*/*matK*, *trnK*-*psbA*, *trnQ*-*rps16*, *rpl32*-*trnL*. Support values PP (left) and MLBS (right) can be found above branches and JK below; “<50” denotes branches with MLBS support below 50%, “--” denotes a node not found by the respective analysis. Country codes: AM = Armenia, AT = Austria, AZ = Azerbaijan, BG = Bulgaria, CY = Cyprus, DE = Germany, ES = Spain, FR = France, GE = Georgia, GR = Greece (NE = North East, NC = North Central, KK = Kriti and Karpathos, StE = Sterea Ellas, NPi = Norh Pindos, EAe = East Aegean Islands, Pe = Peloponnisos), HR = Croatia, IR = Iran, IT = Italy, RO = Romania, RU = Russia, SK = Slovakia, TR = Turkey, ZA = South Africa. The annotations on the infrageneric classification based on Pax and Hoffmann (1934) are indicated by coloured squares.

The plastid tree (Fig. 1) depicts a maximally supported clade comprising all sampled *Dianthus* taxa as well as *Velezia* and several *Petrorhagia*, which are nested within *Dianthus*. *Petrorhagiaalpina* (= *Dianthusrecticaulis* Ledeb.) is resolved as sister to the rest of the *Dianthus*-*Velezia*-*Petrorhagia* clade. The other *Petrorhagia* samples are resolved in a separate highly supported clade including *P.saxifraga*, the type species of the genus, with *Bolanthusgraecus* (Schreb.) Barkoudah sister to the rest of *Petrorhagia*.

A *Dianthusarmeria* clade (1 PP, 98% MLBS and 100% JK) is the first branching lineage. The next branching lineages are *Velezia*, *Dianthuscandicus*, and a clade comprising *Dianthuscyri*, *D.strictus*, *D.tunicoides* and two further species of *Petrorhagia* (*P.cretica* (L.) P.W.Ball & Heywood and *P.illyrica* (Ard.) P.W.Ball & Heywood). However, the branching order among these clades only receives moderate (BI) to weak support.

The core of *Dianthus* that includes most species (1 PP, 69% MLBS and 65% JK) is composed of three main clades. Clade A (1 PP, < 50% MLBS) contains a wide spectrum of Eurasian taxa. Clade B (1 PP, 90% MLBS and 87% JK) contains mainly Irano-Turanian and Caucasian taxa and all species from southern Africa. Clade C (1 PP, 61% MLBS and 67% JK) consists of *Dianthusjuniperinus* and most subspecies of *D.fruticosus*, but D.fruticosussubsp.amorginus Runemark, subsp. fruticosus , and subsp. rhodius (Rech. f.) Runemark are resolved in a sublineage of clade A.

### ﻿Trees inferred from nrITS

**Figure 2.**
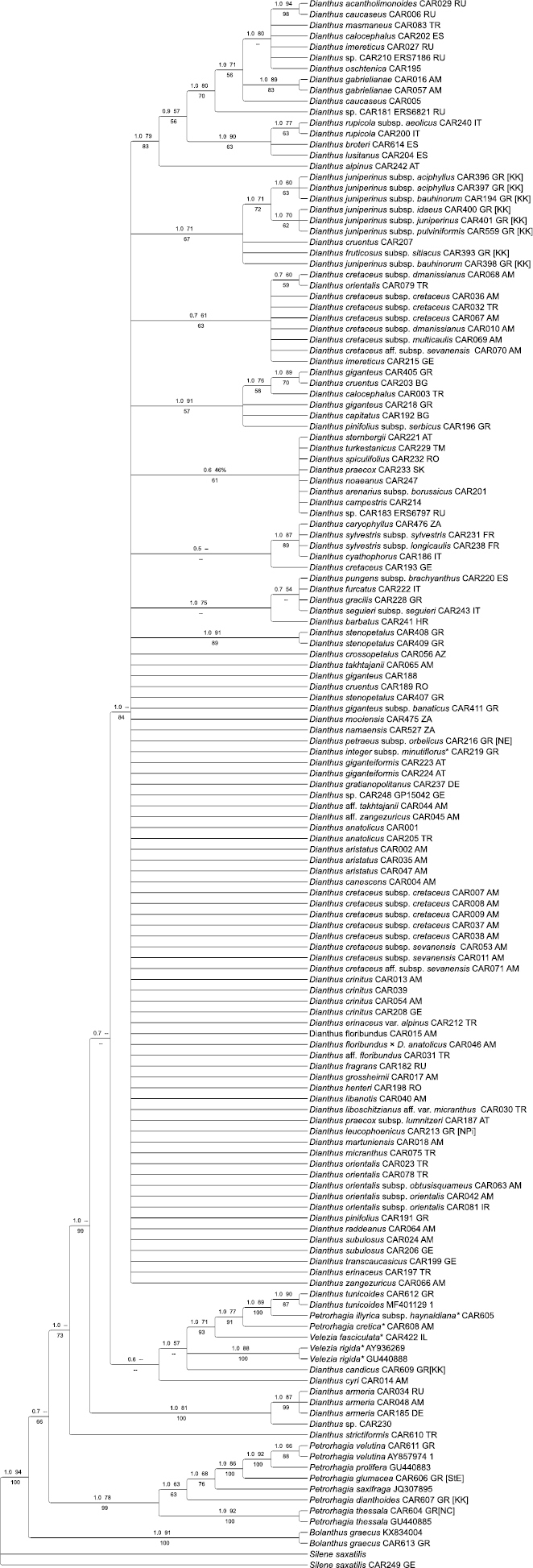
Bayesian majority-rule consensus tree inferred from nuclear ITS datasets. Support values PP (left) and MLBS (right) can be found above branches and JK below; “<50” denotes branches with MLBS support below 50%, “--” denotes a node not found by the respective analysis. Country codes: AM = Armenia, AT = Austria, AZ = Azerbaijan, BG = Bulgaria, CY = Cyprus, DE = Germany, ES = Spain, FR = France, GE = Georgia, GR = Greece (NE = North East, NC = North Central, KK = Kriti and Karpathos, StE = Sterea Ellas, NPi = Norh Pindos, EAe = East Aegean Islands, Pe = Peloponnisos), HR = Croatia, IR = Iran, IT = Italy, RO = Romania, RU = Russia, SK = Slovakia, TR = Turkey, ZA = South Africa. The annotations on the infrageneric classification based on Pax and Hoffmann (1934) are indicated by coloured squares.

The ITS topology (Fig. 2) is hardly resolved at all. Still, the tree depicts a well-resolved core of species of *Petrorhagia* in a clade sister to the *Dianthus* clade. *Dianthusrecticaulis* (= *Petrorhagiaalpina*) is again depicted as first branch, followed by the *D.armeria* lineage, which is congruent to the plastid tree. *Dianthuscyri*, the species of *Velezia*, *Dianthustunicoides* along with *Petrorhagiacretica* and *P.illyrica* are found in a weakly supported clade, inconsistent to their positions in a rather weakly supported grade in the plastid tree. *Bolanthus* (= *Graecobolanthus*) is resolved in the first branch in the tree, albeit with weak support (0.7 BI, 66% JK).

Unlike the plastid sequences, ITS sequence data do not provide resolution within the core of *Dianthus*, although some subclades are evident within a broad polytomy (Fig. 2). The lineage with subspecies of *D.juniperinus* together with D.fruticosussubsp.sitiacus Runemark corresponds to subclade C in the plastid tree, whereas neither the *D.broteri* Boiss. & Reut. - *D.rupicola* Biv. - *D.lusitanus* Brot. subclade of the ITS tree nor the subclade with *D.caucaseus* Sims, *D.gabrielianae* Nersesian and *D.oschtenica* are recovered by plastid data. Also, the southern African species (e.g. *D.mooiensis* F. N. Williams, *D.zeyheri* Sond) appear within the core polytomy in ITS.

### ﻿Accepted species names and synonyms

In total, 1781 names are included for a monophyletic genus *Dianthus* as defined here, including *Petrorhagia* p.p. and *Velezia*. The resulting checklist treatment is subdivided into four parts: i) the core checklist that contains the accepted species and infraspecies and their synonyms, ii) hybrid names, iii) unresolved names and iv) excluded names. The core checklist of *Dianthus* including 2 former *Petrorhagia* species and 3 heterotypic subspecies and 6 former *Velezia* species contains 384 total accepted species and 150 accepted heterotypic subspecies, and 1050 names are assigned as synonyms. There are 22 hybrid names, 172 unresolved names, and 3 excluded names.

## ﻿Discussion

### ﻿Circumscription and overall relationships of *Dianthus*

**Figure 3.**
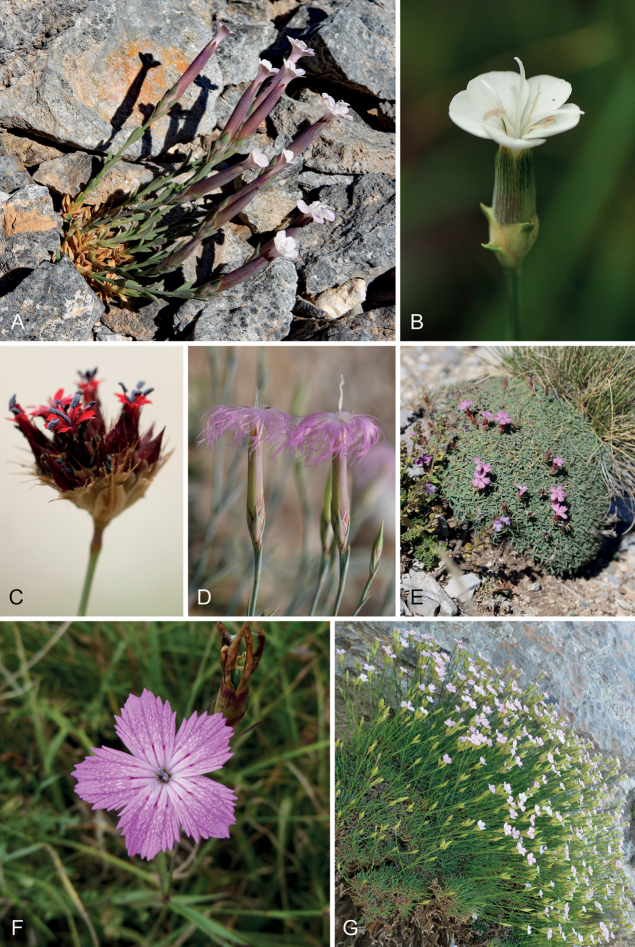
Habit, inflorescence and floral morphology in the core lineage of *Dianthus*. From the predominantly Euro-Mediterranean clade A are *D.sphacioticus* (**A**) with solitary flowers enclosed basally by cauline leaves, D.integersubsp.minutiflorus (**B**) enclosed basally by several slightly coriaceous bracts with an outward-pointing green tip, *D.stenopetalus* (**C**) with several flowers in a condensed terminal inflorescence with many brown scales, *D.critinus* (**D**) with four decussate bracts at the base, and *D.haematocalyx* (**E**). Clade B, which includes many Caucasian and Irano-Turanian species, is represented by *D.gabrielianae* (**F**) and the predominantly Cretan clade C by D.juniperinussubsp.juniperinus (**G**). *D.juniperinus* is a densely branched, cushion-like subshrub and *D.haematocalyx* is a small cushion plant. Photos: N. Turland (**A**), K. Goula (**B–D**), A. Zografidis (**E**), A. Nersesyan (**F**) and G. Fassou (**G**).

**Figure 4.**
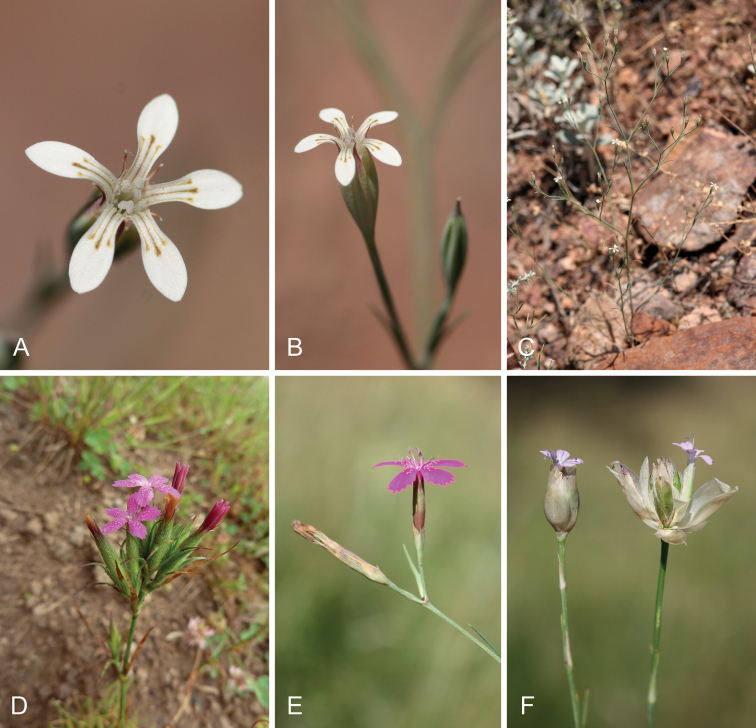
Habit, inflorescence and floral morphology in early-branching lineages of *Dianthus* and *Petrorhagia*. The *Dianthustunicoides* clade is represented by *D.pachygonus* (= *Petrorhagiacretica*), which has solitary flowers (**A, B**) in diffusely branched inflorescences (**C**). The *Dianthusarmeria* clade is represented by *D.armeria* (**D**), which has condensed terminal inflorescences and flowers supported by reflexed, bract-like leaves, and *D.deltoides*, which has lax inflorescences with solitary flowers each supported by two opposite bracts (**E**). The core clade of *Petrorhagia* is represented by *P.prolifera*, which has highly condensed terminal inflorescences with broadly ovate and thinly scarious bracts enclosing the flowers (**F** with opened inflorescence to the right). Photos: A. Nersesyan (**A-D**), T. Borsch (**E, F**).

Plastid and nuclear data agree on a *Dianthus* clade that includes *Velezia* and further species of *Petrorhagia* (*P.cretica*, *P.illyrica*) as deeply nested (Figs 1, 2) that were not sampled in previous phylogenetic analyses (Valente et al. 2010; Madhani et al. 2018). It is noteworthy that *Dianthusrecticaulis* is resolved as sister to all other species of *Dianthus* in both genomic compartments with good support. It is an annual with solitary flowers on conspicuous peduncles (Ball and Heywood 1964) similar to *P.cretica* (Fig. 4A, B). The inflorescence architecture is apparently connected to the annual life form, which evolved independently in different terminal branches in *Dianthus* and allies. *Dianthusrecticaulis* is the legitimate name for *P.alpina*, while *Dianthusstrictiformis* that was proposed as a new name for *Petrorhagiaalpina* by Madhani et al. (2018) is superfluous and illegitimate (Mosyakin and Federonchuk 2018). Their plastid *rpl16* tree, however, depicts this species in an incongruent position as sister to *P.candica* (also transferred to *Dianthus* by the authors), but this may be a spurious signal caused by an imbalanced sampling of *Dianthus* (both species appear nested within the *D.armeria* clade). Our phylogenetic results confirm the position of these former *Petrorhagia* species within *Dianthus* (Figs 2, 3). Nevertheless, we extended the sampling of *Petrorhagia* and found further species (*P.cretica*, *P.illyrica*) within *Dianthus*, whereas a distant clade of the members of the genus *Petrorhagia* (including the type *P.saxifraga*, and *P.dianthoides* (Sm.) P.W.Ball & Heywood, *P.glumacea* (Bory & Chaub.) P.W.Ball & Heywood, *P.prolifera*, *P.thessala* and *P.dubia* (Raf.) G.López & Romo) receives high support in chloroplast and ITS trees. The taxa that were found nested within *Dianthus* belong to the Petrorhagiasect.Pseudotunica (Fenzl) Ball & Heywood and Petrorhagiasect.Pseudogypsophila (A.Braun) P.W.Ball & Heywood, while the *Petrorhagia* taxa that form a distant clade belong to Petrorhagiasect.Petrorhagia and Petrorhagiasect.Kohlrauschia (Kunth) P.W.Ball & Heywood (Ball and Heywood 1964). This clade is related to *Bolanthus*, which is inferred as sister with high support in the plastid tree, but is depicted inconsistently in a grade with ITS (Fig. 2). Apart from our now clearer picture of relationships between *Dianthus* and *Petrorhagia*, the genus *Velezia* still plays a role in the monophyletic circumscription of *Dianthus*. Madhani et al. (2018) merged *Velezia* with *Dianthus*, following the suggestion by Valente et al. (2010), but considered only one species, *V.rigida* L., and resurrected the name *Dianthusnudiflorus* Griff. based on a type from Afghanistan. Sequence data of *Velezia* indicate that this former genus consists of more than one species (Fig. 2), with *V.fasciculata* Boiss. in a different position in the ITS tree compared to *V.rigida*, which is in line with the acceptance of six species in the Euro+Med PlantBase (Marhold 2011). Only *V.rigida* is widespread from the western Mediterranean through SW Asia and the Caucasus as well as introduced in North America, specifically in California (Rabeler and Hartmann 1993+), whereas the other species are range-restricted and occur in Greece, Turkey and Syria. Consequently, we propose to also include the remaining *Velezia* species in *Dianthus* (see Nomenclatural Novelties).

The second branch within the *Dianthus* clade is composed of *Dianthusarmeria* (incl. *D.viscidus*) and relatives such as *D.carthusianorum* and *D.deltoides*, as congruently inferred by the nuclear and plastid partition and which is also consistent with trees shown by Valente et al. (2010) and Greenberg and Donoghue (2011). The respective positions of the next-branching lineages, i.e. *Velezia* and a lineage of *Dianthusstrictus* plus *D.cyri*, are not well supported. Again, our tree topologies agree in principle with the findings of Valente et al. (2010) and Greenberg and Donoghue (2011) but differ in that two former species of *Petrorhagia* as recognised by Madhani et al. (2018) belong here (*P.armerioides* (Ser. ex DC.) P.W.Ball & Heywood, *P.candica* P.W.Ball & Heywood). We show that *P.cretica* and *P.illyrica* are also part of this lineage (Fig. 2) and need to be merged with *Dianthus*.

Our results also agree with a core clade of *Dianthus* that contains the majority of the *Dianthus* species. Contrary to Valente et al. (2010), our results depict three major clades (clades A-C) of core *Dianthus*.

Clade A contains Eurasian taxa and includes the type of the genus, *D.caryophyllus*. Clade A also comprises three subspecies of *D.fruticosus* (subsp. amorginus, subsp. fruticosus and subsp. rhodius) that are apparently convergent in terms of evolving a similar life form as the members of clade C. Several terminal sublineages of apparently geographically close samples receive good support such as D.cretaceusAdamssubsp.cretaceus and *D.floribundus* Boiss., but at the same time the samples of *D.gratianopolitanus* Vill. are spread over this clade (Fig. 2).

Clade B contains mostly Irano-Turanian and Caucasian taxa but also *D.cyprius* A.K.Jacks. & Turrill﻿ from Cyprus, *D.alpinus* L. from the European Alps and the taxa from tropical and southern Africa (*D.mooiensis*, *D.namaensis* Schinz﻿ and *D.zeyheri*). This is in contrast with the claim of Valente et al. (2010) that an African clade is well supported as a sister clade to a Eurasian radiation. According to our data, the African taxa are nested within the Eurasian radiation.

The third clade of the core of *Dianthus* (clade C) consists of the Cretan taxa *Dianthusjuniperinus* and some subspecies of *Dianthusfruticosus*. Specifically, the subspecies D.fruticosussubsp.carpathus Runemark, D.fruticosussubsp.occidentalis Runemark and D.fruticosussubsp.karavius Runemark can be found in clade C together with *D.juniperinus*. *Dianthusfruticosus* is not monophyletic; some of its subspecies, D.fruticosussubsp.fruticosus, D.fruticosussubsp.amorginus and D.fruticosussubsp.rhodius are resolved within clade A. There are no apparent morphological differences of D.fruticosussubsp.amorginus and subsp. fruticosus (both from the Cyclades), and D.fruticosussubsp.rhodius (from Rhodes and other east Aegean islands) compared to the other subspecies of *D.fruticosus* from clade C. This indicates convergent evolution resulting from adaptation to similar coastal rocky habitats.

### ﻿Infrageneric classification of *Dianthus*

The classification of Pax and Hoffmann (1934) is annotated on the phylogenetic trees in Fig. 1. Even though many species were described later and therefore were not classified into infrageneric entities, it is evident that these subgenera and sections do not represent natural groups. Apart from D.sect.Armerium, there is little correlation with the taxonomic groups of either Williams (1893) or Pax and Hoffmann (1934).

Dianthussubg.Armeriastrum is highly polyphyletic and is represented both in the core of *Dianthus*, namely in clades A and C by D.sect.Carthusiani and D.sect.Suffruticosi, but also in the *Dianthusarmeria* clade (with D.sect.Armerium) that belongs to the first branches of *Dianthus*. However, D.sect.Armerium is paraphyletic to *D.deltoides* (D.subg.Caryophyllum). Clade C has exclusively taxa from D.sect.Suffruticosi, but the section itself is not limited to this clade C. It is polyphyletic due to the presence of *D.armeria* outside of the core *Dianthus* clade, the presence of three subspecies of *D.fruticosus*, and two further independent terminal lineages composed by *D.rupicula* and *D.gracilis* in clade A. The suffruticose life form that characterises D.sect.Suffruticosi must therefore have evolved multiple times.

Dianthussubg.Caryophyllum is in the clades A and B, with its sections forming no specific pattern. Although all the sections appear in the core of *Dianthus*, D.sect.Eucaryophyllum appears exclusively in clade A, while the section “*Tetralepidesleiopetala*” can be found in clade A and outside of the core. What was referred to as a clade corresponding to D.sect.Armerium in Valente et al. (2010) in fact consists of taxa from different sections sensu Pax and Hoffmann (1934). In addition to D.sect.Armerium, these are D.sect.Carthusiani (*D.carthusianorum* and *D.viscidus*), although the majority of species from this section are in clade A. *Dianthusdeltoides*, which is congruently inferred as part of the *Dianthusarmeria* clade by Valente et al. (2010), is classified herein in D.subg.Caryophyllum.

The early branch of *Dianthuscyri* and *D.strictus* belongs to the section “*Tetralepidesleiopetala*” according to Pax and Hoffmann (1934), which was originally described by Williams (1885); see Table 1. The two species *D.cyri* and *D.tripunctatus* (not sampled here) were earlier used by Boissier (1867) to define *D.* § 1. *Verruculosi*, although no type for the sectional name was designated. If we accept *D.cyri* as the type, the name D.sect.Verruculosi (Boiss.) Schischkin would be correct in terms of priority. This corresponds to the use of this sectional name by Valente et al. (2010), as well as some floristic treatments, e.g. Rechinger (1988). However, Williams (1885; 1893) did not designate any types for his subgeneric or sectional names either. The wide taxon concept of the section “*Tetralepidesleiopetala*” sensu Williams (1893) and Pax and Hoffmann (1934) can only be adjusted after the application of a validly published name and its typification. This section is polyphyletic and its species are found across all our clades (Fig. 1).

### ﻿Implications for the evolution of floral and inflorescence morphology

The inflorescences in *Dianthus*, *Petrorhagia* and related genera possess a cymose principal structure as in most Caryophyllaceae, and these inflorescences exhibit various levels of complexity. The spectrum ranges from solitary flowers on more or less unbranched stems (e.g. *D.sphacioticus* Boiss. & Heldr.﻿, Fig. 3A), over more or less richly branched inflorescences (e.g. *D.deltoides*, *D.juniperinus*, Figs 3G, 4C, 4E) to somewhat condensed (e.g. *D.armeria*, Fig. 4D) or strongly condensed terminal head-like inflorescences (e.g. *D.stenopetalus* Griseb., Fig. 3C). These complex inflorescences have evolved independently in different lineages within *Dianthus*, such as the early-branching *Dianthusarmeria* clade (*D.armeria*, *D.carthusianorum*) and within clade A of the core group (*D.cruentus* Griseb., *D.giganteus* d’Urv., *D.pinifolius* Sm., *D.stenopetalus*) and also within clade B (*D.asperulus* Boiss. & A.Huet, *D.transcaucasicus* Schischk.). In a similar way, the condensed inflorescences in *Petrorhagiaprolifera* and relatives (Fig. 1C, Fig. 4F; Ball and Heywood 1964) mark convergent evolution in a terminal clade, whereas *P.saxifraga* has solitary flowers subtended by four decussate bracts, like in several species of *Dianthus* in core clades A-C (Fig. 3). Within the inflorescences, cauline herbaceous leaves more or less gradually become narrower, more scarious or papery in texture and with a more distinctly excurrent midrib (Figs 3D, 4E) toward the tip of the branches. The position of the uppermost pair of these leaf organs can be distinctly below the calyx as in *Petrorhagiacretica*, and in this case they differ only slightly in their morphology from cauline leaves. Alternatively, these leaf organs arise directly at the base of the calyx, resulting from a reduced peduncle and shortened uppermost internodes of the inflorescence branches or stems (Figs 3 B, D, 4E). This seems to be the most common state in *Dianthus*, which is present in all major lineages. These “subtending” modified leaf organs usually differ more abruptly from the upper leaves on stems and inflorescence branches and have been called “epicalyx scales” or “epicalyx bracts” (Ball and Heywood 1964; Tutin 1964; Madhani et al. 2018;) . Condensed inflorescences with multiple flowers do not only have such “epicalyx scales” but also additional, usually brown, scarious bracts (Fig. 3C) that create a firm, head-like appearance.

This was noted by Tutin (1964), who stated that bracts occur in species with capitate inflorescences and should be distinguished from epicalyx scales. Specialised inflorescence types have also evolved in other genera of Caryophyllales, such as the head-like synflorescences composed of several paracladia that are supported by pseudanthial leaves in *Gomphrena*, Amaranthaceae (Ortuño Limarino and Borsch 2020). Therefore, complex evolution of inflorescences is present in the Caryophyllales, and occurs multiple times within the respective genera. The absence or presence of “epicalyx bracts” as discussed by Ball and Heywood (1964) and Madhani et al. (2018) can therefore not be used as diagnostic to delimit *Dianthus* from *Petrorhagia* and as such does not represent a homologous character. Nevertheless, the evolution of complex inflorescence types, which possess additional modified leaf organs compared to the subtending opposite leaves in dichasial inflorescence structures, may be more accurately reconstructed once there is a fully resolved tree of *Dianthus*.

The conspicuous species with plumose petals (Fig. 3) were early on classified as “Dianthussect.Plumaria”, a name published by Opiz (1852), albeit a nomen nudum and therefore invalid. It largely corresponds to D.sect.Fimbriati (Boiss.) F.N.Williams. This section is highly polyphyletic, and points to multiple evolutionary origins of plumose petals in *Dianthus*, such as in *D.superbus* (core clade A) and *D.crinitus* or *D.libanotis*, (core clade B), and there are also transitional petals slightly plumose at the tip (e.g. *D.cyprius*, *D.namaensis*).

### ﻿Implications for biogeography and diversification patterns

A striking biogeographic feature within *Dianthus* is the Cretan *D.juniperinus*-*D.fruticosus* clade (clade C). The highly fragmented form of the Aegean region with many islands is the result of a series of geological events. Between the Lower Oligocene and before the Serravallian, there was a land mass, Aegea. Since Serravallian and until Upper Tortonian times (12–5 MYA), the sea slowly invaded and formed the mid-Aegean Trench, which divided the eastern and central-western parts of Aegea. During the Messinian Salinity Crisis (5.96–5.33 MYA), the Mediterranean Sea almost dried up, creating routes between some isolated areas. Nevertheless, Crete remained isolated from the Cyclades and Peloponnese due to deep trenches with high salinity. At the beginning of the Pliocene (5.3 Ma), the sea level rose again and resulted in a highly fragmented Aegea. Crete was almost submerged and only the peaks of the mountains remained as isolated islands. Since the Middle Pliocene until the upper Pliocene-Lower Pleistocene, the Aegean islands and Crete took their present form, with minor changes. The palaeogeographic history of the Aegean Sea has a major contribution to the biogeographic patterns of all recent taxa of these areas (Sfenthourakis and Triantis 2017). The bisection of *D.fruticosus* into two different clades, with D.fruticosussubsp.fruticosus, D.fruticosussubsp.amorginus and D.fruticosussubsp.rhodius belonging to clade A and the rest of the subspecies nested in the C with *D.juniperinus*, follows the fragmentation of Aegea due to the formation of the Mid-Aegean Trench. Moreover, Crete was connected to the mainland during most of the Miocene and was mostly isolated during the Pliocene, forming its own unique biogeographic patterns. The Aegean region is a biologically very diverse archipelago, but the way in which the islands and plant groups have interacted and evolved is not yet sufficiently understood. There are some recent studies, e.g. Kougioumoutzis et al. (2016) that address the biogeographic studies in the area, but further research is nonetheless crucial.

The tropical and southern African *Dianthus* taxa appear in our clade C within the Eurasian taxa, although, due to lack of resolution, we cannot yet infer the closest relatives of the southern African species. *Dianthus* shows a pattern like other species from the northern hemisphere with African high-mountain clades nested within Eurasian clades, as shown for, e.g., *Carex* and *Ranunculus* (Gehrke and Linder 2009). Many cases suggest not only migration from the northern hemisphere, but the recurrence of that (Bleeker et al. 2002; Carlsen et al. 2009), which could explain the presence of the African taxa within the clades of Eurasian taxa. The tropical-alpine and tropical-montane floras, as suggested by Linder (2014), show strong relations with the flora of Eurasia. One probable migration route from Eurasia to southern Africa for these taxa is through the Arabian Peninsula and the high mountains of eastern and southern Africa (Koch et al. 2006), which fits well with the pattern observed in this study that African *Dianthus* are part of a clade dominated by the Eurasian and Irano-Turanian taxa.

*Dianthus* is suggested to be one of the fastest species radiations of flowering plants (Valente et al. 2010). These authors calculated diversification rates by using a rather conservative estimate of 300 species. Considering that the number of species in *Dianthus* as assessed here is distinctly higher, the true speciation rates may also even be higher. On the other hand, there is not a single “Eurasian radiation” as suggested by Valente et al. (2010). Rather, our data point to three sub-radiations in the core of *Dianthus*, which seem to have evolved different numbers of species in different geographic areas. It is noteworthy that *Dianthus* has not evolved a high number of species in tropical and southern Africa. The smallest is the radiation of *D.juniperinus* and allies on Crete (our clade C). However, the number of species in this lineage is still difficult to assess because the current classification includes many subspecies and because species as currently classified do not represent biological entities.

Compared to the other lineages of *Dianthus*, clade C shows a high phylogenetic structure and apparently some geographic patterns. Future analyses will therefore test how far speciation was triggered through geographic and ecological isolation on Crete. The two much more species-rich clades A and B have colonised vast geographic areas, offering many more ecological opportunities and potential areas for spatial isolation.

### ﻿Phylogenetic signal at species level and speciation in *Dianthus*

Genetic distances in *Dianthus* are very low, which limits resolution in phylogeny reconstruction at species level and may be attributed to the core clade of *Dianthus* representing a rapid radiation (Valente et al. 2010) that did not yet allow for the accumulation of mutations in the genomic regions sequenced, while the evolution of phenotypic characters occurred more quickly. Sequence divergence is particularly low in the ribosomal array (Fig. 2). Nevertheless, the overall phylogenetic tree of *Dianthus* (Fig. 2) reveals deep nodes with significant statistical support. The core *Dianthus* clade exhibits a deep sharing of plastid haplotypes in some species, for example in *D.gratianopolitanus*, where individuals from geographically different populations in central Europe are resolved in various sublineages of clade A. The pattern in this species is particularly striking since there were no noticeable morphological differences among these individuals. Another species complex with deeply shared plastid haplotypes is *D.orientalis* Adams (Fig. 1, clade B), although here considerable morphological variation is present that has led to the acceptance of several subspecies. Again, the haplotypes are shared only within subclade C, and not across the genus. Such a pattern could be explained by a combination of shared ancestral variation and continuous secondary genetic contact, which took place within certain major lineages of *Dianthus* and within certain geographic areas. *Dianthus* seems to be one of the most extreme cases of deep chloroplast-sharing in flowering plants, similar to what has been observed in the rapid postglacial evolution of relatives of *Arabidopsisthaliana* (L.) Heynh. during the last 800,000 years (Hohmann and Koch 2017; Novikova et al. 2016).

Future research will therefore have to employ phylogenomic approaches to better understand species-level relationships and species limits in *Dianthus*. In addition to sequencing a broad set of nuclear loci, the analysis of complete plastid genomes will reveal how far plastid genomes are really shared between extant species, considering that our current limited sampling of the plastid regions may not depict the full complexity and divergence patterns of this maternally inherited organellar genome.

### ﻿Species diversity of *Dianthus*

Our *Dianthus* checklist contains 384 accepted species, and 147 accepted heterotypic subspecies. The unresolved names section of the checklist contains a further 172 species names that were not assessed, and a number of them may be good species as well. Therefore, the often-quoted number of 300 species (Pax and Hoffmann 1934; Bittrich 1993; Hernández-Ledesma et al. 2015) is underestimated. It should be noted that we present a compilation of names, not a genus-wide treatment, which we nevertheless consider to be an accurate estimate of the species diversity since it is based on available detailed treatments. It is therefore much more accurate compared to the previous World Flora Online backbone based on The Plant List in terms of accepted species and synonyms. About 50 names that were unresolved in the original WFO backbone could be resolved in our checklist. The remaining 172 unresolved names are in part old names, described in the 19^th^ century and apparently not used in subsequent treatments. But there are also a number of names described from south-eastern Europe, which would have to be assessed in more detail by local experts.

It is both noteworthy and unexpected that taxon concepts are congruent between different treatments, despite some geographic overlap. The widely distributed species are accepted under the same name in different Floras, while subspecies are normally geographically restricted and therefore only accepted in one Flora. In total, differing taxonomic concepts affect fewer than 10 taxa in the whole checklist.

So far, only one species complex within *Dianthus* has been analysed with an evolutionary approach. Farsi et al. (2013) used a combination of sequence data and morphological characters to assess species limits in the *D.polylepis* Bien. complex, concluding that *D.binaludensis* Rech.f. could not be separated from *D.polylepis* but should be better treated as its subspecies. Our present study provides insights into the *Dianthusfruticosus* and *D.juniperinus* groups, but aside from that, basically all other species limits in *Dianthus* are still based on classical morphology, and so are the numerous recent descriptions of new species.

### ﻿Nomenclatural novelties

The three species of *Petrorhagia* that were found nested in *Dianthus* (*P.armerioides*, *P.candica* and *P.alpina*) were already transferred to *Dianthus* by Madhani et al. (2018). We found two additional *Petrorhagia* species to be part of the *Dianthus* clade although they have no name in *Dianthus*. Of the three subspecies of *P.illyrica*, we sampled only one. They all are morphologically very similar (Ball and Heywood 1964) and therefore can be expected to be closely related, although we are aware that is not true for the subspecies of *Dianthusfruticosus* that we showed to be unrelated. Nevertheless, we provide new combinations for the other two *P.illyrica* subspecies so that these subspecies can be classified in *Dianthus*. Their phylogenetic placement remains to be tested.

*Veleziarigida*, the nomenclatural type of *Velezia*, was found to be nested within the *Dianthus* clade by Madhani et al. (2018) and therefore Velezia cannot be accepted as a separate genus any longer. We therefore provide new names in *Dianthus* for the five remaining *Velezia* species; their phylogenetic placement however remains to be confirmed.

The complete homotypic and heterotypic synonymy for each of the species is given in the checklist; here we provide only the basionyms and the former names in *Petrorhagia* or *Velezia*.

#### ﻿ Dianthus antalyensis

Taxon classificationPlantaeCaryophyllalesCaryophyllaceae

Fassou, N.Korotkova, Dimop. & Borsch nom. nov.

0406DBE9-336D-5C11-9AA9-18462B9763D0

urn:lsid:ipni.org:names:77297792-1

≡ Velezia tunicoides P.H.Davis in Notes Roy. Bot. Gard. Edinburgh 22: 166. 1957.

##### Holotype.

Turkey, Prov. Antalya, distr. Kemer (Lycia): Gönük, by dry stream bed, 7 Jul 1949, *Davis*, *Bilger & Attila D. 15009* (K-000077456!; isotype: E-00301891!).

##### Note.

The name *Dianthustunicoides* Madhani & Heubl in Taxon 67(1): 103. 2018) was already published as a new name for *Gypsophilaarmerioides* Ser. ex DC. ≡ *Petrorhagiaarmerioides* (Ser. ex DC.) P.W.Ball & Heywood. This new name refers to the province of Antalya, Turkey, from where the species was described.

#### ﻿ Dianthus hispidus

Taxon classificationPlantaeCaryophyllalesCaryophyllaceae

(Boiss. & Balansa) Fassou, N.Korotkova, Dimop. & Borsch comb. nov.

B4D92A99-EBC9-540F-B4CF-24EDA543DAD3

urn:lsid:ipni.org:names:77297793-1

≡ Velezia hispida Boiss. & Balansa in Boissier, Diagn. Pl. Orient., ser. 2, 5: 57. 1856.

##### Syntypes.

Turkey, Yaïla de Bozdagh (Tmolus occidental), dans les vergers, 27 Jul 1854, *Balansa 117* (GOET-000717!, P-04998030!, P-04998031!, P-04998036!, WAG-0000421!).

#### ﻿ Dianthus illyricus

Taxon classificationPlantaeCaryophyllalesCaryophyllaceae

(Ard.) Fassou, N.Korotkova, Dimop. & Borsch comb. nov.

1DD5943C-78F0-5A79-9E70-48FAB7FA2823

urn:lsid:ipni.org:names:77297794-1

≡ Saponaria illyrica Ard., Animadv. Bot. Spec. Alt.: 24. 1764 ≡ Petrorhagiaillyrica (Ard.) P.W.Ball & Heywood in Bull. Brit. Mus. (Nat. Hist.), Bot. 3: 133. 1964.

##### Lectotype (designated here).

locality and date unknown, *Arduino s.n.*, Herb. Linnaeus No. 580.7 (LINN!). This specimen was mentioned by Ball and Heywood (1964). The sheet has two distinct specimens, one of which is labelled “Ard.”, and the typification refers to this specimen. Ball & Heywood mentioned that it “may be regarded at least as an isotype”, but did not formally designate it as a type.

### ﻿Dianthusillyricussubsp.illyricus

#### ﻿ Dianthus illyricus subsp. angustifolius

Taxon classificationPlantaeCaryophyllalesCaryophyllaceae

(Poir.) Fassou, N.Korotkova, Dimop. & Borsch comb. nov.

6FA7F249-0419-512F-8956-4C39C1E00CBE

urn:lsid:ipni.org:names:77297795-1

≡ Silene angustifolia Poir., Voy. Barbarie 2: 164. 1789 ≡ Petrorhagiaillyricasubsp.angustifolia (Poir.) P.W.Ball & Heywood in Bull. Brit. Mus. (Nat. Hist.), Bot. 3: 136. 1964.

##### Neotype (designated here).

locality and date unknown, *Poiret 32* (P-00286897!). This specimen might be original material for the name.

#### ﻿ Dianthus illyricus subsp. haynaldianus

Taxon classificationPlantaeCaryophyllalesCaryophyllaceae

(Nyman) Fassou, N.Korotkova, Dimop. & Borsch comb. nov.

1CF70B19-F9AD-59F0-B088-6E96DC57BBC3

urn:lsid:ipni.org:names:77297796-1

≡ Tunica haynaldiana [Borbás in Mat. Term. Közlem. 13: 46. 1877, pro. syn; in Just's Bot. Jahresber. 4(2): 1067. 1878, provisional name] Nyman, Consp. Fl. Eur. Suppl. 2(1): 57. 1889 ≡ T.illyricavar.haynaldiana (Nyman) Hayek, Prodr. Fl. Penins. Balcan. 1: 222. 1924 ≡ Petrorhagiaillyricasubsp.haynaldiana (Nyman) P.W.Ball & Heywood in in Bull. Brit. Mus. (Nat. Hist.), Bot. 3: 134. 1964.

##### Type:

Romania, in rupestribus versus portam ferram ad Danubium inferiorem infra Orsova jam in Vallachia sitis frequens, 28 Jun 1870, Janka #Iter banaticum s.n. (B-100365631!).

#### ﻿ Dianthus illyricus subsp. taygeteus

Taxon classificationPlantaeCaryophyllalesCaryophyllaceae

(Boiss.) Fassou, N.Korotkova, Dimop. & Borsch comb. nov.

A66FA5CC-1EAB-5D8F-A27A-95519C7887DC

urn:lsid:ipni.org:names:77297799-1

≡ Tunica illyrica var. taygetea Boiss., Fl. Orient. 1: 521. 1867 ≡ Petrorhagiaillyricasubsp.taygetea (Boiss.) P.W.Ball & Heywood in Bull. Brit. Mus. (Nat. Hist.), Bot. 3: 137. 1964.

##### Syntypes.

Greece, Peloponnisos, Androuvista, Montis Taygeti, Jun-Jul 1844, *Heldreich s.n.*, (G-00227046!, G-00227047!, BR0000006981167!, BR0000006981495!, BM, CGE, K fide Ball and Heywood 1964).

#### ﻿ Dianthus fasciculatus

Taxon classificationPlantaeCaryophyllalesCaryophyllaceae

(Boiss.) Fassou, N.Korotkova, Dimop. & Borsch comb. nov.

FCF8874C-6D12-5EC0-B3FF-343EF9CA7C9F

urn:lsid:ipni.org:names:77297800-1

≡ Velezia fasciculata Boiss., Diagn. Pl. Orient., ser. 1, 8: 92. 1849.

##### Syntypes.

Syria, locality as given in the protologue: “Hab. in montosis Syriae borealis inter fluvium Orontem et urbem Laodiceam”, Jun 1846, *Boissier s.n.* (K-000077457!, K-000077458!, LECB-0000587!, P-04998034!, P-04998035!).

##### Note.

The name *Dianthusfasciculatus* Gilib., Fl. Lit. Inch. 2: 161. 1782 was not validly published.

#### ﻿ Dianthus pachygonus

Taxon classificationPlantaeCaryophyllalesCaryophyllaceae

(Fisch & C.A.Mey.) Fassou, N.Korotkova, Nersesian & Borsch comb. nov.

5D688642-5515-5553-8139-F6D45F3643EC

urn:lsid:ipni.org:names:77297801-1

≡ Tunica pachygona Fisch. & C.A.Mey., Index Sem. Hort. Bot. Petropol. 4: 50. 1838
= Saponaria cretica L., Sp. Pl., ed. 2, 1: 584. 1762 ≡ Petrorhagiacretica (L.) P.W.Ball & Heywood in Bull. Brit. Mus. (Nat. Hist.), Bot. 3: 142. 1964.

##### Type.

not designated

##### Lectotype.

(designated by Davis in Notes Roy. Bot. Gard. Edinburgh 22: 164. 1957): locality, date and collector unknown, Herb. Linnaeus No. 580.4 (LINN!).

Davis (1957) had pointed out the name *Tunicacretica* (L.) Fisch. & C.A.Mey. (basionym: *Saponariacretica* L.) had been misapplied, whereas in fact the type of the name (the specimen in LINN) is an annual species that was later named *Tunicapachygona* Fisch. & C.A.Mey. Accordingly, the correct name in *Dianthus* would be *D.pachygonus*, the new combination published here because the epithet of *Saponariacretica* is preoccupied in *Dianthus* by *D.creticus* Tausch. which is now treated as Dianthusfruticosussubsp.creticus (Tausch) Runemark.

#### ﻿ Dianthus pseudorigidus

Taxon classificationPlantaeCaryophyllalesCaryophyllaceae

(Hub.-Mor.) Fassou, N.Korotkova, Dimop. & Borsch comb. nov.

0E6D5105-B0B3-58D3-ACCB-C25DBA73BFA6

urn:lsid:ipni.org:names:77297802-1

≡ Velezia pseudorigida Hub.-Mor. in Bauhinia 2: 195. 1963.

##### Holotype.

Turkey, Prov. Antalya, Distr. Antalya, *Poterium*-*Cistus*-Macchie in der Bucht von Atbükü, 5 km nördlich von Cirali, 10–50 m, auf Serpentin, 26 May 1950, *Huber-Morath 9666* (E 00301890!).

#### ﻿ Dianthus quadridentatus

Taxon classificationPlantaeCaryophyllalesCaryophyllaceae

(Sm.) Fassou, N.Korotkova, Dimop. & Borsch comb. nov.

BB11F84C-80D7-5771-97C3-B13A9533DEF2

urn:lsid:ipni.org:names:77297803-1

≡ Velezia quadridentata Sm. in Sibthorp & Smith, Fl. Graec. Prodr. 1: 283. 1809.

##### Type.

not designated; original material: “In Asiâ minori”, *Sibthorp s.n.* (OXF Sib-FG Sib-0954!, K000077455!).

## ﻿Checklist

***Dianthus* L.﻿, Sp. Pl. 1: 409. 1753.** Sec. this publication

≡ *Caryophyllus* Mill.﻿, Gard. Dict. Abr., ed. 4: [textus s.n.]. 1754, nom. illeg. syn. sec. IPNI

≡ *Caryophyllus* Tourn. ex Moench﻿, Methodus: 58. 1794 syn. sec. POWO. Plants of the World Online. Facilitated by the Royal Botanic Gardens, Kew.

= *Velezia* L.﻿, Sp. Pl.: 332. 1753 syn. sec. Madhani et al. (2018). Type: *Veleziarigida* L.﻿

= *Cylichnanthus* Dulac﻿, Fl. Hautes-Pyrénées: 260. 1867 syn. sec. Madhani et al. (2018)

– *Diosanthos* St.-Lag.﻿ in Ann. Soc. Bot. Lyon 7: 87. 1880, nom. inval. syn. sec. Kew WCVP (2019)

### ﻿Core checklist

***Dianthusacantholimonoides* Schischk.﻿ in Byull. Gosud. Muz. Gruzii 5: 121. 1930.** Sec. Kuzmina & Nersesyan (2012)

***Dianthusacicularis* Fisch. ex Ledeb.﻿, Fl. Ross. 1: 284. 1842.** Sec. Czerepanov (1995)

= *Dianthustauscheri* Eversm. ex Ledeb.﻿, Fl. Ross. 1: 284. 1842 syn. sec. Dequan & Turland (2001)

***Dianthusacrochlorus* Stapf﻿ in Denkschr. Kaiserl. Akad. Wiss., Wien. Math.-Naturwiss. Kl. 51: 5. 1886.** Sec. Marhold (2011)

– *Dianthusacrochlonis* Stapf﻿ in Denkschr. Kaiserl. Akad. Wiss., Wien. Math.-Naturwiss. Kl. 51: 5. 1886 syn. sec. Marhold (2011) [is misspelling for *Dianthusacrochlorus* Stapf﻿]

***Dianthusaculeatus* Hamzaoğlu﻿ in Biol. Diversity Conservation 7(2): 160. 2014.** Sec. Hamzaoğlu et al. (2014)

***Dianthusafghanicus* Rech.f.﻿ in Bot. Jahrb. Syst. 75: 360. 1951.** Sec. Rechinger (1988)

***Dianthusagrostolepis* Rech.f.﻿ in Plant Syst. Evol. 142: 246. 1983.** Sec. Kuzmina & Nersesyan (2012)

***Dianthusakdaghensis* Gemici & Leblebici﻿ in Candollea 50: 43. 1995.** Sec. Gemici & Leblebici (1995)

***Dianthusalbens* Aiton﻿, Hort. Kew. 2: 90. 1789.** Sec. African Plant Database (version 3.4.0)

= *Dianthusincurvus* Thunb.﻿, Prodr. Pl. Cap. 1: 81. 1794 syn. sec. African Plant Database (version 3.4.0)

***Dianthusalgetanus* Graells ex F.N.Williams﻿ in J. Bot. 23: 347. 1885.** Sec. Bernal et al. (1990)

≡ Dianthuspyrenaicussubsp.algetanus (Graells ex F.N.Williams) Malag.﻿, Sin. Fl. Ibér. 20: 318. 1975 syn. sec. Bernal et al. (1990) ≡ Dianthuscostaesubsp.algetanus (Graells ex F.N.Williams) M.Laínz, Muñoz Garm. & Soriano﻿ in Anales Jard. Bot. Madrid 43: 473. 1986 [“1987”] syn. sec. Bernal et al. (1990)

= Dianthusalgetanusvar.toletanorum Pau﻿ in Cavanillesia 1: 63. 1928 syn. sec. Bernal et al. (1990)

**Dianthusalgetanussubsp.algetanus**﻿

**Dianthusalgetanussubsp.turolensis (Pau) M.Bernal, Laínz & Muñoz Garm.﻿ in Anales Jard. Bot. Madrid 45: 575. 1988 [“1989”].** Sec. Bernal et al. (1990)

≡ *Dianthusturolensis* Pau﻿ in Bol. Real Soc. Esp. Hist. Nat. 21: 142. 1921 syn. sec. Bernal et al. (1990) ≡ Dianthusalgetanusvar.turolensis (Pau) Pau﻿ in Bortéria, Sér. Bot. 22: 111. 1926 syn. sec. Bernal et al. (1990) ≡ Dianthuscostaesubsp.turolensis (Pau) M.Laínz & Muñoz Garm﻿ in Anales Jard. Bot. Madrid 43: 473. 1986 [“1987”] syn. sec. Bernal et al. (1990)

***Dianthusalpinus* L.﻿, Sp. Pl.: 412. 1753.** Sec. Marhold (2011)

= *Dianthusalpinus* lusus *angustifolius* Regel﻿ in Bull. Soc. Imp. Naturalistes Moscou 34(2): 530. 1862 syn. sec. Marhold (2011)

= *Dianthusalpinus* lusus *latifolius* Regel﻿ in Bull. Soc. Imp. Naturalistes Moscou 34(2): 530. 1862 syn. sec. Marhold (2011)

= Dianthusalpinusvar.meyeri Regel﻿ in Bull. Soc. Imp. Naturalistes Moscou 34(2): 530. 1862 syn. sec. Kew WCVP (2019)

***Dianthusaltaicus* L.X.Dong & Chang Y.Yang﻿ in Acta Bot. Boreal.-Occid. Sin. 28(12): 2355. 2008.** Sec. Dong et al. (2008)

***Dianthusamurensis* Jacques﻿ in J. Soc. Imp. Centr. Hort. 7: 625. 1861.** Sec. Barkalov & Probatova (2006)

≡ Dianthuschinensisvar.amurensis (Jacques) Kitag.﻿, Neo-Lineam. Fl. Manshur.: 266. 1979 syn. sec. this publication

***Dianthusanatolicus* Boiss.﻿, Diagn. Pl. Orient. ser. 1 1: 22. 1843.** Sec. Marhold (2011)

= *Dianthusparviflorus* Boiss.﻿, Diagn. Pl. Orient. ser. 1 1: 21. 1843 syn. sec. Marhold (2011)

≡ Dianthusanatolicusvar.parviflorus (Boiss.) Boiss.﻿, Fl. Orient. 1: 490. 1867 syn. sec. Dimopoulos et al. (2013)

= *Dianthuskotschyanus* Boiss. & Heldr.﻿ in Boissier, Diagn. Pl. Orient., ser. 1, 8: 68. 1849 syn. sec. Marhold (2011)

***Dianthusancyrensis* Hausskn. & Bornm.﻿ in Repert. Spec. Nov. Regni Veg. Beih. 89: 94. 1936.** Sec. Marhold (2011)

***Dianthusandronakii* Woronow ex Schischk.﻿, Fl. URSS 6: 841. 1936.** Sec. Kuzmina & Nersesyan (2012)

≡ *Dianthustristis* Woronow﻿ in Vĕstn. Tiflissk. Bot. Sada 10: 25. 1908 syn. sec. WFO 2018

***Dianthusandrosaceus* (Boiss. & Heldr.) Hayek﻿ in Kaiserl. Akad. Wiss. Wien, Math.-Naturwiss. Kl., Denkschr. 94: 141. 1918.** Sec. Dimopoulos et al. (2013)

≡ Dianthuslilacinusvar.androsaceus Boiss. & Heldr.﻿ in Boissier, Fl. Orient. Suppl.: 81. 1888 syn. sec. WFO 2018

***Dianthusandrzejejowskianus* (Zapał.) Kulcz.﻿, Fl. Polska 2: 156. 1921.** Sec. Czerepanov (1995)

≡ Dianthuscapitatussubsp.andrzejowskianus Zapał.﻿ in Rozpr. Wydz. Mat.-Przyr Polsk Akad. Umiejetn., Dzial A/B, Nauki Mat-Fiz. Biol. 11: 25. 1911 syn. sec. Czerepanov (1995)

= Dianthuscapitatusvar.pancicianus F.N.Williams﻿ in J. Bot. 23: 342. 1885 syn. sec. Kew WCVP (2019)

= Dianthusandrzejejowskianussubsp.orientalis Kleopow﻿ in Izv. Kievsk. Bot. Sada 14: 104. 1932 syn. sec. Kew WCVP (2019)

***Dianthusangolensis* Hiern ex F.N.Williams﻿ in J. Bot. 24: 301. 1886.** Sec. African Plant Database (version 3.4.0)

***Dianthusangrenicus* Vved.﻿ in Bot. Mater. Gerb. Bot. Inst. Uzbekistansk. Fil. Akad. Nauk S.S.S.R. 3: 9. 1941.** Sec. WFO 2018

***Dianthusangulatus* Royle ex Benth.﻿, Ill. Bot. Himal. Mts.: 79. 1834.** Sec. Rechinger (1988)

≡ Dianthusorientalisvar.angulatus (Royle ex Benth.) Majumdar﻿, Fl. India 2: 532. 1993 syn. sec. WFO 2018

= *Dianthusincertus* Jacquem. ex Edgew. & Hook.f.﻿, Fl. Brit. India 1: 215. 1874 syn. sec. WFO 2018

**Dianthusangulatussubsp.angulatus**﻿

**Dianthusangulatussubsp.subangulatus Rech.f.﻿, Fl. Iranica 163: 183. 1988.** Sec. Rechinger (1988)

***Dianthusantalyensis* Fassou, N.Korotkova, Dimop. & Borsch﻿.** Sec. this publication 118

≡ *Veleziatunicoides* P.H.Davis in Notes Roy. Bot. Gard. Edinburgh 22: 166. 1957. Syn. sec. this publication

***Dianthusanticarius* Boiss. & Reut.﻿, Pugill. Pl. Afr. Bor. Hispan.: 19. 1852.** Sec. Bernal et al. (1990)

≡ Dianthuscintranussubsp.anticarius (Boiss. & Reut.) Malag.﻿, Pl. Sennen. I: Dianthus 6. 1974 syn. sec. Bernal et al. (1990)

= *Dianthusgaditanus* Boiss.﻿, Diagn. Pl. Orient., ser. 2, 1: 67. 1854 syn. sec. Bernal et al. (1990)

= *Dianthushornemannii* Salzm. ex Boiss.﻿, Diagn. Pl. Orient., ser. 2, 1: 67. 1854 syn. sec. Marhold (2011)

= *Dianthusschousboei* Coss. ex Ball﻿ in J. Linn. Soc., Bot. 16: 355. 1877 syn. sec. WFO 2018

**Dianthusanticariussubsp.anticarius**﻿

**Dianthusanticariussubsp.saorinii Sánchez-Gómez, M.L.Rodr., López Esp., J.B.Vera & J.F.Jiménez﻿ in Anales Biol., Fac. Biol., Univ. Murcia 27: 101. 2005.** Sec. New taxa described to the Flora Iberica region after publication of the respective volumes. Published at http://www.floraiberica.es/eng/miscelania/nuevos_taxones.php

***Dianthusarenarius* L., Sp. Pl.: 412. 1753.** Sec. Czerepanov (1995)

≡ *Tunicaarenaria* (L.) Scop.﻿, Fl. Carniol., ed. 2, 1: 301. 1771 ≡ *Silenearenaria* (L.) E.H.L.Krause﻿, Deutschl. Fl. Abbild., ed. 2, 5: 115. 1901, nom. illeg. syn. sec. Kew WCVP (2019);

= Dianthusarenariusvar.glaucus Blocki﻿ in Oesterr. Bot. Z. 34: 72. 1884 syn. sec. Kew WCVP (2019)

= *Dianthuskrylovianus* Juz.﻿ in Bot. Mater. Gerb. Bot. Inst. Komarova Akad. Nauk S.S.S.R. 13: 71. 1950 syn. sec. Czerepanov (1995)

= Dianthusarenariusvar.suecicus Novák﻿ syn. sec. Czerepanov (1995)

**Dianthusarenariussubsp.arenarius**﻿

**Dianthusarenariussubsp.borussicus Vierh.﻿ in Izv. Kievsk. Bot. Sada 12–13: 34. 1931.** Sec. Marhold (2011)

≡ *Dianthusborussicus* (Vierh.) Juz.﻿ syn. sec. Marhold (2011)

= Dianthusarenariusvar.bohemicus Novák﻿ syn. sec. Kew WCVP (2019) ≡ Dianthusarenariussubsp.bohemicus (Novák) O.Schwartz﻿ in Mitt. Thüring. Bot. Ges. 1: 99. 1949 syn. sec. Kew WCVP (2019)

**Dianthusarenariussubsp.pseudoserotinus (Blocki) Tutin﻿ in Feddes Repert. Spec. Nov. Regni Veg. 68: 190. 1963.** Sec. Czerepanov (1995)

≡ *Dianthuspseudoserotinus* Blocki﻿ syn. sec. Czerepanov (1995) ≡ Dianthusserotinusvar.pseudoserotinus (Blocki) Zapał.﻿, Consp. Fl. Gallic. Crit. 3: 150. 1911 syn. sec. Kew WCVP (2019)

**Dianthusarenariussubsp.pseudosquarrosus (Novák) Kleopow﻿ in Izv. Kievsk. Bot. Sada 12–13: 35. 1931.** Sec. Marhold (2011)

≡ Dianthusarenariusf.pseudosquarrosus Novák﻿ in Mem. Soc. Sci. De Bohem 1: 9. 1925 syn. sec. Kew WCVP (2019) ≡ *Dianthuspseudosquarrosus* (Novák) Klokov﻿, Fl. RSS Ucr. 4: 639. 1952 syn. sec. Marhold (2011)

***Dianthusaristatus* Boiss.﻿, Asie Min., Bot. 1: 222. 1860.** Sec. Kuzmina & Nersesyan (2012)

≡ Dianthuszonatusvar.aristatus (Boiss.) Reeve﻿, Notes Roy. Bot. Gard. Edinb. 28: 21. 1967 syn. sec. Kuzmina & Nersesyan (2012)

= *Dianthuspreobrazhenskii* Klokov﻿ in Trudy Silsko-Gosp. Bot. 1(3): 170. 1927 syn. sec. Kuzmina & Nersesyan (2012)

***Dianthusarmeria* L.﻿, Sp. Pl.: 410. 1753.** Sec. Marhold (2011)

≡ *Caryophyllusarmerius* (L.) Moench﻿, Methodus: 59. 1794 syn. sec. POWO. Plants of the World Online. Facilitated by the Royal Botanic Gardens, Kew. ≡ Dianthusarmeriasubsp.armeria﻿ syn. sec. WFO 2018

= *Dianthushirsutus* Lam.﻿, Fl. Franç. 2: 533. 1779 syn. sec. Bernal et al. (1990)

= *Dianthushirtus* Lam.﻿, Fl. Franç. 2: 533. 1779 syn. sec. WFO 2018

= *Dianthusvillosus* Gilib.﻿, Fl. Lit. Inch. 2: 160. 1782 syn. sec. WFO 2018

= *Dianthuscarolinianus* Walter﻿, Fl. Carol.: 140. 1788 syn. sec. WFO 2018

= Gypsophilaarmeriavar.nanus Boenn.﻿, Prodr. Fl. Monast. Westphal.: 124. 1824 syn. sec. Kew WCVP (2019)

= *Dianthushybridus* F.W.Schmidt ex Tausch﻿ in Flora 13: 245. 1830 syn. sec. WFO 2018

= *Dianthusvivariensis* Jord. ex Boreau﻿, Fl. Centre France ed. 3, 2: 91. 1857 syn. sec. WFO 2018

= Dianthusarmeriavar.laevis Heuff. in Verh. Zool.-Bot. Ges. Wien 8: 68. 1858 syn. sec. Kew WCVP (2019)

= *Dianthusarmeriastrum* Wolfner﻿ in Oesterr. Bot. Z. 8: 318. 1858 syn. sec. WFO 2018 ≡ Dianthusarmeriasubsp.armeria﻿strum (Wolfner) Velen.﻿, Fl. Bulg. Suppl. 1: 42. 1898 syn. sec. WFO 2018

= *Dianthusepirotus* Halácsy﻿ in Verh. K. K. Zool.-Bot. Ges. Wien 48: 708. 1898 syn. sec. WFO 2018

= *Silenevaga* E.H.L.Krause﻿, Deutschl. Fl. Abbild., ed. 2, 5: 109. 1901 syn. sec. POWO. Plants of the World Online. Facilitated by the Royal Botanic Gardens, Kew.

= *Dianthuspseudocorymbosus* Velen.﻿ in Sitzungsber. Königl. Böhm. Ges. Wiss., Math.-Naturwiss. Cl. 8: 6. 1910 [“1911”] syn. sec. Dimopoulos et al. (2013)

= Dianthusarmeriaf.acaulis Bolzon﻿ in Bull. Soc. Bot. Ital. 1911: 56. 1911 syn. sec. Kew WCVP (2019)

= Dianthusarmeriaf.caespitosa Bolzon﻿ in Bull. Soc. Bot. Ital. 1911: 56. 1911 syn. sec. Kew WCVP (2019)

= Dianthusarmeriaf.albiviridis Lehr﻿ in Bull. Torrey Bot. Club 90: 207. 1963 syn. sec. WFO 2018

= Dianthusarmeriaf.glabrissimus Sigunov﻿ in Glasn. Prir. Muz. Beogradu, C 10: 23. 1977 syn. sec. WFO 2018

= Dianthusarmeriaf.alba Stritch﻿ in Castanea 48: 58. 1983 syn. sec. WFO 2018

***Dianthusarpadianus* Ade & Bornm.﻿ in Repert. Spec. Nov. Regni Veg. 36: 385. 1934.** Sec. Dimopoulos et al. (2013)

***Dianthusarrosti* C.Presl﻿, Delic. Prag.: 60. 1822.** Sec. Marhold (2011)

≡ Dianthuscaryophyllusvar.arrosti (C.Presl) Tanfani﻿, Fl. Ital. 9: 283. 1892 syn. sec. WFO 2018 ≡ Dianthuscaryophyllussubsp.arrosti (C.Presl) Arcang.﻿, Comp. Fl. Ital. ed. 2: 306. 1894 syn. sec. Marhold (2011) – *Dianthusarrostii* C.Presl﻿, Delic. Prag.: 60. 1822 syn. sec. Marhold (2011) [is orthographic variant for *Dianthusarrosti* C.Presl﻿]

= *Dianthuscontractus* Jan ex Lojac.﻿, Fl. Sicul. 1: 164. 1889 syn. sec. WFO 2018

**Dianthus×artignanii Sennen﻿ in Bol. Soc. Ibér. Ci. Nat. 25: 145. 1926.** Sec. Bernal et al. (1990)

***Dianthusaticii* Hamzaoğlu﻿ in Phytokeys 48: 22. 2015.** Sec. Hamzaoğlu et al. (2015)

***Dianthusatlanticus* Pomel﻿, Nouv. Mat. Fl. Atl. 1: 332. 1874.** Sec. African Plant Database (version 3.4.0)

≡ Dianthusliburnicusvar.atlanticus (Pomel) Chabert﻿ in Bull. Soc. Bot. France 38: 383. 1892 [“1891”] syn. sec. Kew WCVP (2019)

***Dianthusatschurensis* Sosn.﻿ in Vestn. Tiflissk. Bot. Sada n.s. 1: 74. 1923.** Sec. Kuzmina & Nersesyan (2012)

– *Dianthusazkurensis* Sosn.﻿ in Vestn. Tiflissk. Bot. Sada n.s. 1: 74. 1923 syn. sec. this publication [is misspelling for *Dianthusatschurensis* Sosn.﻿]

***Dianthusaustroiranicus* Lemperg﻿ in Repert. Spec. Nov. Regni Veg. 50: 260. 1941.** Sec. Rechinger (1988)

***Dianthusawaricus* Kharadze﻿ in Zametki Sist. Geogr. Rast. 16: 50. 1951.** Sec. Kuzmina & Nersesyan (2012)

***Dianthusaydogdui* Menemen & Hamzaoğlu﻿ in Ann. Bot. Fenn. 37: 285. 2000.** Sec. Menemen & Hamzaoğlu (2000)

***Dianthusaytachii* C.Vural﻿ in Bot. J. Linn. Soc. 158: 55. 2008.** Sec. Vural (2008)

***Dianthusbalansae* Boiss.﻿, Fl. Orient. 1: 488. 1867.** Sec. Marhold (2011)

***Dianthusbarbatus* L.﻿, Sp. Pl.: 409. 1753.** Sec. Marhold (2011)

≡ *Caryophyllusbarbatus* (L.) Moench﻿, Methodus: 59. 1794 syn. sec. POWO. Plants of the World Online. Facilitated by the Royal Botanic Gardens, Kew. ≡ *Cylichnanthusbarbatus* (L.) Dulac﻿, Fl. Hautes-Pyrénées: 261. 1867 syn. sec. POWO. Plants of the World Online. Facilitated by the Royal Botanic Gardens, Kew. ≡ *Silenebarbata* (L.) E.H.L.Krause﻿, Deutschl. Fl. Abbild., ed. 2, 5: 108. 1901 syn. sec. Kew WCVP (2019); ≡ Dianthusbarbatusvar.barbatus﻿

= *Tunicabarbata* Scop.﻿, Fl. Carniol., ed. 2, 1: 298. 1771 syn. sec. Marhold (2011)

= *Dianthushispanicus* Dum.Cours.﻿ in Bot. Cult. 3: 168. 1802 syn. sec. Kuzmina & Nersesyan (2012)

= *Dianthuslatifolius* Willd.﻿, Enum. Pl.: 466. 1809 syn. sec. Kuzmina & Nersesyan (2012)

= *Dianthusaggregatus* Poir.﻿, Encycl. Suppl. 4: 124. 1816 syn. sec. Kuzmina & Nersesyan (2012)

= *Dianthuscorymbosus* F.Dietr.﻿, Nachtr. Vollst. Lex. Gärtn. 2: 667. 1816 syn. sec. Kuzmina & Nersesyan (2012)

= Dianthusbarbatusvar.latifolius Ser.﻿, Prodr. 1: 356. 1824 syn. sec. Kew WCVP (2019)

= Dianthusbarbatusvar.paniculatus Ser.﻿, Prodr. 1: 356. 1824 syn. sec. Kew WCVP (2019)

= Dianthusbarbatusvar.pedunculosus Ser.﻿, Prodr. 1: 356. 1824 syn. sec. Kew WCVP (2019)

= *Dianthuspulcherrimus* Loisel.﻿, Dict. Sci. Nat. ed. 2, 35: 417. 1825 syn. sec. Kuzmina & Nersesyan (2012)

= *Dianthussplendidissimus* Hoffmanns.﻿ in Verz. Pfl.-Kult. Nachtr: 27. 1842 syn. sec. Kuzmina & Nersesyan (2012)

= *Dianthusgirardinii* Lamotte﻿ in Bull. Soc. Bot. France 21: 120. 1874 syn. sec. Kuzmina & Nersesyan (2012)

= Dianthusbarbatusvar.asiaticus Nakai﻿, Tyosen-Syokubutsu: 143. 1914 syn. sec. Marhold (2011)

**Dianthusbarbatussubsp.barbatus**﻿

**Dianthusbarbatussubsp.compactus (Kit. ex Schult.) Nyman﻿, Consp. Fl. Eur. Suppl. 2(1): 58. 1889.** Sec. Marhold (2011)

≡ *Dianthuscompactus* Kit. ex Schult.﻿, Oestr. Fl. ed. 2, 1: 654. 1814 syn. sec. Marhold (2011) ≡ Dianthusbarbatusvar.compactus (Kit. ex Schult.) Heuff.﻿ in Verh. K. K. Zool.-Bot. Ges. Wien 8: 68. 1858 syn. sec. Kuzmina & Nersesyan (2012)

***Dianthusbasianicus* Boiss. & Hausskn. ex Boiss.﻿, Fl. Orient. Suppl.: 77. 1888.** Sec. Rechinger (1988)

***Dianthusbasuticus* Burtt Davy﻿ in Bull. Misc. Inform. Kew 1922: 220. 1922.** Sec. African Plant Database (version 3.4.0)

= Dianthusmicropetalusvar.galpinii Burtt Davy﻿ syn. sec. WFO 2018

**Dianthusbasuticussubsp.basuticus**﻿

**Dianthusbasuticussubsp.fourcadei S.S.Hooper﻿, Hooker’s Icon. Pl. 7 [1]: 20–22. 1959.** Sec. African Plant Database (version 3.4.0)

**Dianthusbasuticusvar.grandiflorus S.S.Hooper﻿, Hooker’s Icon. Pl. 7 [1]: 19. 1959.** Sec. African Plant Database (version 3.4.0)

***Dianthusbenearnensis* Loret﻿ in Bull. Soc. Bot. France 5: 327. 1858.** Sec. Bernal et al. (1990)

≡ Dianthusfurcatussubsp.benearnensis (Loret) Kerguélen﻿ in Lejeunia 120: 79. 1987 syn. sec. IPNI

***Dianthusbessarabicus* (Kleopow) Klokov﻿ in Bot. Zhurn. (Kiev) 5: 28. 1948.** Sec. Czerepanov (1995)

≡ Dianthuspolymorphussubsp.bessarabicus Kleopow﻿ in Izv. Kievsk. Bot. Sada 14: 114. 1932 syn. sec. Czerepanov (1995)

= Dianthuspolymorphusvar.bessarabicus Sanda﻿ syn. sec. Czerepanov (1995)

***Dianthusbicolor* Adams﻿ in Beitr. Naturk. 1: 55. 1805.** Sec. Kuzmina & Nersesyan (2012)

= *Dianthuscaucasicus* M.Bieb.﻿, Fl. Taur.-Caucas. 1: 327. 1808 syn. sec. Kuzmina & Nersesyan (2012)

= Dianthusbicolorvar.minor Ser.﻿, Prodr. 1: 361. 1824 syn. sec. Kew WCVP (2019)

***Dianthusbiflorus* Sm.﻿, Fl. Graec. Prodr. 1(2): 285. 1809.** Sec. Dimopoulos et al. (2013)

= *Dianthusbinatus* Bartl. ex Rchb.﻿, Fl. Germ. Excurs.: 810. 1832 syn. sec. WFO 2018

= *Dianthuscinnabarinus* Spruner ex Boiss.﻿, Diagn. Pl. Orient. ser. 1, 6: 22. 1846 syn. sec. WFO 2018

= *Dianthussamaritani* Heldr. ex Halácsy, Consp. Fl. Graec. 1: 213. 1900 syn. sec. WFO 2018 ≡ Dianthusbiflorussubsp.samaritanii (Heldr. ex Halácsy) Maire & Petitm﻿ syn. sec. Dimopoulos et al. (2013) – *Dianthussamaritani* Heldr. ex Boiss.﻿, Fl. Orient. 1: 511. 1867, nom. inval. syn. sec. Dimopoulos et al. (2013)

***Dianthusboissieri* Willk.﻿, Icon. Descr. Pl. Nov. 1: 22. 1853.** Sec. Bernal et al. (1990)

≡ Dianthuscaryophyllusvar.boissieri (Willk.) Emb. & Maire﻿, Fl. Afr. Nord 10: 318. 1963 syn. sec. Bernal et al. (1990) ≡ Dianthussylvestrissubsp.boissieri (Willk.) Dobignard﻿ in J. Bot. Soc. Bot. France 20: 37. 2002 syn. sec. WFO 2018

= *Dianthussylvestris* Boiss.﻿, Fl. Orient. 1: 701. 1867 syn. sec. WFO 2018

= Dianthuscaryophyllusvar.brevifolius Rouy﻿, Obs. Dianthus France: 3. 1882 syn. sec. Kew WCVP (2019) ≡ Dianthuscaryophyllusf.brevifolius (Rouy) Maire﻿, Fl. Afrique N. 10: 319. 1963 syn. sec. Kew WCVP (2019)

= Dianthuscaryophyllusvar.longifolius Rouy﻿, Obs. Dianthus France: 3. 1882 syn. sec. Kew WCVP (2019) ≡ Dianthuscaryophyllusf.longifolius (Rouy) Maire﻿, Fl. Afrique N. 10: 319. 1963 syn. sec. Kew WCVP (2019)

= Dianthuscaryophyllusvar.longifolius Maire﻿ in Bull. Soc. His. Nat. Afrique N. 22: 36. 1931, nom. illeg. syn. sec. Kew WCVP (2019)

= *Dianthuscharmelii* Sennen & Mauricio﻿, Diagn. Nouv.: 246. 1936 syn. sec. WFO 2018 ≡ Dianthuscaryophyllusf.charmelii (Sennen & Mauricio) Maire﻿, Fl. Afr. Nord 10: 319. 1963 syn. sec. WFO 2018

= Dianthuscaryophyllusvar.tenuicaulis Maire﻿ in Bull. Soc. His. Nat. Afrique N. 30: 333. 1939 syn. sec. Kew WCVP (2019)

– Dianthuscaryophyllusf.grandiflorus Pau & Font Quer﻿, Iter Marocc. 1928: no. 102. 1829, nom. inval. syn. sec. Kew WCVP (2019)

– Dianthuscaryophyllusf.emancipatus Pau & Font Quer﻿, Iter Marocc. 1927: no. 195. 1928, nom. inval. syn. sec. Kew WCVP (2019)

***Dianthusbolusii* Burtt Davy﻿ in Bull. Misc. Inform. Kew 1922: 218. 1922.** Sec. African Plant Database (version 3.4.0)

***Dianthusborbasii* Vandas﻿ in Oesterr. Bot. Z. 36: 193. 1886.** Sec. Kuzmina & Nersesyan (2012)

***Dianthusborbonicus* Brullo, C.Brullo, Colombo, Giusso, Ilardi & R.Perrone﻿ in Phytotaxa 233: 50. 2015.** Sec. Brullo et al. (2015)

***Dianthusbrachycalyx* A.Huet & É.Huet ex Bacch., Brullo, Casti & Giusso﻿ in Nordic J. Bot. 28: 142. 2010.** Sec. Bacchetta et al. (2010)

***Dianthusbrevicaulis* Fenzl﻿, Pug. Pl. Nov. Syr.: 10. 1842.** Sec. Bacchetta et al. (2010)

**Dianthusbrevicaulissubsp.brevicaulis**﻿

**Dianthusbrevicaulissubsp.setaceus Reeve﻿ in Notes Roy. Bot. Gard. Edinburgh 28: 21. 1967.** Sec. Marhold (2011)

***Dianthusbrevipetalus* Vved.﻿ in Bot. Mater. Gerb. Bot. Inst. Uzbekistansk. Fil. Akad. Nauk S.S.S.R. 3: 9. 1941.** Sec. Czerepanov (1995)

***Dianthusbroteri* Boiss. & Reut.﻿, Pugill. Pl. Afr. Bor. Hispan.: 22. 1852.** Sec. Bernal et al. (1990)

≡ *Dianthusfimbriatus* Brot.﻿, Fl. Lusit. 2: 177. 1805, nom. illeg. syn. sec. Bernal et al. (1990)

= Dianthusserrulatusvar.barbatus Boiss.﻿, Elench. Pl. Nov.: 19. 1838 syn. sec. Bernal et al. (1990) ≡ Dianthusserrulatussubsp.barbatus (Boiss.) Greuter & Burdet﻿ in Willdenowia 13: 281. 1983 [“1984”] syn. sec. Bernal et al. (1990)

= *Dianthusvalentinus* Willk.﻿, Icon. Descr. Pl. Nov. 1: 14. 1852 syn. sec. Bernal et al. (1990) ≡ Dianthusmalacitanusvar.valentinus (Willk.) Losa & Rivas Goday﻿ in Arch. Inst. Aclim. Cons. Super. Invest. Ci. 13(2): 150. 1974 syn. sec. Bernal et al. (1990) ≡ Dianthusbroterisubsp.valentinus (Willk.) Rivas Mart. et al.﻿ in Rivasgodaya 6: 29. 1991 syn. sec. Bernal et al. (1990)

= *Dianthusmalacitanus* Haens. ex Boiss.﻿, Fl. Orient. 1: 85. 1867 syn. sec. Bernal et al. (1990)

= *Dianthusserrulatus* Boiss.﻿, Fl. Orient. 1: 84. 1867 syn. sec. Bernal et al. (1990)

= *Dianthuseusebii* Sennen﻿, Diagn. Nouv.: 264. 1936 syn. sec. Bernal et al. (1990)

= *Dianthusabsconditus* Fern.Casas﻿ in Fontqueria 3: 35. 1983 syn. sec. WFO 2018

= *Dianthussubbaeticus* Fern.Casas﻿ in Fontqueria 3: 37. 1983 syn. sec. WFO 2018 ≡ Dianthusbroterisubsp.subbaeticus (Fern.Casas) Fern.Casas, M.Laínz & Muñoz Garm.﻿ in Anales Jard. Bot. Madrid 44: 573. 1987 syn. sec. Bernal et al. (1990) ≡ Dianthusanticariussubsp.subbaeticus (Fern.Casas) Rivas Mart. et al.﻿ in Rivasgodaya 6: 28. 1991 syn. sec. Bernal et al. (1990)

= *Dianthushinoxianus* Gallego﻿ in Lagascalia 14: 71. 1986 syn. sec. Bernal et al. (1990) ≡ Dianthusbroterisubsp.hinoxianus (Gallego) Rivas Mart.﻿ in Lagascalia 15(Extra): 116. 1988 syn. sec. Bernal et al. (1990)

***Dianthusbrutius* Brullo, Scelsi & Spamp.﻿, Portugaliae Act. Biol., Sér. B, Sist. 19: 304. 2000.** Sec. Brullo et al. (2000)

**Dianthusbrutiussubsp.brutius**﻿

**Dianthusbrutiussubsp.pentadactyli Brullo, Scelsi & Spamp., Portugaliae Act. Biol., Sér. B, Sist. 19: 306. 2000.** Sec. Brullo et al. (2000)

***Dianthusbukovinensis* (Zapał.) Klokov﻿, Fl. RSS Ucr. 4: 606. 1952.** Sec. Czerepanov (1995)

≡ Dianthuscarthusianorumvar.bucovinensis Zapał.﻿, Consp. Fl. Gallic. Crit. 3: 113. 1911 syn. sec. WFO 2018 – *Dianthusbucoviensis* (Zapał.) Klokov﻿ syn. sec. WFO 2018 [is orthographic variant for *Dianthusbukovinensis* (Zapał.) Klokov﻿] – Dianthuscarthusianorumvar.bucoviensis Zapał.﻿ syn. sec. Czerepanov (1995) [is orthographic variant for Dianthuscarthusianorumvar.bucovinensis Zapał.]

***Dianthusburchellii* Ser.﻿, Prodr. 1: 359. 1824.** Sec. African Plant Database (version 3.4.0)

***Dianthusburdurensis* Hamzaoğlu & Koç﻿ in Phytotaxa 233: 197. 2015.** Sec. Hamzaoğlu & Koç (2015)

***Dianthusbusambrae* Soldano & F.Conti﻿, Annot. Checkl. Italian Vasc. Fl.: 18. 2005.** Sec. Bacchetta et al. (2010)

= *Dianthuspaniculatus* Lojac.﻿ in Malpighia 20: 188. 1906 syn. sec. Bacchetta et al. (2010)

***Dianthuscachemiricus* Edgew. & Hook.f.﻿, Fl. Brit. India 1: 214. 1874.** Sec. Rechinger (1988)

***Dianthuscaespitosus* Thunb.﻿, Prodr. Pl. Cap. 1: 81. 1794.** Sec. African Plant Database (version 3.4.0)

**Dianthuscaespitosussubsp.caespitosus**﻿

**Dianthuscaespitosussubsp.pectinatus (E.Mey. ex Sond.) S.S.Hooper﻿ in Hookers Icon. Pl. 37: 37. 1959.** Sec. African Plant Database (version 3.4.0)

≡ *Dianthuspectinatus* E.Mey. ex Sond.﻿, Fl. Cap. 1: 124. 1860 syn. sec. African Plant Database (version 3.4.0)

= *Dianthusprostratus* Jacq.﻿, Pl. Hort. Schoenbr. 3: 11. 1798 syn. sec. African Plant Database (version 3.4.0)

= *Dianthusalbens* Eckl. & Zeyh.﻿, Enum. Pl. Afric. Austral. 1: 32. 1835 syn. sec. WFO 2018

= *Dianthuscrenatus* S.T.Edw.﻿ syn. sec. African Plant Database (version 3.4.0)

***Dianthuscallizonus* Schott & Kotschy﻿ in Bot. Zeitung (Berlin) 9: 192. 1851.** Sec. Marhold (2011)

***Dianthuscalocephalus* Boiss.﻿, Diagn. Pl. Orient. ser. 1, 6: 23. 1846.** Sec. Kuzmina & Nersesyan (2012)

***Dianthuscampestris* M.Bieb.﻿, Fl. Taur.-Caucas. 1: 326. 1808.** Sec. Marhold (2011)

= *Dianthushypanicus* Besser ex Rchb.﻿, Fl. Germ. Excurs.: 809. 1832 syn. sec. WFO 2018

= *Dianthuspseudoversicolor* Klokov﻿, Fl. RSS Ucr. 4: 660. 1952 syn. sec. WFO 2018

= Dianthuscampestrissubsp.arenarius Širj.﻿ syn. sec. WFO 2018

= Dianthuscampestrissubsp.serbanii Prodán﻿ syn. sec. Marhold (2011) ≡ *Dianthusserbanii* (Prodán) Prodán﻿, Fl. Reipubl. Popul. Roman. 2: 670. 1953 syn. sec. Marhold (2011)

**Dianthuscampestrissubsp.campestris**﻿

**Dianthuscampestrissubsp.laevigatus (Gruner) Klokov﻿, Fl. RSS Ucr. 4: 625. 1952.** Sec. Marhold (2011)

≡ Dianthuscampestrisvar.laevigatus Gruner﻿ in Bull. Soc. Imp. Naturalistes Moscou 41(2): 124. 1868 syn. sec. Marhold (2011) ≡ *Dianthuslaevigatus* (Gruner) Klokov﻿ in Novosti Sist. Vyssh. Nizsh. Rast. 1980: 99. 1980 syn. sec. Marhold (2011)

**Dianthuscampestrissubsp.steppaceus Širj.﻿ in Širj. & Lavrenko, Consp. Crit. Fl. Prov. Charkov.: 1. 1926.** Sec. Marhold (2011)

***Dianthuscandicus* (P.W.Ball & Heywood) Madhani & Heubl﻿ in Taxon 67(1): 103. 2018.** Sec. Madhani et al. (2018)

≡ *Petrorhagiacandica* P.W.Ball & Heywood﻿ in Bull. Brit. Mus. (Nat. Hist.), Bot. 3: 141. 1964 syn. sec. Kew WCVP (2019); ≡ *Fiedleriacandica* (P.W.Ball & Heywood) Ovcz.﻿, Fl. Tadzhikskoi S.S.R. 3: 608. 1968 syn. sec. Kew WCVP (2019).

***Dianthuscanescens* K.Koch﻿ in Linnaea 15: 710. 1841.** Sec. Kuzmina & Nersesyan (2012)

≡ Dianthusfimbriatusvar.canescens (K.Koch) Boiss.﻿, Fl. Orient. 1: 496. 1867 syn. sec. Kuzmina & Nersesyan (2012)

***Dianthuscapitatus* Balb. ex DC.﻿, Cat. Pl. Horti Monsp.: 103. 1813.** Sec. Kuzmina & Nersesyan (2012)

***Dianthuscapitatus* J.St.-Hil.﻿, Pl. France 3: 70. 1809.** Sec. Czerepanov (1995)

= *Dianthuscephalotes* Ser.﻿, Prodr. 1: 356. 1824 syn. sec. Czerepanov (1995)

= *Dianthusglaucophyllus* Boiss.﻿, Fl. Orient. 1: 514. 1867 syn. sec. Czerepanov (1995)

= *Dianthuscapillatus* Meinsh.﻿, Beitr. Pfl. Geogr. Sued-Ural-Geb.: 6 syn. sec. Czerepanov (1995)

***Dianthuscapitellatus* Klokov﻿, Fl. RSS Ucr. 4: 659. 1952.** Sec. Kuzmina & Nersesyan (2012)

≡ Dianthusborbasiisubsp.capitellatus (Klokov) Tutin﻿ in Feddes Repert. Spec. Nov. Regni Veg. 68: 192. 1963 syn. sec. Kuzmina & Nersesyan (2012)

= *Dianthuspseudomembranaceus* Schischk. ex Grossh.﻿, Fl. Cauc. 2(3): 282. 1945 syn. sec. Kuzmina & Nersesyan (2012)

***Dianthuscarbonatus* Klokov﻿ in Sc. Mag. Biol. 1927: 15. 1927.** Sec. Czerepanov (1995)

***Dianthuscarmelitarum* Reut. ex Boiss.﻿, Fl. Orient. 1: 512. 1867.** Sec. Kuzmina & Nersesyan (2012)

= *Dianthusartwinensis* Schischk. ex Grossh., Fl. Cauc. 2(3): 284. 1945 syn. sec. Kuzmina & Nersesyan (2012)

***Dianthuscarthusianorum* L.﻿, Sp. Pl.: 409. 1753.** Sec. Marhold (2011)

≡ *Tunicacarthusianorum* (L.) Scop.﻿, Fl. Carniol., ed. 2, 1: 299. 1771 syn. sec. POWO. Plants of the World Online. Facilitated by the Royal Botanic Gardens, Kew. ≡ *Caryophylluscarthusianorum* (L.) Moench﻿, Methodus: 59. 1794 syn. sec. POWO. Plants of the World Online. Facilitated by the Royal Botanic Gardens, Kew. ≡ *Silenecarthusianorum* (L.) E.H.L.Krause﻿, Deutschl. Fl. Abbild., ed. 2, 5: 110. 1901 syn. sec. POWO. Plants of the World Online. Facilitated by the Royal Botanic Gardens, Kew.;

= *Dianthusatrorubens* All.﻿, Fl. Pedem. 2: 75. 1785 syn. sec. Marhold (2011) ≡ *Caryophyllusatrorubens* (All.) Moench﻿, Suppl. Meth.: 23. 1802 syn. sec. POWO. Plants of the World Online. Facilitated by the Royal Botanic Gardens, Kew. ≡ Dianthuscarthusianorumvar.atrorubens (All.) Ser.﻿, Prodr. 1: 357. 1824 syn. sec. this publication ≡ Dianthuscarthusianorumsubsp.atrorubens (All.) Hegi﻿ in Allg. Bot. Z. Syst. 17: 15. 1911 syn. sec. Marhold (2011)

= *Dianthusvaginatus* Chaix﻿, Hist. Pl. Dauphiné 1: 330. 1786 syn. sec. WFO 2018 ≡ *Cylichnanthusvaginatus* (Chaix) Dulac﻿, Fl. Hautes-Pyrénées: 261. 1867 syn. sec. Kew WCVP (2019)

= *Dianthusmontanus* F.W.Schmidt﻿ in Neuere Abh. Königl. Böhm. Ges. Wiss. 1: 30. 1791 syn. sec. WFO 2018

= *Dianthusclavatus* Spreng.﻿, Neue Entdeck. Pflanzenk. 2: 169. 1821 syn. sec. WFO 2018

= Dianthuscarthusianorumvar.anisopodus Ser.﻿, Prodr. 1: 357. 1824 syn. sec. Kew WCVP (2019)

= Dianthuscarthusianorumvar.nanus Ser.﻿, Prodr. 1: 357. 1824 syn. sec. Kew WCVP (2019)

= *Dianthusnanus* Sweet﻿, Hort. Brit.: 41. 1826 syn. sec. WFO 2018

= Dianthusatrorubensvar.intermedius Gaudin﻿, Fl. Helv. 3: 146. 1828 syn. sec. Kew WCVP (2019)

= Dianthuscarthusianorumvar.pygmaeus Gaudin﻿, Fl. Helv. 3: 145. 1828 syn. sec. Kew WCVP (2019)

= *Dianthusallionii* Colla﻿, Herb. Pedem. 1: 297. 1833 syn. sec. WFO 2018

= *Dianthussanguineus* Vis.﻿, Index Seminum (PAD) 1845: 1. 1845 syn. sec. Marhold (2011) ≡ Dianthusatrorubensvar.sanguineus (Vis.) Arcang.﻿, Comp. Fl. Ital.: 84. 1882 syn. sec. this publication ≡ Dianthuscarthusianorumvar.sanguineus (Vis.) Tanfani﻿, Fl. Ital. 9: 254. 1892 syn. sec. this publication ≡ Dianthuscarthusianorumsubsp.sanguineus (Vis.) Hegi﻿ in Ill. Fl. Mitt. Eur. 3: 323. 1910 syn. sec. Marhold (2011)

= *Dianthusferrugineus* Pourr. ex Gren. & Godr.﻿, Fl. France 1: 232. 1847 syn. sec. WFO 2018

= *Dianthusgramineus* Schur﻿ in Verh. Mitth. Siebenbürg. Vereins Naturwiss. Hermannstadt 4: 11. 1853 syn. sec. WFO 2018

= *Dianthuscongestus* Boreau﻿, Fl. Centre France ed. 3, 2: 90. 1857 syn. sec. WFO 2018

= Dianthuscarthusianorumvar.campestris Heuff.﻿ in Verh. Zool.-Bot. Ges. Wien 8: 68. 1858 syn. sec. Kew WCVP (2019)

= Dianthuscarthusianorumvar.ternatus Heuff.﻿ in Verh. Zool.-Bot. Ges. Wien 8: 68. 1858 syn. sec. Kew WCVP (2019)

= *Dianthusgraminifolius* Schur﻿ in Verh. Mitth. Siebenbürg. Vereins Naturwiss. Hermannstadt 10: 144. 1859 syn. sec. WFO 2018

= *Dianthustenuifolius* Schur﻿ in Verh. Mitth. Siebenbürg. Vereins Naturwiss. Hermannstadt 10: 143. 1859 syn. sec. WFO 2018 ≡ Dianthuscarthusianorumsubsp.tenuifolius (Schur) Hegi﻿ syn. sec. Marhold (2011)

= Dianthuscarthusianorumvar.saxigenus Schur﻿, Enum. Pl. Transsilv.: 93. 1866 syn. sec. WFO 2018 ≡ Dianthuscarthusianorumsubsp.saxigenus (Schur) Dostál﻿ in Folia Mus. Rerum Nat. Bohemiae Occid., Bot. 21: 5. 1984 syn. sec. WFO 2018

= *Dianthuschloaephyllus* Schur﻿, Enum. Pl. Transsilv.: 95. 1866 syn. sec. WFO 2018

= *Dianthusrupicola* Schur﻿, Enum. Pl. Transsilv.: 93. 1866 syn. sec. Schur (1866)

= *Dianthusrupicolus* Schur﻿, Enum. Pl. Transsilv.: 93. 1866 syn. sec. WFO 2018

= *Dianthussubneglectus* Schur﻿, Enum. Pl. Transsilv.: 95. 1866 syn. sec. WFO 2018

= Dianthuscarthusianorumvar.puberulus Simonk.﻿, Mat. Term. Közlem. 15: 531. 1878 syn. sec. Marhold (2011) ≡ Dianthuscarthusianorumsubsp.puberulus (Simonk.) Soó﻿ in Feddes Repert. 83: 161. 1972 syn. sec. Marhold (2011) ≡ *Dianthuspuberulus* (Simonk.) A.Kern.﻿ syn. sec. Marhold (2011)

= *Dianthusatropurpureus* Gromov ex Trautv.﻿ in Trudy Imp. S.-Peterburgsk. Bot. Sada 8: 130. 1883 syn. sec. WFO 2018

= *Dianthuscarpathicus* Woł.﻿, Spraw. Komis. Fizjogr. 22(2): 214. 1888 syn. sec. WFO 2018

= *Dianthussemperflorens* Voss﻿ in Gartenflora 44: 514. 1895 syn. sec. WFO 2018

= Dianthuscarthusianorumvar.brachyanthus Dörfl. & Hayek﻿ in Oesterr. Bot. Z. 70: 12. 1921 syn. sec. Kew WCVP (2019)

= Dianthuscarthusianorumvar.longisquamis Kulcz.﻿, Fl. Polska 2: 159. 1921 syn. sec. WFO 2018

= *Dianthusceretanicus* Sennen﻿ in Bol. Soc. Ibér. Ci. Nat. 25: 147. 1926 syn. sec. WFO 2018

= Dianthuscarthusianorumvar.tenorei Lacaita﻿ in Nuovo Giorn. Bot. Ital. n.s., 34: 188. 1927 syn. sec. WFO 2018 ≡ Dianthuscarthusianorumsubsp.tenorei (Lacaita) Pignatti﻿ in Giorn. Bot. Ital. 111: 46. 1977 syn. sec. Marhold (2011)

= *Dianthusmontivagus* Domin﻿ in Acta Bot. Bohem. 8: 53. 1929 syn. sec. WFO 2018

= *Dianthusvelebiticus* Borbás ex Degen﻿, Fl. Veleb. 2: 97. 1937 syn. sec. WFO 2018

= Dianthuscarthusianorumvar.capillifrons Borbás﻿ syn. sec. Marhold (2011) ≡ *Dianthuscapillifrons* (Borbás) H.Neumayer﻿ in Oesterr. Bot. Z. 91: 236. 1942 syn. sec. WFO 2018 ≡ Dianthuscarthusianorumsubsp.capillifrons (Borbás) Soó﻿ in Acta Bot. Acad. Sci. Hung. 23: 391. 1977 [“1978”] syn. sec. Marhold (2011)

= Dianthuscarthusianorumvar.parviflorus Čelak.﻿ syn. sec. WFO 2018 ≡ Dianthuscarthusianorumsubsp.parviflorus (Čelak.) Dostál﻿ in Folia Mus. Rerum Nat. Bohemiae Occid., Bot. 21: 5. 1984 syn. sec. WFO 2018

= Dianthussanguineusvar.atrisquamatus Novák﻿ syn. sec. WFO 2018 ≡ Dianthuscarthusianorumf.atrisquamatus (Novák) Gajić﻿, Fl. SR Srbije 9: 64. 1977 syn. sec. WFO 2018

– *Dianthusfasciculatus* Gilib.﻿, Fl. Lit. Inch. 2: 161. 1782, nom. inval. syn. sec. WFO 2018

**Dianthuscarthusianorumsubsp.carthusianorum**﻿

**Dianthuscarthusianorumsubsp.latifolius (Griseb. & Schenk) Hegi﻿ in Allg. Bot. Z. Syst. 17: 13. 1911.** Sec. Marhold (2011)

≡ Dianthuscarthusianorumvar.latifolius Griseb. & Schenk﻿ in Arch. Naturgesch. 18: 300. 1852 syn. sec. WFO 2018

= Dianthusmontivagusvar.carsticus Novák﻿ syn. sec. Kew WCVP (2019) ≡ Dianthuscarthusianorumsubsp.carsticus (Novák) Dostál﻿ in Folia Mus. Rerum Nat. Bohemiae Occid., Bot. 21: 5. 1984 syn. sec. Kew WCVP (2019)

**Dianthuscarthusianorumsubsp.polonicus (Zapał.) Kovanda﻿ in Preslia 56: 297. 1984.** Sec. WFO 2018

≡ *Dianthuspolonicus* Zapał.﻿, Consp. Fl. Gallic. Crit. 3: 122. 1911 syn. sec. WFO 2018 ≡ Dianthuscarthusianorumvar.polonicus (Zapał.) Kulcz.﻿, Fl. Polska 2: 159. 1921 syn. sec. WFO 2018

**Dianthuscarthusianorumsubsp.sudeticus Kovanda﻿ in Preslia 52: 122. 1980.** Sec. Marhold (2011)

***Dianthuscaryophyllus* L.﻿, Sp. Pl.: 410. 1753.** Sec. Marhold (2011)

≡ *Silenecaryophylla* (L.) E.H.L.Krause﻿, Deutschl. Fl. Abbild., ed. 2, 5: 111. 1901 syn. sec. POWO. Plants of the World Online. Facilitated by the Royal Botanic Gardens, Kew.; ≡ Dianthuscaryophyllusvar.coronarius L.﻿, Sp. Pl. 1: 410. 1753 syn. sec. Domina et al. (2021) ≡ Dianthuscaryophyllussubsp.coronarius (L.) Maire﻿, Fl. Afrique N. 10: 318. 1963 syn. sec. this publication ≡ *Tunicacaryophyllus* (L.) Scop.﻿, Fl. Carniol., ed. 2, 1: 301. 1771 syn. sec. POWO. Plants of the World Online. Facilitated by the Royal Botanic Gardens, Kew.; – *Dianthuscoronarius* (L.) Burm.f.﻿, Fl. Ind. Prodr. Fl. Cap.: 13. 1768, nom. illeg. syn. sec. Domina et al. (2021) – *Dianthuscoronarius* Lam.﻿, Fl. Franç. 2: 536. 1779, nom. illeg. syn. sec. Domina et al. (2021)

= *Caryophyllustunica* Garsault﻿, Fig. Pl. Méd.: t. 203. 1764 syn. sec. POWO. Plants of the World Online. Facilitated by the Royal Botanic Gardens, Kew.

= *Dianthusmoschatus* J.F.Gmel.﻿, Syst. Nat.: 711. 1791 syn. sec. Kuzmina & Nersesyan (2012)

= Dianthuscaryophyllusvar.minor Gray﻿, Nat. Arr. Brit. Pl. 2: 644. 1822 [“1821”] syn. sec. Kew WCVP (2019)

= Dianthuscaryophyllusvar.carduinus Ser.﻿, Prodr. 1: 359. 1824 syn. sec. Kew WCVP (2019)

= *Dianthuscorsicus* Link ex Spreng.﻿, Syst. Veg., ed. 16, 2: 376. 1825 syn. sec. Kuzmina & Nersesyan (2012)

= *Dianthusarbuscula* Lindl.﻿ in Bot. Reg. 13: t. 1086. 1827 syn. sec. Kuzmina & Nersesyan (2012)

= *Dianthusarrectus* Dumort.﻿, Fl. Belg.: 196. 1827 syn. sec. Kuzmina & Nersesyan (2012)

= Dianthuscaryophyllusvar.wakefieldii C.Morren﻿, Hort. Belge 2: 7. 1834 syn. sec. Kew WCVP (2019)

= *Dianthusmultinervis* Vis.﻿, Fl. Dalmat. 3: 164. 1850 syn. sec. Kuzmina & Nersesyan (2012)

= *Dianthuslongicaulis* Costa﻿, Introd. Fl. Cataluña: 36. 1864 syn. sec. Kuzmina & Nersesyan (2012)

= *Dianthusacinifolius* Schur﻿, Enum. Pl. Transsilv.: 97. 1866 syn. sec. Kuzmina & Nersesyan (2012)

= *Dianthusbinatus* Schur﻿, Enum. Pl. Transsilv.: 97. 1866 syn. sec. Kuzmina & Nersesyan (2012)

= *Dianthuskayserianus* Schur﻿, Enum. Pl. Transsilv.: 97. 1866 syn. sec. Kuzmina & Nersesyan (2012)

= *Dianthusminiatus* A.Huet ex Nyman﻿, Consp. Fl. Eur. 1: 105. 1878 syn. sec. Kuzmina & Nersesyan (2012)

= Dianthuscaryophyllusf.intermedius Pamp.﻿ in Boll. Mus. Republ. San Marino 1: 142. 1917 syn. sec. Kew WCVP (2019)

***Dianthuscaucaseus* Sims﻿ in Bot. Mag. 21: t. 795. 1804.** Sec. Kuzmina & Nersesyan (2012)

= *Dianthusdiscolor* Sims in Bot. Mag. 29: t. 1162. 1808 syn. sec. Kuzmina & Nersesyan (2012)

***Dianthuscharidemi* Pau﻿ in Bull. Acad. Int. Geogr. Bot. 16(206): 74. 1906.** Sec. Bernal et al. (1990)

≡ Dianthuscintranussubsp.charidemi (Pau) Tutin﻿ in Feddes Repert. Spec. Nov. Regni Veg. 68: 190. 1963 syn. sec. Bernal et al. (1990)

***Dianthuschimanimaniensis* S.S.Hooper﻿ in Kew Bull. 13: 318. 1958.** Sec. African Plant Database (version 3.4.0)

***Dianthuschinensis* L.﻿, Sp. Pl.: 411. 1753.** Sec. Dequan & Turland (2001)

= *Dianthussequieri* Chaix﻿, Vill. Fl. Dauph. 1: 330. 1786 syn. sec. Dequan & Turland (2001)

= *Dianthuspulcher* Salisb.﻿, Prodr. Stirp. Chap. Allerton: 303. 1796 syn. sec. Dequan & Turland (2001)

= *Dianthusscaber* Schleich. ex Suter﻿, Helvet. Fl. 1: 259. 1802 syn. sec. Dequan & Turland (2001) ≡ Dianthusserratusvar.scaber (Schleich. ex Suter) DC.﻿, Fl. Franç., ed. 3, 5: 601. 1815 syn. sec. this publication ≡ Dianthuscollinusvar.scaber (Schleich. ex Suter) Gaudin﻿, Fl. Helv. 3: 147. 1828 syn. sec. this publication

= *Dianthustataricus* Fisch.﻿, Cat. Jard. Gorenki ed. 2: 59. 1812 syn. sec. Dequan & Turland (2001)

= *Dianthusumbellatus* DC.﻿, Cat. Pl. Horti Monsp.: 104. 1813 syn. sec. Dequan & Turland (2001)

= *Dianthusibericus* Willd.﻿, Enum. Pl., Suppl.: 24. 1814 syn. sec. Dequan & Turland (2001)

= *Dianthuspatens* Willd.﻿, Enum. Pl., Suppl.: 24. 1814 syn. sec. Dequan & Turland (2001)

= *Dianthusfischeri* Spreng.﻿, Pl. Min. Cogn. Pug. 2: 62. 1815 syn. sec. Dequan & Turland (2001)

= *Dianthusruthenicus* Roem. ex Poir.﻿, Encycl. Suppl. 4: 131. 1816 syn. sec. Dequan & Turland (2001)

= *Dianthusochroleucus* Link﻿, Enum. Hort. Berol. Alt. 1: 420. 1821 syn. sec. Dequan & Turland (2001)

= *Dianthusversicolor* Fisch. ex Link﻿, Enum. Hort. Berol. Alt. 1: 420. 1821 syn. sec. Dequan & Turland (2001) ≡ Dianthuschinensisvar.versicolor (Fisch. ex Link) Y.C.Ma﻿, Fl. Intramongolica 2: 191. 1978 [“1979”] syn. sec. Dequan & Turland (2001) ≡ Dianthuschinensissubsp.versicolor (Fisch. ex Link) Vorosch.﻿, Florist. Issl. Razn. Rayonakh SSSR: 167. 1985 syn. sec. Dequan & Turland (2001)

= *Dianthuswilldenowii* Link﻿, Enum. Hort. Berol. Alt. 1: 420. 1821 syn. sec. Dequan & Turland (2001)

= *Dianthusschraderi* Rchb.﻿ in Iconogr. Bot. Exot. 1: 24. 1824 syn. sec. Dequan & Turland (2001)

= *Dianthusdentosus* Fisch. ex Rchb.﻿ in Iconogr. Bot. Pl. Crit. 6: 32. 1828 syn. sec. Dequan & Turland (2001) ≡ Dianthuschinensisvar.dentosus (Fisch. ex Rchb.) Debeaux﻿ in Act. Simpo. Internac. Bot. Pius Font Quer 31: 124. 1876 syn. sec. Dequan & Turland (2001) ≡ Dianthusseguierivar.dentosus (Fisch. ex Rchb.) Franch.﻿, Pl. David. 1: 46. 1884 syn. sec. Dequan & Turland (2001) ≡ Dianthussequierivar.dentosus (Fisch. ex Rchb.) Franch.﻿, Pl. David. 1: 46. 1884 syn. sec. Dequan & Turland (2001)

= *Dianthussinensis* Link﻿, Handbuch 2: 237. 1831 syn. sec. Dequan & Turland (2001)

= Dianthuschinensisvar.sylvaticus W.D.J.Koch﻿, Syn. Fl. Germ. Helv.: 96. 1835 syn. sec. Dequan & Turland (2001)

= *Dianthusaltaicus* Willd. ex Ledeb.﻿, Fl. Ross. 1: 278. 1842 syn. sec. Dequan & Turland (2001)

= *Dianthusjeniseensis* Less. ex Ledeb.﻿, Fl. Ross. 1: 278. 1842 syn. sec. Dequan & Turland (2001)

= Dianthuschinensisvar.macrosepalus Franch. ex L.H.Bailey﻿, Cycl. Amer. Hort.: 100. 1900 syn. sec. Dequan & Turland (2001)

= *Dianthuslaciniatus* Makino﻿ in Bot. Mag. (Tokyo) 17: 60. 1903 syn. sec. Dequan & Turland (2001)

= *Dianthusmorii* Nakai in Bot. Mag. (Tokyo) 28: 302. 1914 syn. sec. Dequan & Turland (2001) ≡ Dianthuschinensisvar.morii (Nakai) Y.C.Chu﻿, Fl. Plant. Herb. Chinae bor.-or. 3: 49. 1975 syn. sec. Dequan & Turland (2001)

= *Dianthuspineticola* Kleopow﻿ in Izv. Kievsk. Bot. Sada 12–13: 161. 1931 syn. sec. Dequan & Turland (2001)

= Dianthusversicolorvar.alpinus Krylov﻿, Fl. Zapadnoi Sibiri 5: 1104. 1931 syn. sec. Kew WCVP (2019)

= Dianthuschinensisvar.longisquama Nakai & Kitag.﻿ in Rep. Exped. Manchoukou Sect. IV, 1: 23. 1934 syn. sec. Dequan & Turland (2001)

= Dianthuschinensisvar.ignescens Nakai﻿ in Rep. Exped. Manchoukou Sect. IV, 4(2): 15. 1935 syn. sec. Dequan & Turland (2001) ≡ Dianthuschinensisf.ignescens (Nakai) Kitag.﻿ in J. Jap. Bot. 34: 5. 1959 syn. sec. Dequan & Turland (2001)

= *Dianthussubulifolius* Kitag.﻿ in Rep. Exped. Manchoukou Sect. IV, 2: 16. 1935 syn. sec. Dequan & Turland (2001) ≡ Dianthusversicolorvar.subulifolius (Kitag.) Y.C.Chu﻿, Fl. Plant. Herb. Chinae bor.-or. 3: 50. 1975 syn. sec. Dequan & Turland (2001) ≡ Dianthuschinensisvar.subulifolius (Kitag.) Y.C.Ma﻿, Fl. Intramongolica 2: 191. 1978 [“1979”] syn. sec. Dequan & Turland (2001)

= Dianthussubulifoliusf.leucopetalus Kitag.﻿ in Rep. Exped. Manchoukou Sect. IV, 2: 17. 1935 syn. sec. Dequan & Turland (2001) ≡ Dianthusversicolorf.leucopetalus (Kitag.) Y.C.Chu﻿, Fl. Plant. Herb. Chinae bor.-or. 3: 50. 1975 syn. sec. Dequan & Turland (2001)

= Dianthuschinensisvar.liaotungensis Y.C.Chu﻿, Fl. Plant. Herb. Chinae bor.-or. 3: 227. 1975 syn. sec. Dequan & Turland (2001)

= Dianthuschinensissubsp.paracampestris Vorosch.﻿ in Byull. Moskovsk. Obshch. Isp. Prir., Otd. Biol. n.s., 83(5): 116. 1978 syn. sec. Dequan & Turland (2001)

= Dianthusversicolorvar.ninelli Peschkova﻿, Fl. Tsentralnoi Sibiri 1: 334. 1979 syn. sec. Dequan & Turland (2001) ≡ Dianthusversicolorsubsp.ninelli (Peschkova) Baikov & N.V.Vlassova﻿, Rast. Mir Aziatsk. Rossii 3(23): 21. 2016 syn. sec. Kew WCVP (2019)

= Dianthuschinensisvar.trinervisD.Q.Lu﻿ in Bull. Bot. Res., Harbin 15: 185. 1995 syn. sec. Dequan & Turland (2001)

= Dianthuschinensisvar.jingpoensis G.Y.Zhang & X.Y.Yuan﻿ in Bull. Bot. Res., Harbin 18: 10. 1998 syn. sec. Dequan & Turland (2001)

= Dianthuschinensisvar.shandongensis J.X.Li & F.Q.Zhou﻿ in Bull. Bot. Res., Harbin 21: 511. 2001 syn. sec. Dequan & Turland (2001)

= Dianthuschinensisf.albiflora Y.N.Lee﻿ in Bull. Korea Pl. Res. 4: 28. 2004 syn. sec. Dequan & Turland (2001)

= Dianthuschinensisvar.serpens Y.N.Lee﻿ in Bull. Korea Pl. Res. 4: 29. 2004 syn. sec. Dequan & Turland (2001)

***Dianthuschouardii* Dobignard﻿ in J. Bot. Soc. Bot. France 48: 15. 2009.** Sec. African Plant Database (version 3.4.0)

***Dianthuscibrarius* Clementi﻿ in Mem. Reale Accad. Sci. Torino II 16: 20. 1855.** Sec. Kuzmina & Nersesyan (2012)

***Dianthusciliatus* Guss.﻿, Ind. Sem. Horto Boccad. 1825: 5. 1825.** Sec. Marhold (2011)

= *Dianthuslitoralis* Host﻿, Fl. Austriaca 1: 522. 1827 syn. sec. WFO 2018

**Dianthusciliatussubsp.ciliatus**﻿

**Dianthusciliatussubsp.dalmaticus (Čelak.) Hayek﻿ in Repert. Spec. Nov. Regni Veg. Beih. 30(1): 246. 1924.** Sec. Marhold (2011)

≡ *Dianthusdalmaticus* Čelak.﻿ in Oesterr. Bot. Z. 35: 189. 1885 syn. sec. Marhold (2011)

= *Dianthusracemosus* Vis.﻿ in Flora 12(1 Erg.): 12. 1829 syn. sec. Marhold (2011) ≡ Dianthusciliatussubsp.racemosus (Vis.) Hayek﻿ in Repert. Spec. Nov. Regni Veg. Beih. 30(1): 246. 1924 syn. sec. Marhold (2011)

**Dianthusciliatussubsp.medunensis (Beck & Szyszyl.) Trinajstić﻿ in Suppl. Fl. Anal. Jugosl. 6: 8. 1979.** Sec. Marhold (2011)

≡ *Dianthusmedunensis* Beck & Szyszył.﻿, Pl. Cernagor. Lect.: 65. 1888 syn. sec. WFO 2018 – ﻿Dianthusciliatussubsp.medunensis (Beck & Szyszyl.) Trinajstić in Suppl. Fl. Anal. Jugosl. 5: 751. 1979, nom. inval. syn. sec. this publication

**Dianthus×cincinnatus Lem.﻿ in Ill. Hort. 11: t. 388. 1864.** Sec. POWO. Plants of the World Online. Facilitated by the Royal Botanic Gardens, Kew.

= Dianthus×isensis Hirahata & Kitam.﻿ in Acta Phytotax. Geobot. 20: 205. 1962 syn. sec. POWO. Plants of the World Online. Facilitated by the Royal Botanic Gardens, Kew. ≡ Dianthus×cincinnatusf.isensis (Hirahata & Kitam.) M.Hiroe﻿, Pl. Bashos & Busons Hokku Lit. 8: 489. 1971 syn. sec. POWO. Plants of the World Online. Facilitated by the Royal Botanic Gardens, Kew.

= Dianthus×cincinnatusf.longipetalus M.Hiroe﻿, Pl. Bashos & Busons Hokku Lit. 8: 489. 1971 syn. sec. POWO. Plants of the World Online. Facilitated by the Royal Botanic Gardens, Kew.

= Dianthus×cincinnatusf.rosalbus M.Hiroe﻿, Pl. Bashos & Busons Hokku Lit. 8: 489. 1971 syn. sec. POWO. Plants of the World Online. Facilitated by the Royal Botanic Gardens, Kew.

***Dianthuscinnamomeus* Sm.﻿, Fl. Graec. Prodr. 1(2): 287. 1809.** Sec. Dimopoulos et al. (2013)

**Dianthuscinnamomeussubsp.cinnamomeus**﻿

**Dianthuscinnamomeussubsp.naxensis Runemark ex Strid﻿ in Strid & Tan, Fl. Hellenica 1: 352. 1997.** Sec. Dimopoulos et al. (2013)

***Dianthuscintranus* Boiss. & Reut.﻿, Pugill. Pl. Afr. Bor. Hispan.: 20. 1852.** Sec. Bernal et al. (1990)

**Dianthuscintranussubsp.atrosanguineus (Emb. & Maire) Greuter & Burdet﻿ in Willdenowia 12: 186. 1982.** Sec. Marhold (2011)

≡ Dianthusgaditanussubsp.atrosanguineus Emb. & Maire﻿, Mém. Soc. Sci. Nat. Maroc 21–22: 26. 1930 syn. sec. Marhold (2011) ≡ Dianthusanticariussubsp.atrosanguineus (Emb. & Maire) Valdés & Mateos﻿ in Lagascalia 29: 157. 2009 syn. sec. WFO 2018

**Dianthuscintranussubsp.barbatus R.Fern. & Franco﻿ in Franco, Nova Fl. Portugal 1: 159. 1971.** Sec. Bernal et al. (1990)

**Dianthuscintranussubsp.byzacenus (Burollet) Greuter & Burdet﻿ in Willdenowia 12: 186. 1982.** Sec. Marhold (2011)

≡ *Dianthusbyzacenus* Burollet﻿, Sahel Sousse: 35. 1927 syn. sec. Marhold (2011) ≡ Dianthusgaditanussubsp.byzacenus (Burollet) Maire﻿, Fl. Afrique N. 10: 307. 1963 syn. sec. Kew WCVP (2019)

**Dianthuscintranussubsp.cintranus**﻿

**Dianthuscintranussubsp.jahandiezii (Maire) Greuter & Burdet﻿ in Willdenowia 12: 186. 1982.** Sec. Marhold (2011)

≡ Dianthusgaditanussubsp.jahandiezii Maire﻿, Mém. Soc. Sci. Nat. Maroc 15: 17. 1927 syn. sec. WFO 2018 ≡ Dianthusanticariussubsp.jahandiezii (Maire) Valdés & Mateos﻿ in Lagascalia 29: 157. 2009 syn. sec. WFO 2018

**Dianthuscintranussubsp.maroccanus (F.N.Williams) Greuter & Burdet﻿ in Willdenowia 12: 186. 1982.** Sec. Marhold (2011)

≡ Dianthusgaditanusvar.maroccanus F.N.Williams﻿ in J. Linn. Soc., Bot. 29: 423. 1893 syn. sec. Marhold (2011) ≡ Dianthusgaditanussubsp.maroccanus (F.N.Williams) Maire﻿, Mém. Soc. Sci. Nat. Maroc 15: 17. 1927 syn. sec. Kew WCVP (2019) ≡ Dianthusanticariussubsp.maroccanus (F.N.Williams) Valdés & Mateos﻿ in Lagascalia 29: 157. 2009 syn. sec. WFO 2018

= Dianthusgaditanusvar.rifeus Pau & Sennen﻿, Cat. Fl. Rif Orient.: 19. 1933 syn. sec. Kew WCVP (2019)

**Dianthuscintranussubsp.mauritanicus (Pomel) Greuter & Burdet﻿ in Willdenowia 12: 186. 1982.** Sec. Marhold (2011)

≡ *Dianthusmauritanicus* Pomel﻿, Nouv. Mat. Fl. Atl. 1: 333. 1874 syn. sec. Marhold (2011) ≡ Dianthusserrulatussubsp.mauritanicus (Pomel) Batt.﻿, Fl. Algérie, Dicot.: 145. 1888 syn. sec. Kew WCVP (2019) ≡ Dianthusgaditanussubsp.mauritanicus (Pomel) Maire﻿, Mém. Soc. Sci. Nat. Maroc 21–22: 26. 1930 syn. sec. Kew WCVP (2019) ≡ Dianthusanticariussubsp.mauritanicus (Pomel) Valdés & Mateos﻿ in Lagascalia 29: 157. 2009 syn. sec. WFO 2018

**Dianthuscintranussubsp.mentagensis (Maire) Greuter & Burdet﻿ in Willdenowia 12: 186. 1982.** Sec. Marhold (2011)

≡ Dianthusgaditanussubsp.mentagensis Maire﻿ in Bull. Soc. His. Nat. Afrique N. 23: 168. 1932 syn. sec. Marhold (2011) ≡ Dianthusanticariussubsp.mentagensis (Maire) Valdés & Mateos﻿ in Lagascalia 29: 157. 2009 syn. sec. WFO 2018

**Dianthuscintranussubsp.occidentalis (Quézel) Mathez﻿ in Willdenowia 13: 280. 1983 [“1984”].** Sec. Marhold (2011)

≡ Dianthusgaditanussubsp.occidentalis Quézel﻿ in Bull. Soc. His. Nat. Afrique N. 44: 197. 1953 syn. sec. Marhold (2011) ≡ Dianthusanticariussubsp.occidentalis (Quézel) Valdés & Mateos﻿ in Lagascalia 29: 157. 2009 syn. sec. WFO 2018

***Dianthuscollinus* Waldst. & Kit.﻿ in Descr. Icon. Pl. Hung. 1: 51. 1800.** Sec. Czerepanov (1995)

≡ Dianthuscarthusianorumsubsp.collinus (Waldst. & Kit.) Pers.﻿, Syn. Pl. 1: 493. 1805 syn. sec. Kew WCVP (2019) ≡ Dianthusaspervar.collinus (Waldst. & Kit.) Ser.﻿, Prodr. 1: 357. 1824 syn. sec. Kew WCVP (2019) ≡ Dianthusseguierivar.collinus (Waldst. & Kit.) W.D.J.Koch﻿, Syn. Fl. Germ. Helv. 1: 96. 1835 syn. sec. Kew WCVP (2019) ≡ Dianthusseguierisubsp.collinus (Waldst. & Kit.) Arcang.﻿, Comp. Fl. Ital.: 84. 1882 syn. sec. Kew WCVP (2019)

= Dianthuscollinusvar.minor Gaudin﻿, Fl. Helv. 3: 147. 1828 syn. sec. Kew WCVP (2019)

= Dianthusseguierisubsp.scaber Čelak.﻿, Prodr. Fl. Böhmen: 507. 1875 syn. sec. Czerepanov (1995)

***Dianthuscommutatus* (Zapał.) Klokov﻿, Fl. RSS Ucr. 4: 607. 1952.** Sec. Czerepanov (1995)

≡ Dianthuscarthusianorumvar.commutatus Zapał.﻿, Fl. Galic. Crit.: 119. 1911 syn. sec. Czerepanov (1995)

***Dianthuscorymbosus* Sm.﻿, Fl. Graec. Prodr. 1(2): 285. 1809.** Sec. Dimopoulos et al. (2013)

= *Dianthuscorymbosus* Poir.﻿, Encycl. Suppl. 4: 124. 1816 syn. sec. WFO 2018

= *Dianthuspoiretianus* Ser.﻿, Prodr. 1: 360. 1824 syn. sec. WFO 2018

= *Dianthuschalcidicus* Halácsy﻿ in Verh. K. K. Zool.-Bot. Ges. Wien 48: 707. 1898 syn. sec. WFO 2018

***Dianthuscostae* Willk.﻿, Prodr. Fl. Hispan. 3: 683. 1878.** Sec. Bernal et al. (1990)

≡ Dianthuspyrenaicussubsp.costae (Willk.) O.Bolòs & Vigo﻿ in Butl. Inst. Catalana Hist. Nat., Secc. Bot. 38(1): 187. 1974 syn. sec. Bernal et al. (1990) ≡ Dianthusalgetanussubsp.costae (Willk.) Romo﻿, Fl. Veg. Montsec: 98. 1989, nom. illeg. syn. sec. Kew WCVP (2019)

= *Dianthusciliatus* Costa﻿, Introd. Fl. Cataluña: 36. 1864 syn. sec. Bernal et al. (1990)

= *Dianthusmarianii* Sennen﻿, Diagn. Nouv.: 32. 1936 syn. sec. WFO 2018

***Dianthuscrassipes* R.Roem.﻿ in Linnaea 25: 11. 1852.** Sec. Bernal et al. (1990)

= *Dianthusserenaeus* Coincy﻿ in J. Bot. (Morot) 12: 53. 1898 syn. sec. Bernal et al. (1990) ≡ Dianthuscrassipessubsp.serenaeus (Coincy) Rivas Mart. et al.﻿ in Rivasgodaya 6: 29. 1991 syn. sec. this publication

***Dianthuscrenatus* Thunb.﻿, Prodr. Pl. Cap. 1: 81. 1794.** Sec. African Plant Database (version 3.4.0)

= *Dianthusinaequalis* E.Mey.﻿, Zwei Pflanzengeogr. Dokum.: 178. 1843 syn. sec. WFO 2018

***Dianthuscretaceus* Adams﻿ in Beitr. Naturk. 1: 56. 1805.** Sec. Kuzmina & Nersesyan (2012)

= *Dianthuspetraeus* M.Bieb.﻿, Fl. Taur.-Caucas. 1: 328. 1808 syn. sec. Kuzmina & Nersesyan (2012)

= *Dianthusbracteatus* Willd. ex Ser.﻿, Prodr. 1: 360. 1824 syn. sec. Kuzmina & Nersesyan (2012)

= *Dianthusliboschitzianus* Ser.﻿, Prodr. 1: 360. 1824 syn. sec. Kuzmina & Nersesyan (2012)

= *Dianthussiemienkiewiczii* Bordz.﻿ in Repert. Spec. Nov. Regni Veg. 30: 364. 1932 syn. sec. this publication

**Dianthuscretaceussubsp.cretaceus**﻿

**Dianthuscretaceussubsp.dmanissianus (M.Kuzmina) Nersesian﻿ in Nov. Syst. Vyssh. Rast. 42: 113. 2011.** Sec. Kuzmina & Nersesyan (2012)

≡ *Dianthusdmanissianus* M.Kuzmina﻿ in Bot. Zhurn. (Moscow & Leningrad) 81(8): 80. 1996 syn. sec. Kuzmina & Nersesyan (2012)

**Dianthuscretaceussubsp.multicaulis (Boiss. & A.Huet) Nersesian﻿ in Novosti Sist. Vyssh. Rast. 42: 112. 2011.** Sec. Kuzmina & Nersesyan (2012)

≡ *Dianthusmulticaulis* Boiss. & A.Huet﻿ in Boissier, Diagn. Pl. Orient., ser. 2, 5: 53. 1856 syn. sec. Kuzmina & Nersesyan (2012) ≡ Dianthusliboschitzianusvar.multicaulis (Boiss. & A.Huet) Boiss.﻿, Fl. Orient. 1: 487. 1867 syn. sec. Kuzmina & Nersesyan (2012) ≡ Dianthuscretaceusvar.multicaulis (Boiss. & A.Huet) Grossh.﻿, Fl. Cauc. 2: 430. 1930 syn. sec. Kuzmina & Nersesyan (2012)

**Dianthuscretaceussubsp.sevanensis Nersesian﻿ in Nov. Syst. Vyssh. Rast. 42: 113. 2011.** Sec. Kuzmina & Nersesyan (2012)

***Dianthuscribrarius* Clementi﻿ in Mem. Reale Accad. Sci. Torino II 16: 20. 1855.** Sec. Marhold (2011)

***Dianthuscrinitus* Sm.﻿ in Trans. Linn. Soc. London 2: 300. 1794.** Sec. Rechinger (1988)

= *Dianthusfimbriatus* Hohen.﻿ in Bull. Soc. Imp. Naturalistes Moscou 6: 232. 1833 syn. sec. Rechinger (1988)

= *Dianthusibericus* Steven ex Ledeb.﻿, Fl. Ross. 1: 283. 1842 syn. sec. Rechinger (1988)

= *Dianthusamoenus* Pomel﻿, Nouv. Mat. Fl. Atl. 1: 210. 1874 syn. sec. Rechinger (1988) ≡ Dianthuscrinitusvar.amoenus (Pomel) Maire﻿, Fl. Afrique N. 10: 300. 1963 syn. sec. Kew WCVP (2019)

= Dianthuscrinitusvar.flaviflorus Emb.﻿ in Bull. Soc. Sci. Nat. Maroc 15: 196. 1935 syn. sec. Kew WCVP (2019)

**Dianthuscrinitussubsp.baldzhuanicus (Lincz.) Rech.f.﻿ in Plant Syst. Evol. 151: 285. 1986.** Sec. Rechinger (1988)

≡ *Dianthusbaldzhuanicus* Lincz.﻿ in Trudy Tadzhikistansk. Bazy 8: 629. 1940 syn. sec. Rechinger (1988)

**Dianthuscrinitussubsp.crinitus**﻿

**Dianthuscrinitussubsp.kermanensis Rech.f.﻿, Pl. Syst. Evol. 151(3–4): 286. 1986.** Sec. Rechinger (1988)

**Dianthuscrinitussubsp.nuristanicus (Gilli) Rech.f.﻿ in Plant Syst. Evol. 151: 285. 1986.** Sec. Rechinger (1988)

≡ *Dianthusnuristanicus* Gilli﻿ in Feddes Repert. Spec. Nov. Regni Veg. 59: 162. 1956 syn. sec. Rechinger (1988)

**Dianthuscrinitussubsp.soongoricus (Schischk.) Kozhevn.﻿ in Novosti Sist. Vyssh. Rast. 22: 112. 1985.** Sec. Rechinger (1988)

≡ *Dianthussoongoricus* Schischk.﻿, Fl. URSS 6: 899. 1936 syn. sec. Rechinger (1988)

**Dianthuscrinitussubsp.tetralepis (Nevski) Rech.f.﻿ in Plant Syst. Evol. 151: 286. 1986.** Sec. Rechinger (1988)

≡ *Dianthustetralepis* Nevski﻿, Fl. URSS 6: 899. 1936 syn. sec. Rechinger (1988)

**Dianthuscrinitussubsp.turcomanicus (Schischk.) Rech.f.﻿ in Plant Syst. Evol. 151: 287. 1986.** Sec. Rechinger (1988)

≡ *Dianthusturcomanicus* Schischk.﻿, Fl. URSS 6: 899. 1936 syn. sec. Rechinger (1988)

**Dianthuscrinitusvar.argaeus Aytaç & H.Duman﻿ in Ann. Bot. Fenn. 41: 217. 2004.** Sec. Rechinger (1988)

***Dianthuscrossopetalus* (Fenzl ex Boiss.) Grossh.﻿, Fl. Kavkaza 2: 428. 1930.** Sec. Kuzmina & Nersesyan (2012)

≡ Dianthuscrinitusvar.crossopetalus Fenzl ex Boiss.﻿, Fl. Orient. 1: 496. 1867 syn. sec. Kuzmina & Nersesyan (2012)

***Dianthuscruentus* Griseb.﻿, Spic. Fl. Rumel. 1: 186. 1843.** Sec. Dimopoulos et al. (2013)

= Dianthuscruentusvar.micropetalus Pančić﻿ in Verh. Zool.-Bot. Vereins Wien 6: 501. 1856 syn. sec. Kew WCVP (2019)

= *Dianthusconsanguineus* Schur﻿, Enum. Pl. Transsilv.: 93. 1866 syn. sec. WFO 2018

= *Dianthusfastigiatus* Pant.﻿ in Verh. Vereins Natur- Heilk. Presburg n.s., 2: 105. 1871 syn. sec. WFO 2018

= *Dianthusholzmannianus* Heldr. & Hausskn. ex Nyman﻿, Consp. Fl. Eur. Suppl. 2(1): 58. 1889 syn. sec. WFO 2018

= Dianthuscibrariusvar.leucolepis Hausskn.﻿ in Mitth. Thüring. Bot. Vereins n.f., 5: 54. 1893 syn. sec. WFO 2018 ≡ *Dianthusbrachyzonus* Borbás ex Formánek﻿ in Verh. Naturf. Vereins Brünn 35: 194. 1897 syn. sec. WFO 2018

= *Dianthusquadrangulus* Velen.﻿ in Sitzungsber. Königl. Böhm. Ges. Wiss., Math.-Naturwiss. Cl. 1892: 372. 1893 syn. sec. WFO 2018

= *Dianthusturcicus* Velen.﻿ in Sitzungsber. Königl. Böhm. Ges. Wiss., Math.-Naturwiss. Cl. 1892: 273. 1893 syn. sec. WFO 2018 ≡ Dianthuscruentussubsp.turcicus (Velen.) Stoj. & Acht.﻿, Fl. Bulg. ed. 3: 405. 1948 syn. sec. WFO 2018

= *Dianthuslateritius* Halácsy﻿, Consp. Fl. Graec. 1: 213. 1900 syn. sec. WFO 2018

= *Dianthusbaldaccii* Degen﻿ in Magyar Bot. Lapok 5: 275. 1906 syn. sec. WFO 2018

= Dianthuscruentusvar.pancicii Stoj. & Acht.﻿, Krit. Stud. Nelk. Bulg.: 56. 1935 syn. sec. WFO 2018

= *Dianthushyalolepis* Acht. & Lindtner﻿ in Izv. Carsk. Prir. Inst. Sofija 13: 195. 1940 syn. sec. WFO 2018

= Dianthuscalocephalusvar.villiger Bornm.﻿ syn. sec. WFO 2018 ≡ *Dianthusvilliger* (Bornm.) Bornm.﻿ in Allg. Bot. Z. Syst. 32: 27. 1926 [“1927”] syn. sec. WFO 2018 ≡ Dianthusbrachyzonussubsp.villiger (Bornm.) Micevski﻿ in Prilozi Oddel. Biol. Med. Nauki Makedonska Akad. Nauk. Umet. 8: 45. 1987 [“1990”] syn. sec. WFO 2018

***Dianthuscyathophorus* Moris﻿, Index Seminum (TO) 1852: 32. 1852.** Sec. Bacchetta et al. (2010)

≡ Dianthussiculussubsp.cyathophorus (Moris) Arrigoni﻿ in Parlatorea 7: 20. 2005 syn. sec. Bacchetta et al. (2010)

= *Dianthusminae* Mazzola, Raimondo & Ilardi﻿ in Bocconea 17: 307. 2004 syn. sec. Bacchetta et al. (2010) ≡ Dianthuscyathophorussubsp.minae (Mazzola, Raimondo & Ilardi) Raimondo﻿ in Quad. Bot. Amb. Appl. 21: 189. 2010 syn. sec. this publication

***Dianthuscyprius* A.K.Jacks. & Turrill﻿ in Bull. Misc. Inform. Kew 1938: 462. 1938.** Sec. Marhold (2011)

***Dianthuscyri* Fisch. & C.A.Mey.﻿ in Index Seminum St. Petersburg (Petropolitanus) 4: 34. 1838.** Sec. Kuzmina & Nersesyan (2012)

***Dianthusdaghestanicus* Kharadze﻿ in Zametki Sist. Geogr. Rast. 16: 47. 1951.** Sec. Kuzmina & Nersesyan (2012)

***Dianthusdarvazicus* Lincz.**﻿ Sec. Czerepanov (1995)

***Dianthusdeltoides* L.﻿, Sp. Pl.: 411. 1753.** Sec. Marhold (2011)

≡ *Caryophyllusdeltoides* (L.) Moench﻿, Methodus: 59. 1794 syn. sec. POWO. Plants of the World Online. Facilitated by the Royal Botanic Gardens, Kew. ≡ *Cylichnanthusdeltoides* (L.) Dulac﻿, Fl. Hautes-Pyrénées: 262. 1867 syn. sec. POWO. Plants of the World Online. Facilitated by the Royal Botanic Gardens, Kew. ≡ *Silenedeltoides* (L.) E.H.L.Krause﻿, Deutschl. Fl. Abbild., ed. 2, 5: 113. 1901 syn. sec. POWO. Plants of the World Online. Facilitated by the Royal Botanic Gardens, Kew.;

= Dianthusdeltoidesvar.montanus Klett & Richt.﻿, Fl. Leipzig: 376. 1830 syn. sec. Kew WCVP (2019)

= *Dianthusalbus* Schkuhr ex Steud.﻿, Nomencl. Bot., ed. 2, 1: 498. 1840 syn. sec. WFO 2018

= *Dianthusendressii* Zahlbr. ex Conrath﻿ in Oesterr. Bot. Z. 38: 51. 1888 syn. sec. WFO 2018

= *Dianthusglaucus* L.﻿, Sp. Pl.: 411. 1753 syn. sec. WFO 2018 ≡ *Caryophyllusglaucus* (L.) Moench﻿, Methodus: 59. 1794 syn. sec. POWO. Plants of the World Online. Facilitated by the Royal Botanic Gardens, Kew. ≡ Dianthusdeltoidesvar.glaucus (L.) Trevir.﻿, Index Seminum (WROCL, Wratislaviensi) 1818: 3. 1818 syn. sec. Kew WCVP (2019) ≡ Dianthusdeltoidesf.glaucus (L.) P.D.Sell﻿, Fl. Gr. Brit. Ireland 1: 686. 2018 syn. sec. Kew WCVP (2019)

= *Dianthussupinus* Lam.﻿, Fl. Franç. 2: 534. 1779 syn. sec. WFO 2018

= *Dianthuscrenatus* Gilib.﻿, Fl. Lit. Inch. 2: 161. 1782 syn. sec. WFO 2018

= *Dianthusvolgensis* Ser.﻿, Prodr. 1: 361. 1824 syn. sec. WFO 2018

**Dianthusdeltoidessubsp.degenii (Bald.) Strid﻿ in Willdenowia 13: 280. 1983 [“1984”].** Sec. Marhold (2011)

≡ *Dianthusdegenii* Bald.﻿ in Nuovo Giorn. Bot. Ital. n.s., 6: 27. 1899 syn. sec. Marhold (2011)

**Dianthusdeltoidessubsp.deltoides**﻿

***Dianthusdenaicus* Assadi﻿ in Iranian J. Bot. 3: 17. 1985.** Sec. Rechinger (1988)

***Dianthusdesideratus* Strid﻿, Fl. Hellenica 1: 371. 1997.** Sec. Dimopoulos et al. (2013)

***Dianthusdiffusus* Sm.﻿, Fl. Graec. Prodr. 1(2): 285. 1809.** Sec. Dimopoulos et al. (2013)

= *Dianthuspubescens* Sm.﻿, Fl. Graec. Prodr. 1(2): 286. 1809 syn. sec. WFO 2018

= *Dianthusrupestris* Friv. ex Griseb.﻿, Spic. Fl. Rumel. 1: 191. 1843 syn. sec. WFO 2018

= *Dianthuscylleneus* Boiss. & Heldr.﻿ in Boissier, Diagn. Pl. Orient., ser. 2, 1: 63. 1854 syn. sec. WFO 2018

= *Dianthussyriacus* F.N.Williams﻿ in J. Bot. 23: 346. 1885 syn. sec. WFO 2018

= *Dianthusglandulosopubescens* Halácsy﻿ in Verh. K. K. Zool.-Bot. Ges. Wien 48: 706. 1898 syn. sec. WFO 2018

***Dianthusdilepis* Rech.f.﻿ in Bot. Jahrb. Syst. 75: 361. 1951.** Sec. Rechinger (1988)

***Dianthusdiversifolius* Assadi﻿ in Iranian J. Bot. 3: 40. 1985.** Sec. Rechinger (1988)

***Dianthusdobrogensis* Prodán﻿ in Bul. Acad. Stud. Agron. Cluj 5(1): 97. 1934.** Sec. Marhold (2011)

**Dianthus×dufftii Hausskn. ex Asch.﻿ in Oesterr. Bot. Z. 26: 259. 1876**

***Dianthusedetanus* (M.B.Crespo & Mateo) M.B.Crespo & Mateo﻿ in Flora Montiber. 40: 64. 2008.** Sec. Mateo Sanz & Crespo (2008)

≡ Dianthushispanicussubsp.edetanus M.B.Crespo & Mateo﻿ in Flora Montiber. 20: 7. 2002 syn. sec. Mateo Sanz & Crespo (2008)

***Dianthuselatus* Ledeb.﻿, Fl. Altaic. 2: 136. 1830.** Sec. Dequan & Turland (2001)

***Dianthuselbrusensis* Kharadze﻿ in Zametki Sist. Geogr. Rast. 21: 48. 1959.** Sec. Kuzmina & Nersesyan (2012)

***Dianthuseldivenus* Czeczott﻿ in Acta Soc. Bot. Poloniae 9: 33. 1932.** Sec. Marhold (2011)

***Dianthuselegans* d’Urv.﻿, Mém. Soc. Linn. Paris 1: 302. 1822.** Sec. Dimopoulos et al. (2013)

= *Dianthusactinopetalus* Fenzl﻿, Pug. Pl. Nov. Syr.: 11. 1842 syn. sec. WFO 2018

= *Dianthuscous* Boiss.﻿, Diagn. Pl. Orient. ser. 1 1: 20. 1843 syn. sec. WFO 2018

= *Dianthuswawrae* Freyn ex Boiss.﻿, Fl. Orient. Suppl.: 79. 1888 syn. sec. WFO 2018

***Dianthuselymaiticus* Hausskn. & Bornm.﻿ in Beih. Bot. Centralbl. 19(2): 213. 1905.** Sec. Rechinger (1988)

***Dianthusengleri* Hausskn. & Bornm.﻿ in Mitt. Geogr. Ges. (Thüringen) Jena 9: 15. 1891.** Sec. Marhold (2011)

***Dianthuseretmopetalus* Stapf﻿ in Denkschr. Kaiserl. Akad. Wiss., Wien. Math.-Naturwiss. Kl. 51: 349. 1886.** Sec. Marhold (2011)

***Dianthuserinaceus* Boiss.﻿, Diagn. Pl. Orient. ser. 1 1: 21. 1843.** Sec. Marhold (2011)

= *Dianthuswebbianus* Parl. ex Vis.﻿ in Atti Riunione Sci. Ital. 2: 180. 1841 syn. sec. Marhold (2011)

***Dianthusernesti-mayeri* Micevski & Matevski﻿ in Razpr. Slov. Akad. Znan. Umetn., Razr. Nar. Vede 42: 155. 2001.** Sec. WFO 2018

***Dianthuserythrocoleus* Boiss.﻿, Fl. Orient. 1: 493. 1867.** Sec. Rechinger (1988)

***Dianthuseugeniae* Kleopow﻿ in Izv. Kievsk. Bot. Sada 12–13: 157. 1931.** Sec. Czerepanov (1995)

= *Dianthustesquicola* Klokov﻿ in Bot. Zhurn. (Kiev) 5: 26. 1948 syn. sec. Czerepanov (1995)

***Dianthusexcelsus* S.S.Hooper﻿ in Hookers Icon. Pl. 37: 13. 1959.** Sec. African Plant Database (version 3.4.0)

= Dianthusangolensissubsp.orientalis Turrill﻿ in Kew Bull. 9: 49. 1954 syn. sec. African Plant Database (version 3.4.0)

***Dianthusfalconeri* Edgew. & Hook.f.﻿, Fl. Brit. India 1: 214. 1874.** Sec. Ghanzafar & Nasir (1986)

**Dianthus×fallens Timb.-Lagr.﻿ in Bull. Soc. Bot. France 5: 329. 1858.** Sec. Bernal et al. (1990)

≡ Dianthus×tenersubsp.fallens (Timb.-Lagr.) Nyman﻿, Consp. Fl. Eur.: 104. 1878 syn. sec. Bernal et al. (1990) ≡ Dianthus×monspessulanusvar.fallens (Timb.-Lagr.) Pau﻿ in Bol. Soc. Aragonesa Ci. Nat. 4: 187. 1905 syn. sec. Bernal et al. (1990)

= Dianthus×borderei Rouy & Foucaud﻿, Fl. France 3: 183. 1896 syn. sec. Kew WCVP (2019) – Dianthus×borderi Rouy & Foucaud﻿, Fl. France 3: 183. 1896 syn. sec. Bernal et al. (1990) [is misspelling for Dianthus×borderi Rouy & Foucaud]

***Dianthusfasciculatus* (Boiss.) Fassou, N.Korotkova, Dimop. & Borsch﻿.** Sec. this publication 141

≡ *Veleziafasciculata* Boiss., Diagn. Pl. Orient., ser. 1, 8: 92. 1849

***Dianthusferrugineus* Mill.﻿, Gard. Dict., ed. 8: 9. 1768.** Sec. Marhold (2011)

= *Dianthusbalbisii* Ser.﻿, Prodr. 1: 356. 1824 syn. sec. Marhold (2011) ≡ Dianthuscarthusianorumvar.balbiosii (Ser.) Tanfani﻿, Fl. Ital. 9: 253. 1892 syn. sec. Kew WCVP (2019)

= Dianthusbalbisiivar.paniculatus Ser.﻿, Prodr. 1: 356. 1824 syn. sec. Kew WCVP (2019)

= *Dianthusglaucophyllus* Hornem. ex Ser.﻿, Prodr. 1: 356. 1824 syn. sec. WFO 2018

= *Dianthuspropinquus* Schur﻿, Enum. Pl. Transsilv.: 94. 1866 syn. sec. WFO 2018

= *Dianthusligusticus* Willd. ex Nyman﻿, Consp. Fl. Eur. 1: 103. 1878 syn. sec. WFO 2018

= *Dianthusrosulatus* Borbás ex Nyman﻿, Consp. Fl. Eur. 1: 103. 1878 syn. sec. WFO 2018

= *Dianthusalbaceteanus* Huter﻿ in Oesterr. Bot. Z. 54: 339. 1904 syn. sec. Peruzzi & Gargano (2006)

**Dianthusferrugineussubsp.ferrugineus**﻿

**Dianthusferrugineussubsp.liburnicus (Bartl.) Tutin﻿ in Feddes Repert. Spec. Nov. Regni Veg. 68: 191. 1963.** Sec. Marhold (2011)

≡ *Dianthusliburnicus* Bartl.﻿ in Beitr. Bot. 2: 52. 1825 syn. sec. Marhold (2011) ≡ Dianthusbalbisiisubsp.liburnicus (Bartl.) Pignatti﻿ in Giorn. Bot. Ital. 111: 45. 1977 syn. sec. Marhold (2011)

= Dianthusliburnicusvar.albiflorus Caldesi﻿ in Nuovo Giorn. Bot. Ital. 11: 338. 1879 syn. sec. Kew WCVP (2019)

***Dianthusfloribundus* Boiss.﻿, Asie Min., Bot. 1: 221. 1860.** Sec. Kuzmina & Nersesyan (2012)

= *Dianthusschischkinii* Grossh.﻿ in Bot. Mater. Gerb. Bot. Inst. Komarova Akad. Nauk S.S.S.R. 11: 83. 1949 syn. sec. Kuzmina & Nersesyan (2012)

= *Dianthusworonowii* Schischk.﻿ in sched. herb. LE syn. sec. Kuzmina & Nersesyan 2012 syn. sec. Kuzmina & Nersesyan (2012)

***Dianthusformanekii* Borbás ex Formánek﻿ in Verh. Naturf. Vereins Brünn 32: 39. 1894.** Sec. Dimopoulos et al. (2013)

***Dianthusfragrans* Adams﻿ in Weber u. Mohr, Beitr. Naturk. 1: 56. 1805.** Sec. Kuzmina & Nersesyan (2012)

= *Dianthusfragrans* M.Bieb.﻿, Fl. Taur.-Caucas. 1: 331. 1808 syn. sec. this publication

= *Dianthusliboschitzianus* Hohen. ex Boiss.﻿, Fl. Orient. 1: 491. 1867 syn. sec. Czerepanov (1995)

= *Dianthustichomirovii* Devyatov, Taisumov & Teimurov﻿ in Byull. Moskovsk. Obshch. Isp. Prir., Otd. Biol. n.s. 104(2): 37. 1999 syn. sec. Kuzmina & Nersesyan (2012)

Notes: – Although Bieberstein (1808) didn’t mention Adams (1805) as an author of *D.fragrans* in his 1^st^ volume of the Flora Taurica-Caucasica, the descriptions are very similar and very likely refer to the same species.

***Dianthusfreynii* Vandas﻿ in Sitzungsber. Königl. Böhm. Ges. Wiss., Math.-Naturwiss. Cl. 1889(2): 255. 1890.** Sec. Marhold (2011)

***Dianthusfruticosus* L.﻿, Sp. Pl.: 413. 1753.** Sec. Dimopoulos et al. (2013)

= *Dianthusfrutescens* Houtt.﻿, Nat. Hist. 2(5): 109. 1775 syn. sec. WFO 2018

**Dianthusfruticosussubsp.amorginus Runemark﻿ in Bot. Not. 133(4): 485. 1980.** Sec. Dimopoulos et al. (2013)

**Dianthusfruticosussubsp.carpathus Runemark﻿ in Bot. Not. 133(4): 487. 1980.** Sec. Dimopoulos et al. (2013)

**Dianthusfruticosussubsp.creticus (Tausch) Runemark﻿ in Bot. Not. 133(4): 488. 1980.** Sec. Dimopoulos et al. (2013)

≡ *Dianthuscreticus* Tausch﻿ in Flora 13: 247. 1830 syn. sec. WFO 2018

**Dianthusfruticosussubsp.fruticosus**﻿

**Dianthusfruticosussubsp.karavius Runemark﻿ in Bot. Not. 133(4): 487. 1980.** Sec. Dimopoulos et al. (2013)

**Dianthusfruticosussubsp.occidentalis Runemark﻿ in Bot. Not. 133: 483. 1980.** Sec. Dimopoulos et al. (2013)

**Dianthusfruticosussubsp.rhodius (Rech.f.) Runemark﻿ in Bot. Not. 133: 486. 1980.** Sec. Dimopoulos et al. (2013)

≡ *Dianthusrhodius* Rech.f.﻿ in Denkschr. Akad. Wiss. Wien, Math.-Naturwiss. Kl. 105: 156. 1943 syn. sec. WFO 2018

**Dianthusfruticosussubsp.sitiacus Runemark﻿ in Bot. Not. 133(4): 488. 1980.** Sec. Dimopoulos et al. (2013)

***Dianthusfurcatus* Balb.﻿, Mém. Acad. Sci. Turin, Sci. Phys. 10–11: 13. 1804.** Sec. Marhold (2011)

= *Dianthusalpester* Balb.﻿, Mém. Acad. Sci. Turin, Sci. Phys. 1: 13. 1802 syn. sec. Marhold (2011) ≡ Dianthuscarthusianorumsubsp.alpester (Balb.) Pers.﻿, Syn. Pl. 1: 493. 1805 syn. sec. Kew WCVP (2019)

= *Dianthuspungens* Gren. & Godr.﻿, Fl. France 1: 234. 1847 syn. sec. WFO 2018

**Dianthusfurcatussubsp.dissimilis (Burnat) Pignatti﻿ in Giorn. Bot. Ital. 107: 209. 1973.** Sec. Marhold (2011)

≡ Dianthusfurcatusvar.dissimilis Burnat﻿, Fl. Alpes Marit. 1: 230. 1892 syn. sec. Marhold (2011)

**Dianthusfurcatussubsp.furcatus**﻿

**Dianthusfurcatussubsp.gyspergerae (Rouy) Burnat ex Briq.﻿, Prodr. Fl. Corse 1: 572. 1910.** Sec. Marhold (2011)

≡ *Dianthusgyspergerae* Rouy﻿ in Rev. Bot. Syst. Géogr. Bot. 1: 132. 1903 syn. sec. Marhold (2011)

**Dianthusfurcatussubsp.lereschii (Burnat) Pignatti﻿ in Giorn. Bot. Ital. 107: 209. 1973.** Sec. Marhold (2011)

≡ Dianthusfurcatusvar.lereschii Burnat﻿, Fl. Alpes Marit. 1: 230. 1892 syn. sec. Marhold (2011)

= *Dianthustener* Balb.﻿, Mém. Acad. Sci. Turin, Sci. Phys. 1: 14. 1802 syn. sec. Marhold (2011) ≡ Dianthusstrictusvar.tener (Balb.) Tanfani﻿, Fl. Ital. 9: 272. 1892 syn. sec. Kew WCVP (2019) ≡ Dianthusfurcatussubsp.tener (Balb.) Tutin﻿ in Feddes Repert. Spec. Nov. Regni Veg. 68: 189. 1963 syn. sec. Marhold (2011)

***Dianthusgabrielianiae* Nersesian﻿ in Takhtajania 1: 45. 2011.** Sec. Kuzmina & Nersesyan (2012)

***Dianthusgasparrinii* Guss.﻿, Fl. Sicul. Syn. 1: 479. 1843.** Sec. Bacchetta et al. (2010)

≡ Dianthuscaryophyllussubsp.gasparrinii (Guss.) Arcang.﻿, Comp. Fl. Ital. ed. 2: 306. 1894 syn. sec. Bacchetta et al. (2010)

***Dianthusgeminiflorus* Loisel.﻿, Fl. Gall. 2: 725. 1807.** Sec. Bernal et al. (1990)

≡ Dianthusfurcatussubsp.geminiflorus (Loisel.) Tutin﻿ in Feddes Repert. Spec. Nov. Regni Veg. 68: 189. 1963 syn. sec. WFO 2018

***Dianthusgenargenteus* Bacch., Brullo, Casti & Giusso﻿ in Nordic J. Bot. 28: 145. 2010.** Sec. Bacchetta et al. (2010)

***Dianthusgiganteiformis* Borbás﻿ in Oesterr. Bot. Z. 41: 32. 1891.** Sec. Marhold (2011)

≡ *Dianthussabuletarum* Heuff.﻿ in Oesterr. Bot. Z. 8: 26. 1858 syn. sec. WFO 2018

= *Dianthuspontederae* A.Kern.﻿, Sched. Fl. Exs. Austro-Hung. 2: 539. 1882 [“1883”] syn. sec. WFO 2018 ≡ Dianthuscarthusianorumsubsp.pontederae (A.Kern.) Hegi﻿ in Allg. Bot. Z. Syst. 17: 16. 1911 syn. sec. WFO 2018 ≡ Dianthusgiganteiformissubsp.pontederae (A.Kern.) Soó﻿ in Acta Bot. Acad. Sci. Hung. 15: 339. 1969 [“1970”] syn. sec. WFO 2018 ≡ Dianthussabuletorumsubsp.pontederae (A.Kern.) Holub﻿ in Folia Geobot. Phytotax. 19: 214. 1984 syn. sec. WFO 2018

= Dianthustenuifoliussubsp.serpentini Podp.﻿, Spisy Přír. Fak. Masarykovy Univ. 12: 20. 1922 syn. sec. WFO 2018 ≡ Dianthussabuletorumsubsp.serpentini (Podp.) Holub﻿ in Folia Geobot. Phytotax. 19: 214. 1984 syn. sec. WFO 2018

= *Dianthusurziceniensis* Prodán﻿, Fl. Reipubl. Popul. Roman. 2: 667. 1953 syn. sec. WFO 2018

= *Dianthusdiutinus* Rchb.﻿, Icon. Fl. Germ. Helv. 6: 44. 1844, nom illeg syn. sec. WFO 2018

= Dianthusgiganteiformisvar.comanae Prodán﻿ syn. sec. WFO 2018 ≡ *Dianthuscomanae* (Prodán) Prodán﻿, Fl. Reipubl. Popul. Roman. 2: 284. 1953 syn. sec. WFO 2018

**Dianthusgiganteiformissubsp.giganteiformis**﻿

**Dianthusgiganteiformissubsp.kladovanus (Degen) Soó﻿ in Feddes Repert. 83: 161. 1972.** Sec. Marhold (2011)

≡ *Dianthuskladovanus* Degen﻿ in Magyar Bot. Lapok 4: 122. 1905 syn. sec. Marhold (2011) ≡ Dianthussabuletorumsubsp.kladovanus (Degen) Holub﻿ in Folia Geobot. Phytotax. 19: 214. 1984 syn. sec. Marhold (2011)

***Dianthusgiganteus* d‘Urv.﻿, Mém. Soc. Linn. Paris 1: 301. 1822.** Sec. Dimopoulos et al. (2013)

= *Dianthusintermedius* Boiss.﻿, Fl. Orient. 1: 515. 1867 syn. sec. Marhold (2011)

= *Dianthushaynaldianus* Borbás﻿ in Oesterr. Bot. Z. 38: 144. 1888 syn. sec. Marhold (2011) ≡ Dianthusgiganteussubsp.haynaldianus (Borbás) Tutin﻿ in Feddes Repert. Spec. Nov. Regni Veg. 69: 191. 1963 syn. sec. WFO 2018

= *Dianthussubgiganteus* Borbás ex Formánek﻿ in Verh. Naturf. Vereins Brünn 32: 181. 1894 syn. sec. WFO 2018 ≡ Dianthusgiganteussubsp.subgiganteus (Borbás ex Formánek) Hayek﻿ in Denkschr. Kaiserl. Akad. Wiss., Wien. Math.-Naturwiss. Kl. 94: 138. 1917 syn. sec. WFO 2018

**Dianthusgiganteussubsp.banaticus (Heuff.) Tutin﻿ in Feddes Repert. Spec. Nov. Regni Veg. 68: 191. 1963.** Sec. Marhold (2011)

≡ Dianthuscarthusianorumvar.banaticus Heuff.﻿ syn. sec. Marhold (2011) ≡ *Dianthusbanaticus* (Heuff.) Dörfl.﻿, Exsicc. (Herb. Norm.) 1894: 3018. 1894 syn. sec. Marhold (2011)

**Dianthusgiganteussubsp.croaticus (Borbás) Tutin﻿ in Feddes Repert. Spec. Nov. Regni Veg. 70: 4. 1964.** Sec. Marhold (2011)

≡ *Dianthuscroaticus* Borbás﻿ in Bot. Jahresber. (Just) 4: 1059. 1877 syn. sec. Marhold (2011)

**Dianthusgiganteussubsp.giganteus**﻿

**Dianthusgiganteussubsp.vandasii (Velen.) Stoj. & Acht.﻿, Sborn. Blghar. Akad. Nauk 29: 43. 1935.** Sec. Marhold (2011)

≡ *Dianthusvandasii* Velen.﻿ in Sitzungsber. Königl. Böhm. Ges. Wiss., Math.-Naturwiss. Cl. 1892: 16. 1893 syn. sec. Marhold (2011)

***Dianthusglabriusculus* (Kit.) Borbás﻿ in Verh. Bot. Vereins Prov. Brandenburg 19: 19. 1877.** Sec. Czerepanov (1995)

≡ Dianthuscollinusvar.glabriusculus Kit.﻿ in Linnaea 32: 528. 1863 syn. sec. Czerepanov (1995) ≡ Dianthuscollinussubsp.glabriusculus (Kit.) Thaisz﻿ in Bot. Közlem. 8: 252. 1910 syn. sec. Czerepanov (1995)

= Dianthusglabriusculussubsp.moldavicus Prodán﻿ in Bul. Soc. Sti. Cluj. 10: 158. 1948 syn. sec. Czerepanov (1995) ≡ *Dianthuspiatra-neamtzui* Prodán﻿, Fl. Reipubl. Popul. Roman. 2: 234. 1953 syn. sec. Czerepanov (1995)

= Dianthuscollinussubsp.glabriusculus Soó﻿ syn. sec. Czerepanov (1995)

***Dianthusglacialis* Haenke﻿ in Collectanea 2: 84. 1788.** Sec. Marhold (2011)

≡ Dianthusalpinusvar.glacialis (Haenke) Regel﻿ in Bull. Soc. Imp. Naturalistes Moscou 34(2): 533. 1862, nom. illeg.

= Dianthusglacialisvar.acaulis Ser.﻿, Prodr. 1: 362. 1824 syn. sec. Kew WCVP (2019)

= Dianthusglacialisvar.latifolius Ser.﻿, Prodr. 1: 362. 1824 syn. sec. Kew WCVP (2019)

= Dianthusalpinusvar.subacaulis Roth﻿, Enum. Pl. Phaen. Germ. 1(2): 282. 1827 syn. sec. Kew WCVP (2019)

= Dianthusglacialisf.reducta Fornac.﻿ in Giorn. Bot. Ital. 107: 246. 1973 syn. sec. WFO 2018

**Dianthusglacialissubsp.gelidus (Schott, Nyman & Kotschy) Tutin﻿ in Feddes Repert. Spec. Nov. Regni Veg. 68: 190. 1963.** Sec. Marhold (2011)

≡ *Dianthusgelidus* Schott, Nyman & Kotschy﻿ in Schott, Analecta Bot.: 54. 1854 syn. sec. Marhold (2011)

**Dianthusglacialissubsp.glacialis**﻿

***Dianthusglutinosus* Boiss. & Heldr.﻿ in Boissier, Diagn. Pl. Orient., ser. 2, 1: 61. 1854.** Sec. Dimopoulos et al. (2013)

= *Dianthuspubescens* d’Urv., Mém. Soc. Linn. Paris 1: 303. 1822 syn. sec. WFO 2018

***Dianthusgoekayi* Kaynak, Yılmaz & Daşkın﻿ in Ann. Bot. Fenn. 48: 74. 2011.** Sec. Yılmaz et al. (2011)

***Dianthusgoerkii* Hartvig & Strid﻿ in Bot. Jahrb. Syst. 108: 321. 1987.** Sec. Hamzaoğlu & Koç (2021)

= Dianthusleucophaeusvar.patens Reeve﻿ syn. sec. Hamzaoğlu & Koç (2021)

***Dianthusgracilis* Sm.﻿, Fl. Graec. Prodr. 1(2): 288. 1809.** Sec. Dimopoulos et al. (2013)

**Dianthusgracilissubsp.armerioides (Griseb.) Tutin﻿ in Feddes Repert. Spec. Nov. Regni Veg. 68: 191. 1963.** Sec. Marhold (2011)

≡ Dianthusgracilisvar.armerioides Griseb.﻿, Spic. Fl. Rumel. 1: 190. 1843 syn. sec. Marhold (2011)

= *Dianthussuskalovicii* Adamović﻿ in Oesterr. Bot. Z. 55: 179. 1905 syn. sec. Marhold (2011)

= *Dianthuscallosus* Velen.﻿ in Sitzungsber. Königl. Böhm. Ges. Wiss., Math.-Naturwiss. Cl. 8: 5. 1910 [“1911”] syn. sec. Marhold (2011)

= *Dianthusachtarovii* Stoj. & Kitan.﻿ in Izv. Bulg. Bot. Druzh. 9: 94. 1943 syn. sec. Marhold (2011) ≡ Dianthusgracilissubsp.achtarovii (Stoj. & Kitanov) Tutin﻿ in Feddes Repert. Spec. Nov. Regni Veg. 68: 191. 1963 syn. sec. Marhold (2011)

**Dianthusgracilissubsp.drenowskianus (Rech.f.) Strid﻿ in Willdenowia 13: 281. 1983 [“1984”].** Sec. Marhold (2011)

≡ *Dianthusdrenowskianus* Rech.f.﻿ in Repert. Spec. Nov. Regni Veg. 31: 158. 1932 syn. sec. Marhold (2011)

**Dianthusgracilissubsp.friwaldskyanus (Boiss.) Tutin﻿ in Feddes Repert. Spec. Nov. Regni Veg. 68: 191. 1963.** Sec. Dimopoulos et al. (2013)

≡ *Dianthusfriwaldskyanus* Boiss.﻿, Diagn. Pl. Orient., ser. 2, 1: 65. 1854 syn. sec. Dimopoulos et al. (2013)

**Dianthusgracilissubsp.gracilis**﻿

= *Dianthusalbanicus* Wettst.﻿ in Biblioth. Bot. 26: 34. 1892 syn. sec. Dimopoulos et al. (2013)

= *Dianthusathous* Rech.f.﻿ in Repert. Spec. Nov. Regni Veg. 31: 159. 1932 syn. sec. Marhold (2011)

**Dianthusgracilissubsp.xanthinus (Davidov) Tutin in Feddes Repert. Spec. Nov. Regni Veg. 68: 191. 1963.** Sec. Dimopoulos et al. (2013)

≡ *Dianthusxanthinus* Davidov﻿ in Trav. Soc. Bulg. Sci. Nat. 8: 56. 1915 syn. sec. this publication – *Dianthusxanthianus* Davidov﻿ in Trav. Soc. Bulg. Sci. Nat. 8: 56. 1915 syn. sec. this publication [is orthographic variant for *Dianthusxanthinus* Davidov] – Dianthusgracilissubsp.xanthianus (Davidov) Tutin﻿ in Feddes Repert. Spec. Nov. Regni Veg. 68: 191. 1963 syn. sec. this publication [is orthographic variant for Dianthusgracilissubsp.xanthinus (Davidov) Tutin﻿]

***Dianthusgraminifolius* C.Presl﻿, Fl. Sicul. 1: 147. 1826.** Sec. Bacchetta et al. (2010)

≡ Dianthusarrostivar.graminifolius (C.Presl) Lojac.﻿, Fl. Sicul. 1: 164. 1889 syn. sec. Bacchetta et al. (2010) – Dianthusarrostiivar.graminifolius (C.Presl) Lojac.﻿, Fl. Sicul. 1: 164. 1889 syn. sec. Bacchetta et al. (2010) [is orthographic variant for Dianthusarrostivar.graminifolius (C.Presl) Lojac.]

***Dianthusgraniticus* Jord.﻿, Observ. Pl. Nouv. 7: 13. 1849.** Sec. Marhold (2011)

≡ Dianthushirtussubsp.graniticus (Jord.) Rouy & Foucaud﻿ in Rouy, Fl. France 3: 177. 1896 syn. sec. Marhold (2011)

***Dianthusgratianopolitanus* Vill.﻿, Hist. Pl. Dauphiné 3: 598. 1789.** Sec. Marhold (2011)

= *Dianthuscaesius* Sm.﻿, Engl. Bot. [1]: t. 62. 1792 syn. sec. Marhold (2011) ≡ *Silenecaesia* (Sm.) E.H.L.Krause﻿, Deutschl. Fl. Abbild., ed. 2, 5: 112. 1901, nom. illeg.

= Dianthuscaesiussubsp.adscendens Gaudin﻿, Fl. Helv. 3: 158. 1828 syn. sec. Kew WCVP (2019)

= Dianthuscaesiussubsp.montanus Gaudin﻿, Fl. Helv. 3: 159. 1828 syn. sec. Kew WCVP (2019)

= Dianthuscaesiusvar.nanus Gaudin﻿, Fl. Helv. 3: 159. 1828 syn. sec. Kew WCVP (2019)

= *Dianthusflaccidus* Fieber﻿ in Flora 17: 633. 1834 syn. sec. WFO 2018

***Dianthusgredensis* Pau ex Caball.﻿ in Anales Jard. Bot. Madrid 5: 513. 1945.** Sec. Bernal et al. (1990)

≡ Dianthuslangeanussubsp.gredensis (Pau ex Caball.) Rivas Mart., Fern.Gonz. & Sánchez Mata﻿ in Opusc. Bot. Pharm. Complut. 2: 108. 1986 syn. sec. Bernal et al. (1990) ≡ Dianthuspungenssubsp.gredensis (Pau ex Caball.) Crespí, C.P.Fern., A.Castro, Bernardos & Amich﻿ in Ann. Bot. Fenn. 44: 253. 2007 syn. sec. this publication

***Dianthusgrossheimii* Schischk.﻿ in Trudy Bot. Inst. Akad. Nauk S.S.S.R., ser. 1, Fl. Sist. Vyssh. Rast. 2: 278. 1936.** Sec. Kuzmina & Nersesyan (2012)

***Dianthusguessfeldtianus* Muschl.﻿, Man. Fl. Egypt 1: 330. 1912.** Sec. Marhold (2011)

***Dianthusguliae*Janka﻿ in J. Bot. 12: 338. 1874.** Sec. Peruzzi & Gargano (2006)

≡ Dianthusliburnicusvar.guliae (Janka) Arcang.﻿, Comp. Fl. Ital.: 85. 1882 syn. sec. Peruzzi & Gargano (2006) ≡ Dianthuscarthusianorumvar.guliae (Janka) Tanfani﻿, Fl. Ital. 9: 254. 1892 syn. sec. Peruzzi & Gargano (2006)

***Dianthusguttatus* M.Bieb.﻿, Fl. Taur.-Caucas. 1: 328. 1808.** Sec. Czerepanov (1995)

= *Dianthuspseudogrisebachii* Grecescu﻿, Consp. Fl. Roman.: 100. 1898 syn. sec. WFO 2018

= Dianthusguttatussubsp.mariae Kleopow﻿ in Zhurn. Inst. Bot. Vseukraïns’k. Akad. Nauk 21–22: 244. 1939 syn. sec. WFO 2018 ≡ *Dianthusmariae* (Kleopow) Klokov﻿ in Bot. Zhurn. (Kiev) 5: 27. 1948 syn. sec. WFO 2018

**Dianthusguttatussubsp.dicaricatus Prodán﻿, Fl. Republ. Popul. Român. 2: 669. 1953.** Sec. Jalas & Suominen (1988)

**Dianthusguttatussubsp.guttatus**﻿

***Dianthushaematocalyx* Boiss. & Heldr.﻿ in Boissier, Diagn. Pl. Orient., ser. 2, 1: 68. 1854.** Sec. Dimopoulos et al. (2013)

= Dianthushaematocalyxf.olympica Stoj. & Jordanov﻿ in God. Sofiisk. Univ. Fiz.-Mat. Fak. 34: 178. 1938 syn. sec. Kew WCVP (2019)

**Dianthushaematocalyxsubsp.haematocalyx**﻿

**Dianthushaematocalyxsubsp.phitosianus Constantin.﻿ in Phyton (Horn) 39(2): 279. 1999.** Sec. Dimopoulos et al. (2013)

**Dianthushaematocalyxsubsp.pindicola (Vierh.) Hayek in Repert. Spec. Nov. Regni Veg. Beih. 30(1): 240. 1924.** Sec. Dimopoulos et al. (2013)

≡ *Dianthuspindicola* Vierh.﻿ in Verh. K. K. Zool.-Bot. Ges. Wien 47: 31. 1897 syn. sec. Dimopoulos et al. (2013) ≡ Dianthushaematocalyxsubsp.pinidicola (Vierh.) Hayek﻿ in Repert. Spec. Nov. Regni Veg. Beih. 30(1): 240. 1924 syn. sec. Dimopoulos et al. (2013)

= Dianthuspineticolavar.jacupicensis Košanin﻿ syn. sec. WFO 2018

**Dianthushaematocalyxsubsp.pruinosus (Boiss. & Orph.) Hayek﻿ in Repert. Spec. Nov. Regni Veg. Beih. 30(1): 240. 1924.** Sec. Dimopoulos et al. (2013)

≡ *Dianthuspruinosus* Boiss. & Orph.﻿ in Boissier, Diagn. Pl. Orient. ser. 2, 6: 28. 1859 syn. sec. Dimopoulos et al. (2013)

**Dianthushaematocalyxsubsp.ventricosus Maire & Petitm.﻿ in Bull. Soc. Sci. Nancy III, 9: 193. 1908.** Sec. Dimopoulos et al. (2013)

≡ *Dianthusventricosus* Heldr. ex Halácsy﻿, Consp. Fl. Graec. 1: 204. 1900, nom. illeg. syn. sec. Marhold (2011)

= *Dianthussibthorpii* Vierh.﻿ in Verh. K. K. Zool.-Bot. Ges. Wien 47: 33. 1897 syn. sec. Marhold (2011) ≡ Dianthushaematocalyxsubsp.sibthorpii (Vierh.) Hayek﻿ in Repert. Spec. Nov. Regni Veg. Beih. 30(1): 240. 1924 syn. sec. Marhold (2011)

***Dianthushafezii* Assadi﻿ in Iranian J. Bot. 3: 23. 1985.** Sec. Rechinger (1988)

***Dianthushalisdemirii* Hamzaoğlu & Koç﻿ in KSÜ Tarim Doga Derg. 21: 548. 2018.** Sec. Hamzaoğlu et al. (2018)

***Dianthushamzaoglui* Koç﻿ in Phytotaxa 439(1): 58. 2020.** Sec. Koç (2020)

***Dianthusharrissii* Rech.f.﻿ in Plant Syst. Evol. 142: 240. 1983.** Sec. Rechinger (1988)

***Dianthushelenae* Vved.﻿ in Bot. Mater. Gerb. Bot. Inst. Uzbekistansk. Fil. Akad. Nauk S.S.S.R. 3: 10. 1941.** Sec. Czerepanov (1995)

**Dianthus×hellwigii Borbás ex Čelak.﻿ in Sitzungsber. Königl. Böhm. Ges. Wiss., Math.-Naturwiss. Cl. 1878: 20. 1879.** Sec. IPNI

≡ Dianthus×hellwigii Borbás ex Asch.﻿ in Oesterr. Bot. Z. 26: 258. 1876;

**Dianthus×helveticorum M.Laínz﻿ in Anales Jard. Bot. Madrid 42: 549. 1985 [“1986”].** Sec. Bernal et al. (1990)

***Dianthushenteri* Heuff. ex Griseb. & Schenk﻿ in Arch. Naturgesch. 18(1): 303. 1852.** Sec. Marhold (2011)

***Dianthushispidus* (Boiss. & Balansa) Fassou, N.Korotkova, Dimop. & Borsch.** Sec. this publication 150

≡ *Veleziahispida* Boiss. & Balansa﻿ in Boissier, Diagn. Pl. Orient., ser. 2, 5: 57. 1856 syn. sec. this publication

***Dianthushoeltzeri* C.Winkl.﻿ in Gartenflora 30: 1. 1881.** Sec. Dequan & Turland (2001)

***Dianthusholopetalus* Turcz.﻿ in Bull. Soc. Imp. Naturalistes Moscou 27(2): 369. 1854.** Sec. African Plant Database (version 3.4.0)

***Dianthushumilis* Willd. ex Ledeb.﻿, Fl. Ross. 1: 280. 1842.** Sec. Czerepanov (1995)

= *Dianthushirtus* M.Bieb.﻿, Fl. Taur.-Caucas. 1: 326. 1808 syn. sec. WFO 2018

= *Dianthusvirgineus* Hablitz ex M.Bieb.﻿, Fl. Taur.-Caucas. 1: 326. 1808 syn. sec. WFO 2018

= *Dianthussterilis* Steven ex Boiss.﻿, Fl. Orient. 1: 505. 1867 syn. sec. WFO 2018

***Dianthushymenolepis* Boiss.﻿, Diagn. Pl. Orient., ser. 1, 8: 64. 1849.** Sec. Rechinger (1988)

***Dianthushypanicus* Andrz.﻿, Ischisl. Rast. Podolsk. Gub. 1: 18. 1860.** Sec. Czerepanov (1995)

***Dianthushyrcanicus* Rech.f.﻿ in Plant Syst. Evol. 142: 241. 1983.** Sec. Rechinger (1988)

***Dianthushyssopifolius* L.﻿, Cent. Pl. I: 11. 1755.** Sec. Bernal et al. (1990)

= *Dianthusmonspeliacus* L.﻿, Syst. Nat., ed. 10 2: 1029. 1759 syn. sec. WFO 2018

= *Dianthusambiguus* Salisb.﻿, Prodr. Stirp. Chap. Allerton: 303. 1796 syn. sec. WFO 2018

= *Dianthussaxatilis* Pers.﻿, Syn. Pl. 1: 494. 1805 syn. sec. WFO 2018

= *Dianthusalpestris* Sternb.﻿, Deutschl. Fl.: 28. 1809 syn. sec. WFO 2018 ≡ Dianthusmonspessulanusvar.alpestris (Sternb.) Arcang.﻿, Comp. Fl. Ital.: 87. 1882 syn. sec. Kew WCVP (2019)

= *Dianthusplumosus* DC. ex Spreng.﻿, Pl. Min. Cogn. Pug. 2: 64. 1815 syn. sec. WFO 2018 ≡ Dianthusmonspessulanusvar.plumosus (DC. ex Spreng.) Gaudin﻿, Fl. Helv. 6: 355. 1830 syn. sec. Kew WCVP (2019)

= *Dianthussuaveolens* Spreng.﻿, Novi Provent.: 16. 1818 syn. sec. Bernal et al. (1990) ≡ Dianthusmonspessulanusvar.suaveolens (Spreng.) Trevir.﻿, Index Seminum (WROCL, Wratislaviensi) 1821(App. 3): 1. 1821 syn. sec. Kew WCVP (2019)

= Dianthusmonspessulanusvar.brevifolius Ser.﻿, Prodr. 1: 365. 1824 syn. sec. Kew WCVP (2019)

= *Dianthusacuminatus* Tausch﻿, Syll. Pl. Nov. 2: 242. 1828 syn. sec. WFO 2018

= *Dianthuscontroversus* Gaudin, Fl. Helv. 3: 157. 1828 syn. sec. WFO 2018 ≡ Dianthusseguierivar.controversus (Gaudin) W.D.J.Koch﻿, Syn. Fl. Germ. Helv. 1: 96. 1835 syn. sec. Kew WCVP (2019) ≡ Dianthusseguierisubsp.controversus (Gaudin) Arcang.﻿, Comp. Fl. Ital.: 84. 1882 syn. sec. Kew WCVP (2019)

= *Dianthussprengelii* G.Don﻿, Gen. Hist. 1: 394. 1831 syn. sec. WFO 2018

= *Dianthusodoratissimus* Vest ex Rchb.﻿, Fl. Germ. Excurs.: 807. 1832 syn. sec. WFO 2018

= Dianthusmonspessulanusvar.alpicola W.D.J.Koch﻿, Syn. Fl. Germ. Helv. 1: 99. 1835 syn. sec. Kew WCVP (2019)

= *Dianthuscondensatus* Kil.﻿ in Linnaea 32: 532. 1863 syn. sec. WFO 2018

= *Dianthusoreades* Balb. ex Nyman﻿, Consp. Fl. Eur. 1: 104. 1878 syn. sec. WFO 2018

= *Dianthuseynensis* Sennen﻿ in Bol. Soc. Ibér. Ci. Nat. 25: 148. 1926 syn. sec. WFO 2018

= Dianthusmonspessulanusvar.jacetanus P.Monts.﻿ in Bull. Soc. Échange Pl. Vasc. Eur. Occid. Bassin Médit. 18: 72. 1981 syn. sec. WFO 2018

**Dianthushyssopifoliussubsp.gallicus (Pers.) M.Laínz & Muñoz Garm﻿ in Anales Jard. Bot. Madrid 44: 572. 1987.** Sec. Bernal et al. (1990)

≡ *Dianthusgallicus* Pers.﻿, Syn. Pl. 1: 495. 1805 syn. sec. Bernal et al. (1990) ≡ Dianthusmonspeliacussubsp.gallicus (Pers.) M.Laínz & Muñoz Garm﻿ in Anales Jard. Bot. Madrid 42: 259. 1985 syn. sec. Bernal et al. (1990)

**Dianthushyssopifoliussubsp.hyssopifolius**﻿

***Dianthusichnusae* Bacch., Brullo, Casti & Giusso﻿ in Nordic J. Bot. 28: 146. 2010.** Sec. Bacchetta et al. (2010)

**Dianthusichnusaesubsp.ichnusae**﻿

**Dianthusichnusaesubsp.toddei Bacch., Brullo, Casti & Giusso﻿ in Nordic J. Bot. 28: 147. 2010.** Sec. Bacchetta et al. (2010)

***Dianthusillyricus* (Ard.) Fassou, N.Korotkova, Dimop. & Borsch﻿.** Sec. this publication 151

≡ *Saponariaillyrica* Ard.﻿, Animadv. Bot. Spec. Alt.: 24. 1764 syn. sec. this publication ≡ *Tunicaillyrica* (Ard.) Fisch. & C.A.Mey.﻿, Index Sem. Hort. Petrop. 4: 49. 1838 syn. sec. this publication ≡ *Fiedleriaillyrica* (Ard.) Rchb.﻿, Icon. Fl. Germ. Helv. 6: 42, t. 246. 1844 syn. sec. this publication (2019) ≡ *Petrorhagiaillyrica* (Ard.) P.W.Ball & Heywood﻿ in Bull. Brit. Mus. (Nat. Hist.), Bot. 3: 133. 1964 syn. sec. this publication

**Dianthusillyricussubsp.angustifolius (Poir.) Fassou, N.Korotkova, Dimop. & Borsch﻿.** Sec. this publication 152

≡ *Sileneangustifolia* Poir.﻿, Voy. Barbarie 2: 164. 1789 syn. sec. this publication (2019); ≡ *Tunicaangustifolia* (Poir.) Briq.﻿, Prodr. Fl. Corse 1: 544. 1910 syn. sec. this publication ≡ Tunicaillyricasubsp.angustifolia (Poir.) Maire﻿ in Bull. Soc. His. Nat. Afrique N. 30: 265. 1939 syn. sec. this publication

= *Gypsophilacompressa* Desf.﻿, Fl. Atlant. 1: 343, t. 97. 1798 syn. sec. this publication ≡ *Tunicacompressa* (Desf.) Fisch. & C.A.Mey.﻿, Index Sem. Hort. Petrop. 4: 50. 1838 syn. sec. this publication ≡ *Dianthellacompressa* (Desf.) Pomel﻿, Mat. Fl. Atl.: 9. 1860 syn. sec. this publication (2019)

= *Tunicadavaeana* Coss.﻿ in Bull. Soc. Bot. France 36: 103. 1889 syn. sec. this publication

= *Tunicascoparia* Pamp.﻿ in Arch. Bot. (Forlì) 12: 25. 1936 syn. sec. this publication (2019)

**Dianthusillyricussubsp.haynaldianus (Janka) Fassou, N.Korotkova, Dimop. & Borsch﻿.** Sec. this publication 152

≡ *Gypsophilahaynaldiana*Janka﻿ in Oesterr. Bot. Z. 20: 316. 1870 syn. sec. this publication (2019) ≡ *Tunicahaynaldiana* (Janka) Borbás﻿ in Math. Term. Közlem. 12: 165. 1876 syn. sec. this publication (2019) ≡ *Gypsophilahaynaldiana*Janka ex Nyman﻿, Consp. Fl. Eur. 1: 100. 1878 syn. sec. IPNI ≡ Tunicaillyricavar.haynaldiana (Janka) Hayek﻿, Prodr. Fl. Penins. Balcan. 1: 222. 1924 syn. sec. this publication ≡ Tunicaillyricasubsp.haynaldiana (Janka) Prodán﻿ in Savulescu, F. Roman. P. R. 2: 215. 1953 syn. sec. this publication ≡ Petrorhagiaillyricasubsp.haynaldiana (Janka) P.W.Ball & Heywood﻿ in Bull. Brit. Mus. (Nat. Hist.), Bot. 3: 134. 1964 syn. sec. this publication

= *Tunicarhodopea* Velen.﻿ in Abh. Böhm. Ges. Wiss. 1894 (29): 4. 1895 syn. sec. Kew WCVP (2019)

**Dianthusillyricussubsp.illyricus**﻿

**Dianthusillyricussubsp.taygeteus (Boiss.) Fassou, N.Korotkova, Dimop. & Borsch﻿.** Sec. this publication 152

≡ Tunicaillyricavar.taygetea Boiss.﻿, Fl. Orient. 1: 521. 1867 syn. sec. this publication; ≡ *Tunicataygetea* (Boiss.) P.H.Davis﻿ in Notes Roy. Bot. Gard. Edinburgh 22: 165. 1957 syn. sec. this publication (2019) ≡ Petrorhagiaillyricasubsp.taygetea (Boiss.) P.W.Ball & Heywood﻿ in Bull. Brit. Mus. (Nat. Hist.), Bot. 3: 137. 1964 syn. sec. this publication ≡ Tunicacreticavar.taygetea (Boiss.) Halácsy﻿ syn. sec. this publication

***Dianthusimereticus* (Rupr.) Schischk.﻿ in Byull. Gosud. Muz. Gruzii 5: 123. 1928 [“1930”].** Sec. Kuzmina & Nersesyan (2012)

≡ Dianthusmontanusf.imereticus Rupr.﻿, Fl. Caucasi: 173. 1869 syn. sec. Kuzmina & Nersesyan (2012)

= *Dianthuscharadzeae* Gagnidze & Gvin.﻿ in Zametki Sist. Geogr. Rast. 37: 25. 1981 syn. sec. Kuzmina & Nersesyan (2012)

***Dianthusinamoenus* Schischk.﻿, Fl. URSS 6: 897. 1936.** Sec. Kuzmina & Nersesyan (2012)

= *Dianthuslenkoranicus* Kharadze﻿ in Zametki Sist. Geogr. Rast. 16: 46. 1951 syn. sec. Kuzmina & Nersesyan (2012)

= Dianthuspallenssubsp.inamoenus Sanda﻿ syn. sec. Kuzmina & Nersesyan (2012)

***Dianthusingoldbyi* Turrill﻿ in Bull. Misc. Inform. Kew 1924: 314. 1924.** Sec. Dimopoulos et al. (2013)

***Dianthusinsularis* Bacch., Brullo, Casti & Giusso﻿ in Nordic J. Bot. 28: 156. 2010.** Sec. Bacchetta et al. (2010)

***Dianthusinteger* Vis. in Flora 12(1 Erg.): 11. 1829.** Sec. Dimopoulos et al. (2013)

≡ Dianthuspetraeussubsp.integer (Vis.) Tutin﻿ in Feddes Repert. Spec. Nov. Regni Veg. 68: 190. 1963 syn. sec. WFO 2018

= *Dianthusnicolai* Beck & Szyszył.﻿, Pl. Cernagor. Lect.: 65. 1888 syn. sec. WFO 2018

= *Dianthusprenjus* Beck﻿ in Wiss. Mitt. Bosnien & Herzegovina 11: 488. 1909 syn. sec. WFO 2018

**Dianthusintegersubsp.integer**﻿

**Dianthusintegersubsp.macedonicus Trinajstić﻿ in Suppl. Fl. Anal. Jugosl. 6: 8. 1979.** Sec. Marhold (2011)

– Dianthusintegersubsp.macedonicus Trinajstić﻿ in Suppl. Fl. Anal. Jugosl. 5: 737. 1979, nom. inval. syn. sec. this publication

**Dianthusintegersubsp.minutiflorus (Halácsy) Bornm. ex Strid﻿, Mount. Fl. Greece 1: 182. 1986.** Sec. Dimopoulos et al. (2013)

≡ *Dianthusminutiflorus* Halácsy﻿, Consp. Fl. Graec. 1: 216. 1900 syn. sec. Marhold (2011) ≡ Dianthuspetraeussubsp.minutiflorus (Halácsy) Greuter & Burdet﻿ in Willdenowia 12: 187. 1982 syn. sec. Marhold (2011)

= Dianthusstrictusvar.minutiflorus Borbás﻿ in Verh. Naturf. Vereins Brünn 34: 85. 1896 syn. sec. WFO 2018

***Dianthusintegerrimus* Bunge﻿ in Mém. Acad. Imp. Sci. St-Pétersbourg, Sér. 6, Sci. Math., Seconde Pt. Sci. Nat. 7: 583. 1858.** Sec. Kuzmina & Nersesyan (2012)

≡ Dianthusliboschitzianusvar.integerrimus (Bunge) Boiss.﻿, Fl. Orient. 1: 487. 1867 syn. sec. Kuzmina & Nersesyan (2012) ≡ Dianthuscretaceusvar.integerrimus (Bunge) Grossh.﻿, Fl. Cauc. 2: 430. 1930 syn. sec. Kuzmina & Nersesyan (2012)

***Dianthusjacobsii* Rech.f.﻿ in Plant Syst. Evol. 142: 243. 1983.** Sec. Kuzmina & Nersesyan (2012)

***Dianthusjacquemontii* Edgew. & Hook.f.﻿, Fl. Brit. India 1: 214. 1874.** Sec. Ghanzafar & Nasir (1986)

**Dianthus×jaczonis Asch.﻿ in Oesterr. Bot. Z. 26: 257. 1876.** Sec. POWO. Plants of the World Online. Facilitated by the Royal Botanic Gardens, Kew.

***Dianthusjapigicus* Bianco & S.Brullo﻿ in Braun-Blanquetia 2: 31. 1988.** Sec. Bacchetta et al. (2010)

***Dianthusjaponicus* Thunb.﻿, Syst. Veg. ed. 14: 417. 1784.** Sec. Zoku (1965)

= *Dianthusellipticus* Turcz.﻿ in Bull. Soc. Imp. Naturalistes Moscou 27(2): 369. 1854 syn. sec. Zoku (1965)

= *Dianthusnipponicus* Makino﻿ in Bot. Mag. (Tokyo) 17: 58. 1903 syn. sec. Zoku (1965)

= Dianthusjaponicusf.albiflorus J.Ohara ex Nakan.﻿ in J. Geobot. 16: 116. 1968 syn. sec. WFO 2018

= Dianthusjaponicusf.albiflorus Honda & Koike﻿ in J. Jap. Bot. 46: 75. 1971, nom. illeg. syn. sec. WFO 2018

= Dianthusjaponicusf.albiflorus Konta & S.Matsumoto﻿ in Bull. Natl. Sci. Mus., Tokyo, B. 31(1): 23. 2005, nom. illeg. syn. sec. Zoku (1965)

***Dianthusjaroslavii* Galushko﻿ in Novosti Sist. Vyssh. Rast. 6: 213. 1970.** Sec. Kuzmina & Nersesyan (2012)

**Dianthus×javorkae Kárpáti﻿ in Borbásia 5–6: 87. 1946.** Sec. POWO. Plants of the World Online. Facilitated by the Royal Botanic Gardens, Kew.

***Dianthusjuniperinus* Sm.﻿ in Trans. Linn. Soc. London 2: 303. 1794.** Sec. Dimopoulos et al. (2013)

**Dianthusjuniperinussubsp.aciphyllus (Sieber ex Ser.) Turland﻿ in Bull. Brit. Mus. (Nat. Hist.), Bot. 22: 168. 1992.** Sec. Dimopoulos et al. (2013)

≡ *Dianthusaciphyllus* Sieber ex Ser.﻿ in Candolle, Prodr. 1: 358. 1824 syn. sec. WFO 2018

**Dianthusjuniperinussubsp.bauhinorum (Greuter) Turland﻿ in Bull. Brit. Mus. (Nat. Hist.), Bot. 22: 168. 1992.** Sec. Dimopoulos et al. (2013)

≡ Dianthusaciphyllusvar.bauhinorum Greuter﻿ in Candollea 20: 190. 1965 syn. sec. WFO 2018

– *Dianthusarboreus* L.﻿, Sp. Pl.: 413. 1753, nom. rej. syn. sec. WFO 2018

**Dianthusjuniperinussubsp.heldreichii Greuter﻿ in Candollea 20: 187. 1966.** Sec. Dimopoulos et al. (2013)

**Dianthusjuniperinussubsp.idaeus Turland﻿ in Bull. Brit. Mus. (Nat. Hist.), Bot. 22(2): 168. 1992.** Sec. Dimopoulos et al. (2013)

**Dianthusjuniperinussubsp.juniperinus**﻿

**Dianthusjuniperinussubsp.kavusicus Turland﻿ in Bull. Brit. Mus. (Nat. Hist.), Bot. 22(2): 168. 1992.** Sec. Dimopoulos et al. (2013)

**Dianthusjuniperinussubsp.pulviniformis (Greuter) Turland﻿ in Bull. Brit. Mus. (Nat. Hist.), Bot. 22: 166. 1992.** Sec. Dimopoulos et al. (2013)

≡ *Dianthuspulviniformis* Greuter﻿ in Candollea 20: 189. 1965 syn. sec. WFO 2018

***Dianthusjuzeptchukii* M.Kuzmina﻿ in Bot. Zhurn. (Moscow & Leningrad) 81: 80. 1996.** Sec. Kuzmina & Nersesyan (2012)

***Dianthuskapinaensis* Markgr. & Lindtner﻿ in Glasn. Skopsk. Naucn. Drustva 18(6): 125. 1938.** Sec. Marhold (2011)

***Dianthuskarami* (Boiss.) Mouterde﻿ in Bull. Soc. Bot. France 104: 511. 1958.** Sec. Marhold (2011)

≡ Dianthushypochlorusvar.karami Boiss.﻿, Fl. Orient. Suppl.: 80. 1888 syn. sec. Marhold (2011)

***Dianthuskarataviensis* Pavlov﻿ in Sovetsk. Bot. 1934(1): 22. 1934.** Sec. Czerepanov (1995)

***Dianthuskastembeluensis* Freyn & Sint.﻿ in Oesterr. Bot. Z. 43: 375. 1893.** Sec. Marhold (2011)

***Dianthuskhamiesbergensis* Sond.﻿, Fl. Cap. 1: 124. 1860.** Sec. African Plant Database (version 3.4.0)

***Dianthuskirghizicus* Schischk.﻿, Fl. URSS 6: 896. 1936.** Sec. Czerepanov (1995)

***Dianthuskiusianus* Makino﻿ in Bot. Mag. (Tokyo) 26: 178. 1912.** Sec. Zoku (1965)

= *Dianthushachijoensis* Nakai﻿ in Bot. Mag. (Tokyo) 35: 150. 1921 syn. sec. Zoku (1965)

***Dianthusklokovii* Knjasev﻿ in Bot. Zhurn. (Moscow & Leningrad) 82(11): 80. 1997.** Sec. Knjasev (1997)

***Dianthusknappii* (Pant.) Asch. & Kanitz ex Borbás, Mat. Term. Közlem. 13: 196. 1877.** Sec. Marhold (2011)

≡ Dianthusliburnicusvar.knappii Pant.﻿ in Oesterr. Bot. Z. 23: 4. 1873 syn. sec. Marhold (2011) ≡ Dianthusbalbisiisubsp.knappii (Pant.) Peruzzi & Uzunov﻿ in Phytol. Balcan. 14: 48. 2008 syn. sec. Marhold (2011)

***Dianthuskoreanus*D.C.Son & K.H.Lee﻿ in Phytotaxa 303(1): 71. 2017.** Sec. Son et al. (2017)

***Dianthuskubanensis* Schischk.﻿ in Trudy Bot. Inst. Akad. Nauk S.S.S.R., ser. 1, Fl. Sist. Vyssh. Rast. 3: 184. 1937.** Sec. Kuzmina & Nersesyan (2012)

***Dianthuskuschakewiczii* Regel & Schmalh.﻿ in Trudy Imp. S.-Peterburgsk. Bot. Sada 5: 244. 1877.** Sec. Dequan & Turland (2001)

***Dianthuskusnezowii* Marcow.﻿ in Bot. Mater. Gerb. Glavn. Bot. Sada RSFSR 4: 31. 1923.** Sec. Kuzmina & Nersesyan (2012)

= *Dianthusabchasicus* Gvin.﻿ in Zametki Sist. Geogr. Rast. 27: 78. 1969 syn. sec. Kuzmina & Nersesyan (2012)

***Dianthuslactiflorus* Fenzl﻿ in Asie Min., Bot. 1: 215. 1860.** Sec. Marhold (2011)

***Dianthuslaingsburgensis* S.S.Hooper﻿ in Hookers Icon. Pl. 37: 30. 1959.** Sec. African Plant Database (version 3.4.0)

***Dianthuslanceolatus* Steven ex Rchb.﻿, Pl. Crit. 6: 34. 1828.** Sec. Kuzmina & Nersesyan (2012)

***Dianthuslangeanus* Willk.﻿, Prodr. Fl. Hispan. 3: 690. 1878.** Sec. Bernal et al. (1990)

***Dianthuslaricifolius* Boiss. & Reut.﻿, Diagn. Pl. Nov. Hisp.: 7. 1842.** Sec. Bernal et al. (1990)

**Dianthuslaricifoliussubsp.caespitosifolius (Planellas) M.Laínz﻿, Aport. Conocim. Fl. Gallega 6: 6. 1968.** Sec. Bernal et al. (1990)

≡ *Dianthuscaespitosifolius* Planellas﻿, Ensayo Fl. Gallega: 118. 1852 syn. sec. Bernal et al. (1990)

= *Dianthusplanellae* Willk.﻿, Icon. Descr. Pl. Nov. 1: 79. 1854 syn. sec. Bernal et al. (1990)

**Dianthuslaricifoliussubsp.laricifolius**﻿

**Dianthuslaricifoliussubsp.marizii (Samp.) Franco﻿ in Ann. Bot. Fenn. 23: 91. 1986.** Sec. Bernal et al. (1990)

≡ Dianthusgraniticusvar.marizii Samp.﻿ in Ann. Sci. Nat. (Oporto) 10: 14. 1906 syn. sec. Bernal et al. (1990) ≡ *Dianthusmarizii* (Samp.) Samp.﻿ in Bol. Soc. Brot. II, 1: 134. 1922 syn. sec. Bernal et al. (1990) ≡ Dianthusmerinoisubsp.marizii (Samp.) Rivas Mart. & Penas﻿ in Itinera Geobot. 15: 700. 2002 syn. sec. WFO 2018 – Dianthusgraniticusvar.maritzii Samp.﻿ in Ann. Sci. Nat. (Oporto) 10: 14. 1906 syn. sec. Kew WCVP (2019) [is misspelling for Dianthusgraniticusvar.marizii Samp.]

**Dianthuslaricifoliussubsp.merinoi (M.Laínz) M.Laínz﻿ in Anales Jard. Bot. Madrid 43: 197. 1986.** Sec. Bernal et al. (1990)

≡ *Dianthusmerinoi* M.Laínz﻿, Comun. I. N. I. A., Sér. Recurs. Nat. 2: 3. 1974 syn. sec. Bernal et al. (1990)

***Dianthuslegionensis* (Willk.) F.N.Williams﻿ in J. Bot. 23: 346. 1885.** Sec. Bernal et al. (1990)

≡ Dianthuslusitanusvar.legionensis Willk.﻿, Prodr. Fl. Hispan. 3: 684. 1878 syn. sec. Bernal et al. (1990)

= Dianthustoletanusvar.cutandae Pau﻿ in Bol. Soc. Ibér. Ci. Nat. 22: 87. 1923 syn. sec. Bernal et al. (1990) ≡ *Dianthuscutandae* (Pau) Pau﻿ in Cavanillesia 1: 63. 1928 syn. sec. Bernal et al. (1990) ≡ Dianthusscabersubsp.cutandae (Pau) Tutin﻿ in Feddes Repert. Spec. Nov. Regni Veg. 68: 190. 1963 syn. sec. Bernal et al. (1990)

***Dianthusleptoloma* Steud. ex A.Rich.﻿, Tent. Fl. Abyss. 1: 42. 1847.** Sec. African Plant Database (version 3.4.0)

= *Dianthusabyssinicus* R.Br.﻿, Voy. Abyss. App: lxiv. 1814 syn. sec. WFO 2018

***Dianthusleptopetalus* Willd.﻿, Enum. Pl.: 468. 1809.** Sec. Kuzmina & Nersesyan (2012)

= *Dianthuspomeridianus* M.Bieb.﻿, Fl. Taur.-Caucas. 1: 329. 1808 syn. sec. Kuzmina & Nersesyan (2012)

= *Dianthuspubescens* Fisch.﻿, Cat. Jard. Gorenki ed. 2: 59. 1812 syn. sec. WFO 2018

= *Dianthusbicolor* Hornem.﻿ in Hort. Bot. Hafn. 1: 407. 1813 syn. sec. WFO 2018

***Dianthusleucophaeus* Sm.﻿, Fl. Graec. Prodr. 1(2): 288. 1809.** Sec. Marhold (2011)

= *Dianthusolympicus* Sibth. ex Boiss.﻿, Fl. Orient. 1: 487. 1867 syn. sec. Kew WCVP (2019)

= *Dianthusilgazensis* Czeczott﻿ in Acta Soc. Bot. Poloniae 9: 32. 1932 syn. sec. Marhold (2011)

***Dianthusleucophoeniceus* Dörfl. & Hayek﻿ in Oesterr. Bot. Z. 70: 13. 1921.** Sec. Dimopoulos et al. (2013)

≡ Dianthusgiganteussubsp.leucophoeniceus (Dörfl. & Hayek) Tutin﻿ in Feddes Repert. Spec. Nov. Regni Veg. 68: 191. 1963 syn. sec. Dimopoulos et al. (2013)

***Dianthuslibanotis* Labill.﻿ in Icon. Pl. Syr. 1: 14. 1791.** Sec. Kuzmina & Nersesyan (2012)

= *Dianthusatomarius* Boiss.﻿, Diagn. Pl. Orient., ser. 1, 8: 71. 1849 syn. sec. Kuzmina & Nersesyan (2012)

***Dianthuslindbergii* Riedl﻿ in Ann. Naturhist. Mus. Wien 65: 30. 1962.** Sec. Rechinger (1988)

***Dianthuslongicalyx* Miq. in J. Bot. Néerl. 1: 127. 1861.** Sec. Dequan & Turland (2001)

= *Dianthusoreadum* Hance﻿ in Ann. Sci. Nat., Bot. V, 5: 207. 1866 syn. sec. Dequan & Turland (2001) ≡ Dianthussuperbusvar.oreadum (Hance) Pamp.﻿ in Nuovo Giorn. Bot. Ital. n.s., 17: 265. 1910 syn. sec. Dequan & Turland (2001)

= Dianthussuperbusf.longicalycinus Maxim.﻿ in Trudy Imp. S.-Peterburgsk. Bot. Sada 11: 64. 1890 syn. sec. Dequan & Turland (2001) ≡ Dianthussuperbusvar.longicalycinus (Maxim.) F.N.Williams﻿ in J. Linn. Soc., Bot. 34: 411. 1899 syn. sec. Dequan & Turland (2001) ≡ Dianthussuperbussubsp.longicalycinus (Maxim.) Kitam.﻿ in Acta Phytotax. Geobot. 20: 205. 1962 syn. sec. WFO 2018

= Dianthussuperbusvar.latifolius Nakai﻿ in Bot. Mag. (Tokyo) 43: 457. 1929 syn. sec. WFO 2018 ≡ Dianthussuperbusf.latifolius (Nakai) Kitag.﻿ in J. Jap. Bot. 40: 138. 1965 syn. sec. WFO 2018

= *Dianthustaiwanensis* Masam.﻿ in Trans. Nat. Hist. Soc. Taiwan 33: 618. 1943 syn. sec. Dequan & Turland (2001) ≡ Dianthussuperbusvar.taiwanensis (Masam.) T.S.Liu & S.S.Ying﻿, Fl. Taiwan 2: 334. 1976 syn. sec. Dequan & Turland (2001)

= Dianthussuperbusf.tricolor Honda﻿ in Acta Phytotax. Geobot. 20: 18. 1962 syn. sec. WFO 2018

= Dianthussuperbusvar.pycnophyllus Kitag.﻿ in J. Jap. Bot. 55: 266. 1980 syn. sec. WFO 2018

***Dianthuslongiglumis* Delile﻿ in Ann. Sci. Nat., Bot. II, 20: 89. 1843.** Sec. African Plant Database (version 3.4.0)

***Dianthuslongivaginatus* Rech.f.﻿ in Plant Syst. Evol. 142: 244. 1983.** Sec. Rechinger (1988)

**Dianthus×lorberi Kubát & Abtová﻿ in Severoceskou Prír. 21: 5. 1988.** Sec. Danihelka et al. (2012)

***Dianthuslucae* Asch.﻿ in Oesterr. Bot. Z. 26: 259. 1876**

= Dianthus×lucaevar.novakii Graebn.﻿, Syn. Mitteleur. Fl. 5(2): 451. 1922 syn. sec. Kew WCVP (2019) ≡ Dianthus×novakii (Graebn.) Novák﻿ in Vestn. Král. Ceské Spolecn. Nauk. Tr. Mat.-Prír. 9: 47. 1927 syn. sec. Kew WCVP (2019)

***Dianthuslusitanus* Brot.﻿, Fl. Lusit. 2: 177. 1805.** Sec. Bernal et al. (1990)

= *Dianthusbolivaris* Sennen﻿ in Butl. Inst. Catalana Hist. Nat. 32: 96. 1932 syn. sec. WFO 2018

= Dianthuslusitanusvar.imberbis Maire﻿ in Bull. Soc. His. Nat. Afrique N. 28: 342. 1937 syn. sec. Kew WCVP (2019)

**Dianthuslusitanussubsp.lusitanus**﻿

**Dianthuslusitanussubsp.sidi-tualii (Font Quer) Dobignard﻿ in J. Bot. Soc. Bot. France 20: 39. 2002.** Sec. African Plant Database (version 3.4.0)

≡ *Dianthussidi-tualii* Font Quer﻿ in Cavanillesia 7: 149. 1935 syn. sec. African Plant Database (version 3.4.0)

= Dianthuslusitanusvar.latifolius Maire﻿ in Bull. Soc. His. Nat. Afrique N. 24: 204. 1933 syn. sec. Kew WCVP (2019)

= Dianthuslusitanusvar.tamarutii Caball.﻿, App. Discurs. Univ. Madrid: 2. 1935 syn. sec. Kew WCVP (2019)

= *Dianthusatlanticus* Romo﻿ in Bot. J. Linn. Soc. 108: 205. 1992 syn. sec. African Plant Database (version 3.4.0)

***Dianthuslydus* Boiss.﻿, Diagn. Pl. Orient. ser. 1 1: 20. 1843.** Sec. Marhold (2011)

***Dianthusmacranthoides* Hausskn. ex Bornm.﻿ in Beih. Bot. Centralbl. 19(2): 213. 1906.** Sec. Rechinger (1988)

***Dianthusmacranthus* Boiss.﻿, Diagn. Pl. Orient. ser. 1 1: 23. 1843.** Sec. Rechinger (1988)

***Dianthusmacroflorus* Hamzaoğlu﻿ in Syst. Bot. 40: 210. 2015.** Sec. Hamzaoğlu et al. (2015)

***Dianthusmainensis* Shaulo & Erst﻿ in Feddes Repert. 122: 345. 2011.** Sec. Shaulo & Erst (2011)

***Dianthusmarschallii* Schischk.﻿ in J. Bot. Gard. Nikita 10(2): 39. 1928.** Sec. Czerepanov (1995)

= *Dianthusbicolor* M.Bieb.﻿, Fl. Taur.-Caucas. 1: 329. 1808 syn. sec. Czerepanov (1995)

= Dianthuspallenssubsp.marschallii Sanda﻿ syn. sec. Czerepanov (1995)

***Dianthusmartuniensis* M.Kuzmina﻿ in Bot. Zhurn. (Moscow & Leningrad) 81(8): 81. 1996.** Sec. Kuzmina & Nersesyan (2012)

***Dianthusmasmenaeus* Boiss.﻿, Diagn. Pl. Orient., ser. 2, 5: 51. 1856.** Sec. Kuzmina & Nersesyan (2012)

= *Dianthusmutabilis* Boiss.﻿ in Ann. Sci. Nat., Bot. IV, 2: 44. 1854 syn. sec. Kuzmina & Nersesyan (2012)

= *Dianthusasperulus* Boiss. & A.Huet﻿ in Boissier, Diagn. Pl. Orient., ser. 2, 5: 51. 1856 syn. sec. Kuzmina & Nersesyan (2012)

***Dianthusmazanderanicus* Rech.f.﻿ in Plant Syst. Evol. 142: 245. 1983.** Sec. Rechinger (1988)

**Dianthus×melandrioides Pau﻿ in Not. Bot. Fl. Españ. 1: 29. 1887.** Sec. Bernal et al. (1990)

= Dianthus×carolipaui M.B.Crespo & Mateo﻿ in Anales Jard. Bot. Madrid 47: 506. 1989 [“1990”] syn. sec. POWO. Plants of the World Online. Facilitated by the Royal Botanic Gardens, Kew.

***Dianthusmembranaceus* Borbás﻿ in Oesterr. Bot. Z. 26: 125. 1876.** Sec. Kuzmina & Nersesyan (2012)

= *Dianthusrehmannii* Blocki﻿ in Kosmos (Lvov) 5: 494. 1880 syn. sec. Kuzmina & Nersesyan (2012)

= *Dianthuspseudobarbatus* Besser ex Blocki﻿ in Deutsche Bot. Monatsschr. 3(9): 131. 1885 syn. sec. Kuzmina & Nersesyan (2012)

***Dianthusmercurii* Heldr.﻿ in Atti Congr. Int. Bot. Firenze 1874: 237. 1876.** Sec. Dimopoulos et al. (2013)

***Dianthusmicranthus* Boiss. & Heldr.﻿ in Boissier, Diagn. Pl. Orient., ser. 1, 8: 69. 1849.** Sec. Marhold (2011)

= *Dianthushaussknechtii* Boiss.﻿, Fl. Orient. 1: 489. 1867 syn. sec. Marhold (2011)

***Dianthusmicrolepis* Boiss.﻿, Diagn. Pl. Orient. ser. 1 1: 22. 1843.** Sec. Marhold (2011)

= *Dianthuspumilus* Friv. ex Boiss.﻿, Diagn. Pl. Orient. ser. 1 1: 22. 1843 syn. sec. WFO 2018

= *Dianthusbrachyanthus* Schur﻿, Enum. Pl. Transsilv.: 96. 1866 syn. sec. WFO 2018

= *Dianthuspumilio* Degen & Urum.﻿ in Magyar Bot. Lapok 13: 177. 1914 [“1915”] syn. sec. Marhold (2011)

= Dianthusmicrolepisf.alba Delip. & Dimitrov﻿ in Feddes Repert. 83: 490. 1972 [“1973”] syn. sec. WFO 2018

= Dianthusmicrolepisvar.degenii Stoj. & Acht.﻿ syn. sec. WFO 2018 ≡ Dianthusmicrolepissubsp.degenii (Stoj. & Acht.) Peev & Zlatkova﻿ in Phytologia Balcan. 1: 38. 1995 syn. sec. WFO 2018

= Dianthusmicrolepisvar.musalae Velen.﻿ syn. sec. WFO 2018 ≡ *Dianthusmusalae* (Velen.) Velen.﻿ in Sitzungsber. Königl. Böhm. Ges. Wiss., Math.-Naturwiss. Cl. 8: 6. 1910 [“1911”] syn. sec. WFO 2018

***Dianthusmicropetalus* Ser.﻿, Prodr. 1: 359. 1824.** Sec. African Plant Database (version 3.4.0)

***Dianthusmoesiacus* Vis. & Pančić﻿ in Mem. Reale Ist. Veneto Sci. 15: 17. 1870.** Sec. Marhold (2011)

≡ Dianthuscarthusianorumvar.moesiacus (Vis. & Pančić) F.N.Williams﻿ in J. Linn. Soc., Bot. 29: 376. 1893 syn. sec. Kew WCVP (2019)

= *Dianthusburgasensis* Tutin﻿ in Feddes Repert. Spec. Nov. Regni Veg. 68: 192. 1963 syn. sec. WFO 2018

**Dianthusmoesiacussubsp.grancarovii (Urum.) Stoj. & Acht.﻿ in Sborn. Bălg. Akad. Nauk. 29(2): 53. 1935.** Sec. Marhold (2011)

≡ *Dianthusgrancarovii* Urum.﻿ in Sborn. Bălg. Akad. Nauk. 22(1): 22. 1926 syn. sec. Marhold (2011) ≡ Dianthusmoesiacusvar.grancarovii Urum. ex Stoj. & Stef.﻿, Fl. Bulg. ed. 2: 368. 1933 syn. sec. this publication

**Dianthusmoesiacussubsp.moesiacus**﻿

**Dianthusmoesiacussubsp.sevlievensis (Degen & Nejceff) Stoj. & Acht.﻿ in Sborn. Bălg. Akad. Nauk. 29(2): 54. 1935.** Sec. Marhold (2011)

≡ Dianthusvandasiivar.sevlievensis Degen & Nejceff﻿ in Period. Spis. Bulg. Knizh. Druzh. 69: 74. 1908 syn. sec. Marhold (2011)

**Dianthusmoesiacussubsp.skobelevii (Velen.) Stoj. & Acht.﻿ in Sborn. Bălg. Akad. Nauk. 29(2): 53. 1935.** Sec. Marhold (2011)

≡ Dianthusmoesiacusvar.skobelevii Velen.﻿, Fl. Bulg. Suppl.: 45. 1898 syn. sec. Marhold (2011)

***Dianthusmonadelphus* Vent.﻿, Choix Pl.: t. 39. 1807.** Sec. Dimopoulos et al. (2013)

= *Dianthusochroleucus* Pers.﻿, Syn. Pl. 1: 494. 1805 syn. sec. WFO 2018

**Dianthusmonadelphussubsp.judaicus (Boiss.) Greuter & Burdet in Willdenowia 12: 186. 1982.** Sec. Marhold (2011)

≡ *Dianthusjudaicus* Boiss.﻿, Diagn. Pl. Orient., ser. 1, 8: 66. 1849 syn. sec. Marhold (2011)

= *Dianthusauraniticus* Post﻿ in J. Linn. Soc., Bot. 24: 422. 1888 syn. sec. Marhold (2011)

**Dianthusmonadelphussubsp.monadelphus**﻿

**Dianthusmonadelphussubsp.pallens (Sm.) Greuter & Burdet﻿ in Willdenowia 12: 187. 1982.** Sec. Dimopoulos et al. (2013)

≡ *Dianthuspallens* Sm.﻿, Fl. Graec. Prodr. 1(2): 286. 1809 syn. sec. WFO 2018

= *Dianthusprocumbens* Vent.﻿, Choix Pl.: Index. 1808 syn. sec. WFO 2018

= *Dianthusrhodopeus* Velen.﻿ in Sitzungsber. Königl. Böhm. Ges. Wiss., Math.-Naturwiss. Cl. 1890(2): 40. 1890 syn. sec. WFO 2018

***Dianthusmonspessulanus* L.﻿, Amoen. Acad. 4: 1313. 1759.** Sec. Marhold (2011)

**Dianthusmonspessulanussubsp.marsicus (Ten.) Novák﻿, Spisy Prir. Fak. Karlovy Univ. 21: 25. 1924.** Sec. Marhold (2011)

≡ *Dianthusmarsicus* Ten.﻿, Fl. Napol. 4: 61. 1830 syn. sec. Marhold (2011) ≡ Dianthusmonspessulanusvar.marsicus (Ten.) Arcang.﻿, Comp. Fl. Ital.: 87. 1882 syn. sec. this publication ≡ Dianthussternbergiisubsp.marsicus (Ten.) Pignatti﻿ in Giorn. Bot. Ital. 107: 209. 1973 syn. sec. Marhold (2011) ≡ Dianthuswaldsteiniisubsp.marsicus (Ten.) Greuter & Burdet﻿ in Willdenowia 12: 188. 1982 syn. sec. Marhold (2011)

**Dianthusmonspessulanussubsp.monspessulanus**﻿

***Dianthusmooiensis* F.N.Williams﻿ in J. Bot. 27: 199. 1889.** Sec. African Plant Database (version 3.4.0)

≡ *Dianthusmoviensis* F.N.Williams﻿ in J. Bot. 27: 199. 1889 syn. sec. African Plant Database (version 3.4.0)

= *Dianthusnelsonii* F.N.Williams﻿ in J. Bot. 27: 200. 1889 syn. sec. WFO 2018

**Dianthusmooiensissubsp.kirkii (Burtt Davy) S.S.Hooper﻿ in Hookers Icon. Pl. 37: 54. 1959.** Sec. African Plant Database (version 3.4.0)

≡ *Dianthuskirkii* Burtt Davy﻿ in Bull. Misc. Inform. Kew 1922: 220. 1922 syn. sec. African Plant Database (version 3.4.0) ≡ Dianthusmoviensissubsp.kirkii (Burtt Davy) S.S.Hooper﻿ in Hookers Icon. Pl. 37: 54. 1959 syn. sec. this publication

**Dianthusmooiensissubsp.mooiensis**﻿

**Dianthusmooiensisvar.dentatus Burtt Davy﻿ in Bull. Misc. Inform. Kew 1922: 222. 1922.** Sec. African Plant Database (version 3.4.0)

– Dianthusmoviensisvar.dentatus Burtt Davy﻿ in Bull. Misc. Inform. Kew 1922: 222. 1922 syn. sec. this publication [is misspelling for Dianthusmooiensisvar.dentatus Burtt Davy﻿]

***Dianthusmoravicus* Kovanda﻿ in Preslia 54: 241. 1982.** Sec. Marhold (2011)

≡ Dianthusgratianopolitanussubsp.moravicus (Kovanda) Holub﻿ in Folia Geobot. Phytotax. 18: 205. 1983 syn. sec. this publication

***Dianthusmorisianus* Vals.﻿ in Boll. Soc. Sarda Sci. Nat. 24: 333. 1985.** Sec. Bacchetta et al. (2010)

≡ Dianthussiculussubsp.morisianus (Vals.) Arrigoni﻿ in Parlatorea 7: 20. 2005 syn. sec. Bacchetta et al. (2010)

***Dianthusmossanus* Bacch. & Brullo﻿ in Portugaliae Act. Biol., Sér. B, Sist. 19: 296. 2000.** Sec. Bacchetta & Brullo (2000)

≡ Dianthussiculussubsp.mossanus (Bacch. & Brullo) Arrigoni﻿ in Parlatorea 7: 20. 2005 syn. sec. WFO 2018

***Dianthusmuglensis* Hamzaoğlu & Koç﻿ in Phytotaxa 491(4): 291. 2021.** Sec. Hamzaoğlu & Koç (2021)

≡ Dianthuselegansvar.gramineus R.M.Burton﻿ in Karaca Arbor. Mag. 3: 184. 1997 syn. sec. Hamzaoğlu & Koç (2021)

***Dianthusmultiaffinis* Pau﻿ in Bol. Real Soc. Esp. Hist. Nat. 21: 142. 1921.** Sec. Bernal et al. (1990)

≡ Dianthuspungensvar.multiaffinis (Pau) O.Bolòs & Vigo﻿ in Butl. Inst. Catalana Hist. Nat., Secc. Bot. 38(1): 88. 1974 syn. sec. Bernal et al. (1990) ≡ Dianthuscostaesubsp.multiaffinis (Pau) M.Laínz﻿ in Anales Jard. Bot. Madrid 43: 473. 1986 [“1987”] syn. sec. Bernal et al. (1990) ≡ Dianthusmulticepssubsp.multiaffinis (Pau) Rivas Mart.﻿ in Itinera Geobot. 15: 700. 2002 syn. sec. WFO 2018

***Dianthusmulticeps* Costa ex Willk.﻿ in Linnaea 30: 88. 1859.** Sec. WFO 2018

≡ Dianthuscintranussubsp.multiceps (Costa ex Willk.) Tutin﻿ in Feddes Repert. Spec. Nov. Regni Veg. 68: 190. 1963 syn. sec. Marhold (2011) ≡ Dianthuspungenssubsp.multiceps (Costa ex Willk.) O.Bolòs & Vigo﻿ in Butl. Inst. Catalana Hist. Nat., Secc. Bot. 38(1): 88. 1974 syn. sec. Marhold (2011) ≡ Dianthuspungensvar.multiceps (Costa ex Willk.) O.Bolòs & Vigo﻿ in Butl. Inst. Catalana Hist. Nat., Secc. Bot. 38: 88. 1974 syn. sec. Kew WCVP (2019)

= Dianthus×bergadensis Sennen﻿ in Bol. Soc. Ibér. Ci. Nat. 25: 144. 1926 syn. sec. Bernal et al. (1990)

= Dianthus×corberae Sennen﻿ in Bol. Soc. Ibér. Ci. Nat. 25: 143. 1926 [“1927”] syn. sec. Bernal et al. (1990)

= Dianthus×notabilis Sennen﻿ in Bol. Soc. Ibér. Ci. Nat. 25: 207. 1926 [“1927”] syn. sec. Bernal et al. (1990)

**Dianthusmulticepssubsp.multiceps**﻿

**Dianthusmulticepssubsp.praepyrenaicus M.Bernal﻿ in Anales Jard. Bot. Madrid 44: 569. 1987.** Sec. Bernal et al. (1990)

***Dianthusmultiflorus* Deniz & Aykurt﻿ in Phytokeys 63: 3. 2016.** Sec. Deniz et al. (2016)

***Dianthusmultisquamatus* F.N.Williams﻿ in J. Bot. 23: 344. 1885.** Sec. Williams (1893)

***Dianthusmultisquameus* Bondarenko & R.M.Vinogr.﻿, Opred. Rast. Sred. Azii 2: 327. 1971.** Sec. Czerepanov (1995)

***Dianthusmuschianus* Kotschy ex Boiss.﻿, Fl. Orient. 1: 510. 1867.** Sec. Marhold (2011)

***Dianthusmyrtinervius* Griseb.﻿, Spic. Fl. Rumel. 1: 194. 1843.** Sec. Dimopoulos et al. (2013)

= Dianthusmyrtinerviussubsp.zupancicii Micevski & E.Mayer﻿ in Razpr. Slov. Akad. Znan. Umetn., Razr. Nar. Vede 43: 411. 2002 syn. sec. WFO 2018

**Dianthusmyrtinerviussubsp.caespitosus Strid & Papan. in Ann. Mus. Goulandris 4: 219. 1978.** Sec. Dimopoulos et al. (2013)

= Dianthusmyrtinerviusvar.oxylepis Boiss.﻿, Fl. Orient. 1: 509. 1867 syn. sec. this publication ≡ *Dianthusoxylepis* (Boiss.) Kümmerle & Jáv.﻿, Balkán-Kutat Tud. Eredm. 3: 233. 1926 syn. sec. Dimopoulos et al. (2013)

= *Dianthuskajmaktzalanicus* Mitsevski﻿ in Fragm. Balcan. Mus. Macedon. Sci. Nat. 10(4): 29. 1977 syn. sec. Dimopoulos et al. (2013)

**Dianthusmyrtinerviussubsp.myrtinervius**﻿

***Dianthusnamaensis* Schinz﻿ in Bull. Herb. Boiss. 5(App. 3): 84. 1897.** Sec. African Plant Database (version 3.4.0)

= *Dianthuspearsonii* Burtt Davy﻿ in Bull. Misc. Inform. Kew 1922: 215. 1922 syn. sec. African Plant Database (version 3.4.0)

**Dianthusnamaensisvar.dinteri (Schinz) S.S.Hooper﻿.** Sec. African Plant Database (version 3.4.0)

= *Dianthusdinteri* Schinz﻿ in Vierteljahrsschr. Naturf. Ges. Zürich 74: 110. 1929 syn. sec. African Plant Database (version 3.4.0)

**Dianthusnamaensisvar.junceus (Burtt Davy) S.S.Hooper in Hookers Icon. Pl. 37: 30. 1959.** Sec. African Plant Database (version 3.4.0)

≡ *Dianthusjunceus* Burtt Davy﻿ in Bull. Misc. Inform. Kew 1922: 219. 1922 syn. sec. African Plant Database (version 3.4.0)

**Dianthusnamaensisvar.namaensis**﻿

***Dianthusnangarharicus* Rech.f.﻿ in Plant Syst. Evol. 142: 240. 1983.** Sec. Rechinger (1988)

***Dianthusnanshanicus* Chang Y.Yang & L.X.Dong﻿ in Bull. Bot. Res., Harbin 28: 644. 2008.** Sec. Dequan & Turland (2001)

***Dianthusnardiformis*Janka﻿ in Oesterr. Bot. Z. 23: 195. 1873.** Sec. Marhold (2011)

***Dianthusnihatii* Güner﻿, Fl. Turkey 11: 318. 2000 [“2001”].** Sec. Güner (2000)

***Dianthusnitidus* Waldst. & Kit.﻿ in Descr. Icon. Pl. Hung. 2: 209. 1805.** Sec. Marhold (2011)

***Dianthusnoeanus* Boiss.﻿, Diagn. Pl. Orient., ser. 2, 5: 52. 1856.** Sec. Dimopoulos et al. (2013)

≡ Dianthusstrictussubsp.noeanus (Boiss.) Stoj. & Acht.﻿ in Sborn. Bulg. Akad. Nauk. 29: 81. 1935 syn. sec. Dimopoulos et al. (2013) ≡ Dianthuspetraeussubsp.noeanus (Boiss.) Tutin﻿ in Feddes Repert. Spec. Nov. Regni Veg. 68: 190. 1963 syn. sec. WFO 2018

***Dianthusnudiflorus* Griff.﻿, Not. Pl. Asiat. 4: 466. 1854.** Sec. Madhani et al. (2018)

= *Veleziarigida* L.﻿, Sp. Pl.: 332. 1753 syn. sec. Kew WCVP (2019)

= Veleziarigidavar.glabrata Regel﻿ in Izv. Imp. Obshch. Lyubit. Estestv. Moskovsk. Univ. 34(2): 13. 1882 syn. sec. POWO. Plants of the World Online. Facilitated by the Royal Botanic Gardens, Kew.

= Veleziarigidavar.sessiliflora F.N.Williams﻿ in J. Bot. 37: 28. 1899 syn. sec. Kew WCVP (2019)

***Dianthusoliastrae* Bacch., Brullo, Casti & Giusso﻿ in Nordic J. Bot. 28: 171. 2010.** Sec. Bacchetta et al. (2010)

***Dianthusorientalis* Adams﻿ in Beitr. Naturk. 1: 54. 1805.** Sec. Kuzmina & Nersesyan (2012)

= *Dianthusfimbriatus* M.Bieb.﻿, Fl. Taur.-Caucas. 1: 332. 1808 syn. sec. Kuzmina & Nersesyan (2012)

= *Dianthuspogonopetalus* Boiss. & Kotschy﻿ in Boissier, Diagn. Pl. Orient. ser. 2, 6: 29. 1859 syn. sec. Kuzmina & Nersesyan (2012)

**Dianthusorientalissubsp.aphanoneurus Rech.f. in Plant Syst. Evol. 151: 290. 1986.** Sec. Kuzmina & Nersesyan (2012)

≡ *Dianthusaphanoneurus* (Rech.f.) M.Kuzmina﻿ in Bot. Zhurn. (Moscow & Leningrad) 81: 82. 1996 syn. sec. Kuzmina & Nersesyan (2012)

**Dianthusorientalissubsp.gilanicus Rech.f.﻿ in Pl. Syst. Evol. 151(3–4): 289. 1986.** Sec. Rechinger (1988)

**Dianthusorientalissubsp.gorganicus Rech.f.﻿ in Pl. Syst. Evol. 151(3–4): 291. 1986.** Sec. Rechinger (1988)

**Dianthusorientalissubsp.ketzkhovelii (Makaschv.) Nersesian, Konspekt Fl. Kavkaza 3(2): 194. 2012.** Sec. Kuzmina & Nersesyan (2012)

≡ *Dianthusketzkhovelii* Makaschv.﻿ in Soobshch. Akad. Nauk Gruzinsk. S.S.R. 8(7): 447. 1947 syn. sec. Kuzmina & Nersesyan (2012)

**Dianthusorientalissubsp.macropetalus (Boiss.) Rech.f.﻿ in Plant Syst. Evol. 151: 289. 1986.** Sec. Kuzmina & Nersesyan (2012)

≡ Dianthusfimbriatusvar.macropetalus Boiss.﻿, Fl. Orient. Suppl.: 77. 1888 syn. sec. Kuzmina & Nersesyan (2012)

**Dianthusorientalissubsp.nassireddinii (Stapf) Rech.f.﻿ in Plant Syst. Evol. 151: 292. 1986.** Sec. Kuzmina & Nersesyan (2012)

≡ *Dianthusnassireddinii* Stapf﻿ in Denkschr. Kaiserl. Akad. Wiss., Wien. Math.-Naturwiss. Kl. 51: 279. 1886 syn. sec. WFO 2018

= *Dianthusdumulosus* Boiss. & A.Huet﻿ in Boissier, Diagn. Pl. Orient., ser. 2, 5: 53. 1856 syn. sec. Kuzmina & Nersesyan (2012)

= Dianthusfimbriatusvar.brachyodontus Boiss. & A.Huet﻿ in Boissier, Diagn. Pl. Orient., ser. 2, 5: 53. 1856 syn. sec. Kuzmina & Nersesyan (2012) ≡ *Dianthusbrachyodontus* (Boiss. & A.Huet) Grossh.﻿, Fl. Cauc., ed. 2, 3: 294. 1945 syn. sec. Kuzmina & Nersesyan (2012)

**Dianthusorientalissubsp.obtusisquameus (Boiss.) Rech.f.﻿ in Plant Syst. Evol. 151: 291. 1986.** Sec. Kuzmina & Nersesyan (2012)

≡ Dianthusfimbriatusvar.obtusisquameus Boiss.﻿, Fl. Orient. 1: 495. 1867 syn. sec. Kuzmina & Nersesyan (2012)

**Dianthusorientalissubsp.orientalis**﻿

**Dianthusorientalissubsp.scoparius (Fenzl ex Boiss.) Bornm.﻿ in Beih. Bot. Centralbl. 28(2): 134. 1911.** Sec. Rechinger (1988)

≡ *Dianthusscoparius* Fenzl ex Boiss.﻿, Fl. Orient. 1: 494. 1867 syn. sec. Kuzmina & Nersesyan (2012)

= *Dianthusfallax* Rech.f. & Esfand.﻿ in Bot. Jahrb. Syst. 75: 362. 1951 syn. sec. Rechinger (1988)

**Dianthusorientalissubsp.stenocalyx (Boiss.) Rech.f.﻿ in Plant Syst. Evol. 151: 292. 1986.** Sec. Rechinger (1988)

≡ Dianthusfimbriatusvar.stenocalyx Boiss.﻿, Fl. Orient. 1: 495. 1867 syn. sec. Rechinger (1988)

= *Dianthusmacronyx* Fenzl ex Boiss.﻿, Fl. Orient. 1: 495. 1867 syn. sec. Rechinger (1988)

= *Dianthuspulverulentus* Stapf﻿ in Denkschr. Kaiserl. Akad. Wiss., Wien. Math.-Naturwiss. Kl. 51: 279. 1886 syn. sec. Rechinger (1988)

***Dianthusoschtenicus* Galushko﻿ in Novosti Sist. Vyssh. Rast. 2: 118. 1965.** Sec. Kuzmina & Nersesyan (2012)

***Dianthuspachygonus* (Fisch. & C.A.Mey.) Fassou, N.Korotkova, Nersesian & Borsch﻿.** Sec. this publication 167

≡ *Tunicapachygona* Fisch. & C.A.Mey.﻿, Index Sem. Hort. Bot. Petropol. 4: 50. 1838 syn. sec. this publication;

= *Saponariacretica* L.﻿, Sp. Pl., ed. 2 1: 584. 1762 syn. sec. this publication ≡ *Tunicacretica* (L.) Fisch. & C.A.Mey.﻿, Index Sem. Hort. Bot. Petropol. 4: 49. 1837 syn. sec. Kew WCVP (2019) ≡ *Petrorhagiacretica* (L.) P.W.Ball & Heywood﻿ in Bull. Brit. Mus. (Bot.) 3(4): 142. 1964 ≡ *Fiedleriacretica* (L.) Ovcz.﻿, Fl. Tadzhiksk. S.S.R. 3: 608. 1968 syn. sec. this publication

***Dianthuspaghmanicus* Rech.f.﻿ in Bot. Jahrb. Syst. 75: 362. 1951.** Sec. Rechinger (1988)

***Dianthuspalinensis* S.S.Ying﻿, Col. Ill. Fl. Taiwan 2: 693. 1987.** Sec. Dequan & Turland (2001)

***Dianthuspallidiflorus* Ser.﻿, Prodr. 1: 358. 1824.** Sec. Marhold (2011)

≡ Dianthuscampestrissubsp.pallidiflorus (Ser.) Schmalh.﻿, Fl. Sredn. Yuzhn. Rossii 1: 447. 1895 syn. sec. Marhold (2011)

= *Dianthuspaniculatus* Gueldenst.﻿, Reis. Russland 1: 68. 1787 syn. sec. WFO 2018

= *Dianthuspallens* M.Bieb.﻿, Fl. Taur.-Caucas. 1: 325. 1808 syn. sec. WFO 2018

= *Dianthussaxatilis* Pall. ex M.Bieb.﻿, Fl. Taur.-Caucas. 1: 329. 1808 syn. sec. WFO 2018

= *Dianthusemarginatus* Ser.﻿, Prodr. 1: 359. 1824 syn. sec. WFO 2018

= *Dianthusparviflorus* Willd. ex Ledeb.﻿, Fl. Ross. 1: 279. 1842 syn. sec. WFO 2018

= *Dianthusaridus* Griseb. ex Janka﻿ in Oesterr. Bot. Z. 23: 196. 1873 syn. sec. Marhold (2011)

= *Dianthusmaeoticus* Klokov﻿ in Sc. Mag. Biol. 1927: 13. 1927 syn. sec. Marhold (2011)

***Dianthuspamiralaicus* Lincz.﻿ in Novosti Sist. Vyssh. Rast. 1: 76. 1964.** Sec. Czerepanov (1995)

***Dianthuspancicii* Velen.﻿ in Sitzungsber. Königl. Böhm. Ges. Wiss., Math.-Naturwiss. Cl. 1886(Extr.): 9. 1886.** Sec. Marhold (2011)

≡ Dianthusstenopetalusvar.pancicii (Velen.) F.N.Williams﻿ in J. Linn. Soc., Bot. 29: 389. 1893 syn. sec. this publication ≡ Dianthuscruentusvar.pancicii (Velen.) Stoj. & Acht.﻿, Krit. Stud. Nelk. Bulg.: 56. 1935 syn. sec. this publication ≡ Dianthuscruentussubsp.pancicii (Velen.) Stoj. & Stef.﻿, Fl. Bulg., ed. 3: 405. 1948 syn. sec. Marhold (2011)

= *Dianthustristis* Velen.﻿ in Sitzungsber. Königl. Böhm. Ges. Wiss., Math.-Naturwiss. Cl. 1890(2): 41. 1890 syn. sec. Marhold (2011)

**Dianthus×paradoxus Rouy & Foucaud﻿, Fl. France 3: 187. 1896.** Sec. WFO 2018

***Dianthuspatentisquameus* Bondarenko & R.M.Vinogr.﻿, Opred. Rast. Sred. Azii 2: 327. 1971.** Sec. Czerepanov (1995)

***Dianthuspavlovii* Lazkov﻿ in Bot. Zhurn. (Moscow & Leningrad) 87(12): 113. 2002.** Sec. Lazkov (2006)

– *Dianthusattenuatus* Pavlov﻿, Fl. Kazakhst. 3: 426. 1960, nom. inval. syn. sec. Lazkov (2002)

***Dianthuspavonius* Tausch﻿ in Flora 22: 145. 1839.** Sec. Marhold (2011)

= *Dianthusneglectus* Loisel.﻿ in J. Bot. (Paris) 2: 321. 1809 syn. sec. Marhold (2011) ≡ *Cylichnanthusneglectus* (Loisel.) Dulac﻿, Fl. Hautes-Pyrénées: 262. 1867

***Dianthuspelviformis* Heuff.﻿ in Flora 36: 625. 1853.** Sec. Marhold (2011)

= *Dianthusbulgaricus* Velen.﻿, Fl. Bulg.: 78. 1891 syn. sec. Marhold (2011)

= *Dianthuszernyi* Hayek﻿ in Repert. Spec. Nov. Regni Veg. Beih. 30(1): 237. 1924 syn. sec. Marhold (2011)

***Dianthuspendulus* Boiss. & Blanche﻿ in Boissier, Diagn. Pl. Orient. ser. 2, 6: 28. 1859.** Sec. Rechinger (1988)

***Dianthuspersicus* Hausskn.﻿ in Mitt. Geogr. Ges. (Thüringen) Jena 9: 16. 1891.** Sec. Rechinger (1988)

***Dianthuspetraeus* Waldst. & Kit.﻿ in Descr. Icon. Pl. Hung. 3: 246. 1807.** Sec. Dimopoulos et al. (2013)

= *Dianthusbohemicus* Mayer ex Tausch﻿ in Flora 13: 246. 1830 syn. sec. WFO 2018

= *Dianthusbebius* Vis. ex Rchb.﻿ in Icon. Fl. Germ. Helv. 6: 47. 1844 syn. sec. WFO 2018

= *Dianthusintegripetalus* Schur﻿, Enum. Pl. Transsilv.: 98. 1866 syn. sec. WFO 2018

= *Dianthuspseudocaesius* Schur﻿, Enum. Pl. Transsilv.: 98. 1866 syn. sec. WFO 2018

= *Dianthusliliodorus* Pančić﻿, Fl. Serbiae: 176. 1874 syn. sec. Marhold (2011) ≡ Dianthuspetraeusf.liliodorus (Pančić) Hayek﻿ in Repert. Spec. Nov. Regni Veg. Beih. 30(1): 252. 1924 syn. sec. Kew WCVP (2019)

= *Dianthuskitaibelii*Janka ex Beck﻿ in Ann. K. K. Naturhist. Hofmus. 2: 192. 1889 syn. sec. WFO 2018

= *Dianthusskorpilii* Velen.﻿ in Sitzungsber. Königl. Böhm. Ges. Wiss., Math.-Naturwiss. Cl. 1890(2): 40. 1890 syn. sec. WFO 2018

**Dianthuspetraeussubsp.orbelicus (Velen.) Greuter & Burdet in Willdenowia 12: 187. 1982.** Sec. Dimopoulos et al. (2013)

≡ Dianthusstrictussubsp.orbelicus Velen.﻿, Fl. Bulg. Suppl. 1: 40. 1898 syn. sec. Marhold (2011)

= *Dianthusstrictus* Sm.﻿, Fl. Graec. Prodr. 1(2): 288. 1809 syn. sec. Marhold (2011)

= *Dianthussimonkaianus* Péterfi﻿ in Magyar Bot. Lapok 15: 14. 1916 syn. sec. Marhold (2011) ≡ Dianthuspetraeussubsp.simonkaianus (Péterfi) Tutin﻿ in Feddes Repert. Spec. Nov. Regni Veg. 68: 190. 1963 syn. sec. Marhold (2011)

= *Dianthussuendermannii* Bornm.﻿ in Repert. Spec. Nov. Regni Veg. 17: 40. 1921 syn. sec. WFO 2018

= *Dianthusstefanoffii* Eig﻿ in J. Bot. 75: 191. 1937 syn. sec. Dimopoulos et al. (2013) ≡ Dianthuspetraeussubsp.stefanoffii (Eig) Greuter & Burdet﻿ in Willdenowia 12: 187. 1982 syn. sec. Dimopoulos et al. (2013)

**Dianthuspetraeussubsp.petraeus**﻿

***Dianthuspinifolius* Sm.﻿, Fl. Graec. Prodr. 1(2): 284. 1809.** Sec. Marhold (2011)

= *Dianthusbrevifolius* Friv.﻿ in Flora 18: 334. 1835 syn. sec. WFO 2018 ≡ Dianthuspinifoliussubsp.brevifolius (Friv.) Stoj. & Stef.﻿, Fl. Bulg., ed. 3: 406. 1948 syn. sec. Marhold (2011)

= *Dianthusrumelicus* Velen.﻿, Fl. Bulg.: 78. 1891 syn. sec. Marhold (2011) ≡ Dianthuspinifoliussubsp.rumelicus (Velen.) Stoj. & Acht.﻿, Sborn. Bălg. Akad. Nauk. 29(2): 66. 1935 syn. sec. Marhold (2011)

= Dianthuspinifoliussubsp.smithii Wettst.﻿ in Biblioth. Bot. 26: 33. 1892 syn. sec. Marhold (2011)

= *Dianthusserresianus* Halácsy & Charrel﻿ in Oesterr. Bot. Z. 42: 271. 1892 syn. sec. Marhold (2011)

= *Dianthussmithii* Wettst.﻿ in Biblioth. Bot. 26: 33. 1892 syn. sec. this publication

= *Dianthusrhodopeus* Davidov﻿ in Trav. Soc. Bulg. Sci. Nat. 8: 55. 1915, nom. illeg. syn. sec. Kew WCVP (2019)

= *Dianthusserresianus* Hayek﻿ in Denkschr. Kaiserl. Akad. Wiss., Wien. Math.-Naturwiss. Kl. 94: 140. 1917 syn. sec. Marhold (2011)

= *Dianthusserulis* Kulcz.﻿ in Rozpr. Wydz. Mat.-Przyr Polsk Akad. Umiejetn., Dzial A/B, Nauki Mat-Fiz. Biol. 59: 361. 1923 syn. sec. Marhold (2011) ≡ Dianthuspinifoliussubsp.serulis (Kulcz.) Trinajstić in Suppl. Fl. Anal. Jugosl. 6: 9. 1979 syn. sec. Marhold (2011) – Dianthuspinifoliussubsp.serulis (Kulcz.) Trinajstić in Suppl. Fl. Anal. Jugosl. 5: 753. 1979, nom. inval. syn. sec. this publication

**Dianthuspinifoliussubsp.lilacinus (Boiss. & Heldr.) Wettst.﻿ in Biblioth. Bot. 26: 33. 1892.** Sec. Marhold (2011)

≡ *Dianthuslilacinus* Boiss. & Heldr.﻿ in Boissier, Diagn. Pl. Orient., ser. 2, 1: 63. 1854 syn. sec. Marhold (2011)

**Dianthuspinifoliussubsp.pinifolius**﻿

**Dianthuspinifoliussubsp.serbicus Wettst.﻿ in Biblioth. Bot. 26: 34. 1892.** Sec. Marhold (2011)

≡ *Dianthusserbicus* (Wettst.) Hayek﻿ in Kaiserl. Akad. Wiss. Wien, Math.-Naturwiss. Kl., Denkschr. 94: 141. 1918 syn. sec. this publication

**Dianthuspinifoliussubsp.tenuicaulis (Turrill) Strid﻿, Fl. Hellenica 1: 368. 1997.** Sec. Marhold (2011)

≡ *Dianthustenuicaulis* Turrill﻿ in Bull. Misc. Inform. Kew 1929: 224. 1929 syn. sec. Marhold (2011)

***Dianthusplumarius* L.﻿, Sp. Pl.: 411. 1753.** Sec. Marhold (2011)

≡ *Tunicaplumaria* (L.) Scop.﻿, Fl. Carniol., ed. 2, 1: 300. 1771 syn. sec. POWO. Plants of the World Online. Facilitated by the Royal Botanic Gardens, Kew.; ≡ *Caryophyllusplumarius* (L.) Moench﻿, Methodus: 59. 1794 syn. sec. POWO. Plants of the World Online. Facilitated by the Royal Botanic Gardens, Kew. ≡ *Cylichnanthusplumarius* (L.) Dulac﻿, Fl. Hautes-Pyrénées: 262. 1867 syn. sec. POWO. Plants of the World Online. Facilitated by the Royal Botanic Gardens, Kew. ≡ *Sileneplumaria* (L.) E.H.L.Krause﻿, Deutschl. Fl. Abbild., ed. 2, 5: 114. 1901 syn. sec. Kew WCVP (2019);

= *Dianthushortensis* Schrad. ex Willd.﻿, Enum. Pl.: 469. 1809 syn. sec. WFO 2018 ≡ Dianthusplumariusvar.hortensis (Schrad. ex Willd.) Trevir.﻿, Index Seminum (WROCL, Wratislaviensi) 1818: 3. 1818 syn. sec. Kew WCVP (2019)

= *Dianthusportensis* Libosch. ex Ser.﻿, Prodr. 1: 363. 1824 syn. sec. WFO 2018 ≡ Dianthusplumariusvar.portensis Ser.﻿, Prodr. 1: 363. 1824 syn. sec. Kew WCVP (2019)

= Dianthusplumariusvar.blandus Rchb.﻿, Fl. Germ. Excurs.: 807. 1832 syn. sec. Marhold (2011) ≡ *Dianthusblandus* (Rchb.) Hayek﻿, Fl. Steiermark 1: 320. 1908 syn. sec. Marhold (2011)

= *Dianthusodoratus* Vest ex Steud.﻿, Nomencl. Bot., ed. 2, 1: 500. 1840 syn. sec. WFO 2018

= *Plumariavulgaris* Opiz﻿, Seznam: 75. 1852 syn. sec. POWO. Plants of the World Online. Facilitated by the Royal Botanic Gardens, Kew.

= Dianthusplumariusvar.parviflorus Kauffm.﻿, Index Seminum (MHA, Mosquensis) 1868: 9. 1868 syn. sec. Kew WCVP (2019)

= *Dianthushoppei* Port. ex Hayek﻿, Fl. Steiermark 1: 320. 1908 syn. sec. Marhold (2011)

= *Dianthusneilreichii* Hayek﻿ syn. sec. Marhold (2011)

= *Dianthusdubius* Hornem.﻿ ex DC.﻿, Cat. Pl. Horti Monsp.: 103. 1813, nom. illeg. syn. sec. Marhold (2011) – *Dianthusdubius* Hornem. in Hort. Bot. Hafn. 1: 408. 1813 syn. sec. WFO 2018 [is later isonym of *Dianthusdubius* Hornem. ex DC.]

**Dianthusplumariussubsp.plumarius**﻿

**Dianthusplumariussubsp.regis-stephani (Rapaics) Baksay﻿ in Bot. Közlem. 57: 215. 1970.** Sec. Marhold (2011)

≡ *Dianthusregis-stephani* Rapaics﻿ syn. sec. WFO 2018 ≡ Dianthushungaricussubsp.regis-stephani (Rapaics) Holub﻿ in Folia Geobot. Phytotax. 9: 273. 1974 syn. sec. WFO 2018

***Dianthusplumbeus* Schischk.﻿ in Izv. Tomsk. Gosud. Univ. 81: 451. 1928.** Sec. Marhold (2011)

***Dianthuspolylepis* Bien. ex Boiss.﻿, Fl. Orient. 1: 497. 1867.** Sec. Rechinger (1988)

**Dianthuspolylepissubsp.binaludensis (Rech.f.) Vaezi & Behrooz.﻿ in Pl. Syst. Evol. 299: 1430. 2013.** Sec. Farsi et al. (2013)

≡ *Dianthusbinaludensis* Rech.f.﻿ in Plant Syst. Evol. 142: 242. 1983 syn. sec. Farsi et al. (2013)

**Dianthuspolylepissubsp.polylepis**﻿

***Dianthuspolymorphus* M.Bieb.﻿, Fl. Taur.-Caucas. 1: 324. 1808.** Sec. Kuzmina & Nersesyan (2012)

= *Dianthusdichotomus* Pall.﻿, Reise Südl. Statthaltersch. Russ. Reich. 2: 335. 1801 syn. sec. WFO 2018

= *Dianthusdiutinus* Kit. ex Schult.﻿, Oestr. Fl. ed. 2, 1: 655. 1814 syn. sec. WFO 2018 ≡ Dianthuspolymorphusvar.diutinus (Kit.) Ser.﻿, Prodr. 1: 356. 1824 syn. sec. this publication

= *Dianthusatratus* Beaupré ex Ser.﻿, Prodr. 1: 356. 1824 syn. sec. WFO 2018

= *Dianthusponticus* Wahlenb.﻿, Isis (Oken) 21: 985. 1828 syn. sec. WFO 2018

= *Dianthusglomeratus* Pall. ex Ledeb.﻿, Fl. Ross. 1: 276. 1842 syn. sec. WFO 2018

= *Dianthusintermedius* Willd. ex Ledeb.﻿, Fl. Ross. 1: 276. 1842 syn. sec. WFO 2018

= *Dianthusautumnalis* Kit.﻿ in Linnaea 32: 530. 1863 syn. sec. WFO 2018

= *Dianthussabuli* Kit.﻿ in Linnaea 32: 530. 1863 syn. sec. WFO 2018

= *Dianthusplatyodon* Klokov﻿ in Bot. Zhurn. (Kiev) 5: 27. 1948 syn. sec. WFO 2018

= Dianthuspolymorphusvar.platyodon Sanda﻿ syn. sec. WFO 2018

***Dianthuspraecox* Willd. ex Spreng.﻿, Syst. Veg., ed. 16, 2: 381. 1825.** Sec. Marhold (2011)

≡ Dianthusplumariussubsp.praecox (Willd. ex Spreng.) Domin﻿ syn. sec. Marhold (2011)

= *Dianthushungaricus* Pers.﻿, Syn. Pl. 1: 494. 1805 syn. sec. WFO 2018

**Dianthuspraecoxsubsp.lumnitzeri (Wiesb.) Kmet’ová﻿, Biol. Práce Slov. Akad. Vied. 5: 63. 1985.** Sec. Marhold (2011)

≡ *Dianthuslumnitzeri* Wiesb.﻿ in Bot. Centralbl. 26: 85. 1886 syn. sec. Marhold (2011) ≡ Dianthushungaricussubsp.lumnitzeri (Wiesb.) Holub﻿ in Folia Geobot. Phytotax. 9: 272. 1974 syn. sec. WFO 2018 ≡ Dianthusplumariussubsp.lumnitzeri (Wiesb.) Domin﻿ syn. sec. Marhold (2011)

= Dianthusplumariusf.palaviensis Novák﻿ syn. sec. WFO 2018 ≡ Dianthuslumnitzerisubsp.palaviensis (Novák) Dostál﻿ in Folia Mus. Rerum Nat. Bohemiae Occid., Bot. 21: 5. 1984 syn. sec. WFO 2018

**Dianthuspraecoxsubsp.praecox**﻿

**Dianthuspraecoxsubsp.pseudopraecox (Novák) Kmet’ová﻿, Biol. Práce Slov. Akad. Vied. 5: 69. 1985.** Sec. Marhold (2011)

≡ Dianthusplumariusf.pseudopraecox Novák﻿ syn. sec. Marhold (2011) ≡ Dianthuslumnitzerisubsp.pseudopraecox (Novák) Dostál﻿ in Folia Mus. Rerum Nat. Bohemiae Occid., Bot. 21: 4. 1984 syn. sec. Marhold (2011)

***Dianthuspratensis* M.Bieb.﻿, Fl. Taur.-Caucas. 3: 300. 1819.** Sec. Marhold (2011)

≡ *Dianthusseguieri* lusus *pratensis* (M.Bieb.) Regel﻿ in Bull. Soc. Imp. Naturalistes Moscou 34(2): 526. 1862 syn. sec. Kew WCVP (2019)

= *Dianthuschloroleucus* Fisch.﻿ in Hort. Bot. Hafn. Suppl: 137. 1819 syn. sec. WFO 2018

= *Dianthusseguieri* lusus *angustifolius* Regel﻿ in Bull. Soc. Imp. Naturalistes Moscou 34(2): 526. 1862 syn. sec. Kew WCVP (2019)

= *Dianthusseguieri* lusus *humilis* Regel﻿ in Bull. Soc. Imp. Naturalistes Moscou 34(2): 526. 1862 syn. sec. Kew WCVP (2019)

**Dianthuspratensissubsp.pratensis**﻿

**Dianthuspratensissubsp.racovitzae (Prodán) Tutin﻿ in Feddes Repert. Spec. Nov. Regni Veg. 68: 189. 1963.** Sec. Marhold (2011)

≡ *Dianthusracovitzae* Prodán﻿, Bul. Soc. St. Cluj 10(2): 162. 1948 syn. sec. Marhold (2011)

***Dianthuspseudarmeria* M.Bieb.﻿, Fl. Taur.-Caucas. 1: 323. 1808.** Sec. Kuzmina & Nersesyan (2012)

***Dianthuspseudobarbatus* Besser ex Ledeb.**﻿ Sec. Czerepanov (1995)

= Dianthustrifasciculatussubsp.euponticus Kleopow﻿ syn. sec. Czerepanov (1995)

***Dianthuspseudocrinitus* Behrooz. & Joharchi﻿ in Phytotaxa 156: 69. 2014.** Sec. Vaezi et al. (2014)

***Dianthuspseudorigidus* (Hub.-Mor.) Fassou, N.Korotkova, Dimop. & Borsch﻿.** Sec. this publication 173

≡ *Veleziapseudorigida* Hub.-Mor.﻿ in Bauhinia 2: 195. 1963 syn. sec. this publication

***Dianthuspungens* L.﻿, Mant. Pl. 2: 240. 1771.** Sec. Bernal et al. (1990)

= *Dianthuspurpureus* Poir.﻿, Encycl. 4: 523. 1798 syn. sec. WFO 2018

= *Dianthusserratus* Lapeyr.﻿, Hist. Pl. Pyrénées: 241. 1813 syn. sec. Marhold (2011) ≡ Dianthusaspervar.serratus (Lapeyr.) Ser.﻿, Prodr. 1: 357. 1824 syn. sec. Kew WCVP (2019)

= *Dianthusinsignitus* Timb.-Lagr.﻿ in Bull. Soc. Bot. France 11: 143. 1864 syn. sec. WFO 2018

**Dianthuspungenssubsp.brachyanthus (Boiss.) B.Fern.Casas, G.López & M.Laínz﻿ in Anales Jard. Bot. Madrid 44: 180. 1987.** Sec. Bernal et al. (1990)

≡ *Dianthusbrachyanthus* Boiss.﻿, Fl. Orient. 1: 701. 1867 syn. sec. Bernal et al. (1990) ≡ Dianthussubacaulissubsp.brachyanthus (Boiss.) P.Fourn.﻿, Quatre Fl. France: 331. 1936 syn. sec. Bernal et al. (1990) ≡ Dianthusstrictusvar.brachyanthus (Boiss.) Boiss.﻿ syn. sec. this publication

= *Dianthusattenuatus* Xatard ex Walp.﻿, Repert. Bot. Syst. 1: 267. 1842 syn. sec. WFO 2018

= *Dianthuspungens* J.Gay ex Boiss.﻿, Fl. Orient. 1: 85. 1867 syn. sec. WFO 2018

= Dianthusbrachyanthusvar.maroccanus Pau & Font Quer﻿, Iter Marocc. 1927: 197. 1928 syn. sec. WFO 2018 ≡ Dianthussubacaulisvar.maroccanus (Pau & Font Quer) Maire﻿, Fl. Afrique N. 10: 314. 1963 syn. sec. Kew WCVP (2019)

= Dianthusbrachyanthussubsp.cantabricus Font Quer﻿ in Collect. Bot. (Barcelona) 3: 355. 1953 syn. sec. Marhold (2011) ≡ Dianthussubacaulissubsp.cantabricus (Font Quer) Laínz﻿ in Bol. Inst. Estud. Adsturianos, Supl. Ci. 15: 12. 1970 syn. sec. Marhold (2011)

= Dianthusbrachyanthusvar.nivalis Willk.﻿ syn. sec. WFO 2018 ≡ Dianthussubacaulissubsp.nivalis (Willk.) Malag.﻿, Subesp. Variac. Geogr.: 6. 1973 syn. sec. WFO 2018

**Dianthuspungenssubsp.hispanicus (Asso) O.Bolòs & Vigo﻿ in Butl. Inst. Catalana Hist. Nat., Secc. Bot. 38(1): 188. 1974.** Sec. Bernal et al. (1990)

≡ *Dianthushispanicus* Asso﻿, Syn. Stirp. Aragon.: 53. 1779 syn. sec. Bernal et al. (1990) ≡ Dianthuspungensvar.hispanicus (Asso) Ser.﻿, Prodr. 1: 360. 1824 syn. sec. this publication

= Dianthusbrachyanthussubsp.tarraconensis (Costa) Rivas Mart. ex M.B.Crespo & Mateo﻿ in Flora Montiber. 45: 90. 2010 syn. sec. Bernal et al. (1990) ≡ Dianthusbrachyanthusvar.tarraconensis Costa﻿ in Anales Soc. Esp. Hist. Nat. 3: 183. 1874 syn. sec. Bernal et al. (1990) ≡ Dianthuspungenssubsp.tarraconensis (Costa) O.Bolòs & Vigo﻿ in Butl. Inst. Catalana Hist. Nat., Secc. Bot. 38(1): 88. 1974 syn. sec. Bernal et al. (1990) ≡ Dianthushispanicussubsp.tarraconensis (Costa) Molero﻿ in Folia Bot. Misc. 3: 12. 1982 syn. sec. Bernal et al. (1990)

**Dianthuspungenssubsp.pungens**﻿

**Dianthuspungenssubsp.ruscinonensis (Boiss.) M.Bernal, Laínz & Muñoz Garm.﻿ in Anales Jard. Bot. Madrid 44: 571. 1987.** Sec. Bernal et al. (1990)

≡ Dianthusbrachyanthusvar.ruscinonensis Boiss.﻿, Fl. Orient. 1: 86. 1867 syn. sec. Bernal et al. (1990) ≡ *Dianthusruscinonensis* (Boiss.) Sennen﻿ in Trab. Mus. Ci. Nat., Ser. Bot., 15: 48. 1931 syn. sec. Bernal et al. (1990) ≡ Dianthussubacaulissubsp.ruscinonensis (Boiss.) G.Bosc & Kerguélen﻿ in Lejeunia 120: 80. 1987 syn. sec. Bernal et al. (1990)

***Dianthuspurpureimaculatus* Podlech﻿ in Mitt. Bot. Staatssamml. München 16: 544. 1980.** Sec. Rechinger (1988)

***Dianthuspygmaeus* Hayata﻿ in Icon. Pl. Formosan. 3: 34. 1913.** Sec. Dequan & Turland (2001)

= Dianthuspygmaeusvar.albiflorus S.S.Ying﻿ in Quart. J. Chin. Forest. 8(4): 120. 1975 syn. sec. Dequan & Turland (2001) ≡ Dianthuspygmaeusf.albiflorus (S.S.Ying) S.S.Ying﻿, Fl. Taiwan ed. 2, 2: 356. 1996 syn. sec. Dequan & Turland (2001)

***Dianthuspyrenaicus* Pourr.﻿, Mém. Acad. Sci. Toulouse 3: 318. 1788.** Sec. Marhold (2011)

= *Dianthuscognobilis* (Timb.-Lagr.) Timb.-Lagr.﻿ in Bull. Soc. Bot. France 11: 143. 1864 syn. sec. WFO 2018 ≡ Dianthusrequieniivar.cognobilis Timb.-Lagr.﻿ syn. sec. WFO 2018 ≡ Dianthuspungenssubsp.cognobilis (Timb.-Lagr.) O.Bolòs & Vigo﻿ in Butl. Inst. Catalana Hist. Nat., Secc. Bot. 38(1): 88. 1974 syn. sec. WFO 2018

= *Dianthusmaritimus* (Rouy) P.Fourn.﻿, Quatre Fl. France: 332. 1936, nom. illeg. syn. sec. Marhold (2011) ≡ Dianthuspyrenaicussubsp.maritimus (Rouy) Kerguélen﻿, Coll. Patrim. Nat. 8: 13. 1993 syn. sec. WFO 2018

= Dianthuspungenssubsp.fontqueri O.Bolòs & Vigo﻿ in Butl. Inst. Catalana Hist. Nat., Secc. Bot. 38(1): 88. 1974 syn. sec. WFO 2018 ≡ Dianthushispanicussubsp.fontqueri (O.Bolòs & Vigo) A.Barber, M.B.Crespo & Mateo﻿, Contr. Coneix. Fl. Fitogeogr. Lit. Comarca Marina Alta: 77. 1999 syn. sec. WFO 2018

**Dianthuspyrenaicussubsp.attenuatus (Sm.) M.Bernal, Laínz & Muñoz Garm.﻿ in Anales Jard. Bot. Madrid 45: 364. 1988.** Sec. Bernal et al. (1990)

≡ *Dianthusattenuatus* Sm.﻿ in Trans. Linn. Soc. London 2: 301. 1794 syn. sec. WFO 2018

= *Dianthuslongiflorus* Poir.﻿, Encycl. 4: 522. 1798 syn. sec. POWO. Plants of the World Online. Facilitated by the Royal Botanic Gardens, Kew.

= Dianthusattenuatusvar.catalaunicus Willk. & Costa﻿ syn. sec. WFO 2018 ≡ *Dianthuscatalaunicus* (Willk. & Costa) Pourr. ex Willk. & Lange﻿, Prodr. Fl. Hispan. 3: 684. 1878 syn. sec. WFO 2018 ≡ Dianthuspyrenaicussubsp.catalaunicus (Willk. & Costa) Tutin﻿ in Feddes Repert. Spec. Nov. Regni Veg. 68: 190. 1963 syn. sec. WFO 2018

**Dianthuspyrenaicussubsp.pyrenaicus**﻿

***Dianthusquadridentatus* (Sm.) Fassou, N.Korotkova, Dimop. & Borsch﻿.** Sec. this publication 175

≡ *Veleziaquadridentata* Sm. in Sibthorp & Smith, Fl. Graec. Prodr. 1: 283. 1809 syn. sec. this publication

= *Veleziaclavata* d’Urv.﻿ in Mém. Soc. Linn. Paris 1: 284. 1822 syn. sec. Kew WCVP (2019)

***Dianthusraddeanus* Vierh.﻿ in Sitzungsber. Kaiserl. Akad. Wiss., Math.-Naturwiss. Cl., Abt. 1, 57(1): 1145. 1898.** Sec. Kuzmina & Nersesyan (2012)

= *Dianthustrautvetteri* Woronow﻿ in Izv. Kavkazsk. Muz. 7: 346. 1913 syn. sec. Kuzmina & Nersesyan (2012)

***Dianthusramosissimus* Pall. ex Poir.﻿, Encycl. Suppl. 4: 130. 1816.** Sec. Dequan & Turland (2001)

***Dianthusrecognitus* Schischk.﻿ in Trudy Bot. Inst. Akad. Nauk S.S.S.R., ser. 1, Fl. Sist. Vyssh. Rast. 3: 187. 1937.** Sec. Czerepanov (1995)

***Dianthusrecticaulis* Ledeb.﻿, Fl. Ross. 1(2): 287. 1842.** Sec. Mosyakin & Fedoronchuk (2018)

***Dianthusrepens* Willd.﻿, Sp. Pl. 2: 681. 1799.** Sec. Dequan & Turland (2001)

≡ Dianthusalpinusvar.repens (Willd.) Regel﻿ in Bull. Soc. Imp. Naturalistes Moscou 34(2): 531. 1862 syn. sec. Kew WCVP (2019) ≡ Dianthusalpinussubsp.repens (Willd.) Kozhevn.﻿ in Novosti Sist. Vyssh. Rast. 18: 238. 1981 syn. sec. Czerepanov (1995) ≡ Dianthuschinensissubsp.repens (Willd.) Vorosch.﻿, Florist. Issl. Razn. Rayonakh SSSR: 167. 1985 syn. sec. WFO 2018 ≡ Dianthusrepensvar.repens﻿ syn. sec. WFO 2018

= Dianthusrepensvar.scabripilosus Y.Z.Zhao﻿ in Acta Sci. Nat. Univ. Intramongol. 20: 110. 1989 syn. sec. Dequan & Turland (2001)

**Dianthusrepenssubsp.repens**﻿

**Dianthusrepenssubsp.schistosus Kuvaev﻿, Fl. Subarkt. Gor Evraz.: 144. 2006.** Sec. Kuvaev (2006)

***Dianthusrigidus* M.Bieb., Fl. Taur.-Caucas. 1: 325. 1808.** Sec. Czerepanov (1995)

≡ *Tunicarigida* (M.Bieb.) Raf.﻿, Fl. Tellur. 2: 54. 1837 syn. sec. POWO. Plants of the World Online. Facilitated by the Royal Botanic Gardens, Kew.

***Dianthusrobustus* Boiss. & Kotschy ex Boiss.﻿, Fl. Orient. 1: 492. 1867.** Sec. Marhold (2011)

= *Dianthussuperbiens* Kotschy ex Boiss.﻿, Fl. Orient. 1: 492. 1867 syn. sec. WFO 2018

***Dianthusrogowiczii* Kleopow﻿ in Izv. Kievsk. Bot. Sada 12–13: 160. 1931.** Sec. Czerepanov (1995)

***Dianthusroseoluteus* Velen. in Oesterr. Bot. Z. 36: 226. 1886.** Sec. Marhold (2011)

≡ Dianthuscampestrissubsp.roseoluteus (Velen.) Stoj. & Acht.﻿, Sborn. Blghar. Akad. Nauk 29(2): 16. 1935 syn. sec. Marhold (2011)

= *Dianthuspurpureoluteus* Velen.﻿, Fl. Bulg.: 73. 1891 syn. sec. Marhold (2011)

***Dianthusrudbaricus* Assadi﻿ in Iranian J. Bot. 3: 38. 1985.** Sec. Rechinger (1988)

***Dianthusrupicola* Biv.﻿, Sicul. Pl. 1: 31. 1806.** Sec. Bernal et al. (1990)

= *Dianthusbisignanii* Ten.﻿, Cat. Piante Barra: 13. 1805 syn. sec. Bernal et al. (1990)

= *Dianthussuffruticosus* Willd.﻿, Enum. Pl.: 466. 1809 syn. sec. WFO 2018

= *Dianthusinvolucratus* Poir.﻿, Encycl. Suppl. 4: 132. 1816 syn. sec. WFO 2018

= *Dianthusarborescens* Hoffmanns.﻿, Verz. Pfl.-Kult.: 56. 1824 syn. sec. WFO 2018

= *Dianthusbertolonii* J.Woods, Tourists Fl.: 45. 1850 syn. sec. Kew WCVP (2019) ≡ Dianthusrupicolavar.bertolonii (J.Woods) Arcang.﻿, Comp. Fl. Ital.: 85. 1882 syn. sec. Kew WCVP (2019)

= *Dianthushermaeensis* Coss.﻿, Ill. Fl. Atlant. 1: 129. 1890 syn. sec. Bernal et al. (1990) ≡ Dianthusrupicolavar.hermaeensis (Coss.) F.N.Williams﻿ in J. Linn. Soc., Bot. 29: 363. 1893 syn. sec. Kew WCVP (2019) ≡ Dianthusrupicolasubsp.hermaeensis (Coss.) O.Bolòs & Vigo﻿ in Butl. Inst. Catalana Hist. Nat., Secc. Bot. 38(1): 187. 1974 syn. sec. Bernal et al. (1990)

**Dianthusrupicolasubsp.aeolicus (Lojac.) Brullo & Miniss.﻿ in Inform. Bot. Ital. 33: 539. 2001 [“2002”].** Sec. Iamonico (2013)

≡ *Dianthusaeolicus* Lojac.﻿, Fl. Sicul. 1(1): 163. 1888 syn. sec. Iamonico (2013)

**Dianthusrupicolasubsp.bocchoriana L.Llorens & Gradaille﻿ in Candollea 46: 389. 1991.** Sec. New taxa described to the Flora Iberica region after publication of the respective volume. Published at http://www.floraiberica.es/eng/miscelania/nuevos_taxones.php

**Dianthusrupicolasubsp.lopadusanus Brullo & Miniss.﻿ in Inform. Bot. Ital. 33: 541. 2002 [“2001”].** Sec. Iamonico (2013)

**Dianthusrupicolasubsp.rupicola**﻿

***Dianthusruprechtii* Schischk. ex Grossh.﻿, Fl. Kavkaza 2: 432. 1930.** Sec. Kuzmina & Nersesyan (2012)

= Dianthuscarthusianorumvar.caucasicus Rupr.﻿, Fl. Caucasi: 174. 1869 syn. sec. Kuzmina & Nersesyan (2012)

***Dianthussachalinensis* Barkalov & Prob.﻿, Fl. Ross. Dalnego Vostoka: 444. 2006.** Sec. Barkalov & Probatova (2006)

***Dianthussaetabensis* Rouy﻿ in Bull. Soc. Bot. France 29: 44. 1882.** Sec. Mateo Sanz & Crespo (2008)

**Dianthussaetabensissubsp.contestanus (M.B.Crespo & Mateo) M.B.Crespo & Mateo﻿ in Flora Montiber. 40: 65. 2008.** Sec. Mateo Sanz & Crespo (2008)

≡ Dianthushispanicussubsp.contestanus M.B.Crespo & Mateo﻿ in Flora Montiber. 20: 8. 2002 syn. sec. Mateo Sanz & Crespo (2008)

**Dianthussaetabensissubsp.saetabensis**﻿

***Dianthussahandicus* Assadi﻿ in Iranian J. Bot. 3: 45. 1985.** Sec. Rechinger (1988)

***Dianthussajanensis* (Baikov) Czepinoga﻿, Konspekt Fl. Irkutsk. Obl.: 116. 2008.** Sec. Chepinoga et al. (2008)

≡ Dianthussuperbussubsp.sajanensis Baikov﻿ in Bot. Zhurn. (Moscow & Leningrad) 77(9): 80. 1992 syn. sec. Chepinoga et al. (2008)

***Dianthussancarii* Hamzaoğlu & Koç﻿ in Biol. Diversity Conservation 11(1): 31. 2018.** Sec. Hamzaoğlu & Koç (2018)

***Dianthussardous* Bacch., Brullo, Casti & Giusso﻿ in Feddes Repert. 116: 271. 2005.** Sec. Bacchetta et al. (2010)

**Dianthus×saxatilis F.W.Schmidt﻿ in Neuere Abh. Königl. Böhm. Ges. Wiss. 1: 28. 1790.** Sec. WFO 2018

***Dianthusscardicus* Wettst.﻿ in Biblioth. Bot. 26: 31. 1891.** Sec. Marhold (2011)

= Dianthusscardicusvar.incisus Micevski﻿ in Prilozi Oddel. Biol. Med. Nauki Makedonska Akad. Nauk. Umet. 8: 44. 1987 [“1990”] syn. sec. WFO 2018

= Dianthusnitidussubsp.lakusicii Wraber﻿ in Biol. Vestn. 36: 97. 1988 syn. sec. WFO 2018

***Dianthusschemachensis* Schischk.﻿ in Trudy Geobot. Obsl. Pastb. S.S.R. Azerbaidzhn, Ser. A., Zimn. Pabstb. 7: 90. 1931.** Sec. Kuzmina & Nersesyan (2012)

***Dianthusseguieri* Vill.﻿, Hist. Pl. Dauphiné 1: 330. 1786.** Sec. Bernal et al. (1990)

≡ *Sileneseguieri* (Vill.) E.H.L.Krause﻿, Deutschl. Fl. Abbild., ed. 2, 5: 109. 1901 syn. sec. Kew WCVP (2019)

= *Dianthusasper* Willd.﻿, Enum. Pl.: 466. 1809 syn. sec. WFO 2018 ≡ Dianthusseguierivar.asper (Willd.) W.D.J.Koch﻿, Syn. Fl. Germ. Helv. 1: 96. 1835 syn. sec. Kew WCVP (2019)

= Dianthusaspervar.angustifolius Ser.﻿, Prodr. 1: 357. 1824 syn. sec. Kew WCVP (2019)

= Dianthusseguierif.longibracteatus Regel﻿, Index Seminum (St. Petersburg, Petropolitanus) 1863: 34. 1863 syn. sec. Kew WCVP (2019)

= Dianthusseguierivar.viscidus Regel﻿, Index Seminum (St. Petersburg, Petropolitanus) 1863: 40. 1863 syn. sec. Kew WCVP (2019)

= *Cylichnanthusciliatus* Dulac﻿, Fl. Hautes-Pyrénées: 261. 1867 syn. sec. POWO. Plants of the World Online. Facilitated by the Royal Botanic Gardens, Kew.

= Dianthussylvaticusvar.pseudocollinus P.Fourn.﻿, Quatre Fl. France: 330. 1936 syn. sec. Kew WCVP (2019) ≡ Dianthusseguierisubsp.pseudocollinus (P.Fourn.) Jauzein﻿, Biocosme Mésogéen 27: 114. 2010 syn. sec. Kew WCVP (2019)

= Dianthusseguierivar.subaggregatus Albov﻿ syn. sec. IPNI ≡ *Dianthussubaggregatus* (Albov) Schischk. ex Kem.-Nath.﻿ in Vеstn. Tiflissk. Bot. Sada, n.s., 5: 12. 1931 syn. sec. POWO. Plants of the World Online. Facilitated by the Royal Botanic Gardens, Kew.

**Dianthusseguierisubsp.glaber Čelak.﻿, Prodr. F. Böhmen 3: 507. 1875.** Sec. Marhold (2011)

**Dianthusseguierisubsp.requienii (Godr.) M.Bernal, Laínz & Muñoz Garm.﻿ in Anales Jard. Bot. Madrid 44: 569. 1987.** Sec. Bernal et al. (1990)

≡ *Dianthusrequienii* Godr.﻿, Fl. France 1: 234. 1847 syn. sec. Bernal et al. (1990) ≡ Dianthusfurcatussubsp.requienii (Godr.) Kerguélen﻿ in Lejeunia 120: 80. 1987 syn. sec. Bernal et al. (1990)

= *Cylichnanthusunibiflorus* Dulac﻿, Fl. Hautes-Pyrénées: 261. 1867 syn. sec. POWO. Plants of the World Online. Facilitated by the Royal Botanic Gardens, Kew.

= *Dianthusarragonensis* Timb.-Lagr. ex Nyman﻿, Consp. Fl. Eur. 1: 105. 1878 syn. sec. WFO 2018

= *Dianthusgerundensis* Sennen & Pau﻿ in Bol. Soc. Aragonesa Ci. Nat. 4: 309. 1905 syn. sec. WFO 2018 ≡ Dianthusseguierivar.gerundensis (Sennen & Pau) O.Bolòs & Vigo﻿ in Butl. Inst. Catalana Hist. Nat., Secc. Bot. 38(1): 88. 1974 syn. sec. WFO 2018

= *Dianthusgautieri* Sennen﻿ in Bull. Acad. Int. Geogr. Bot. 21: 107. 1911 syn. sec. Bernal et al. (1990) ≡ Dianthusseguierisubsp.gautieri (Sennen) Tutin﻿ in Feddes Repert. Spec. Nov. Regni Veg. 68: 189. 1963 syn. sec. Bernal et al. (1990) ≡ Dianthusseguierivar.gautieri (Sennen) O.Bolòs & Vigo﻿ in Butl. Inst. Catalana Hist. Nat., Secc. Bot. 38: 88. 1974 syn. sec. this publication

= *Dianthuscadevallii* Sennen & Pau﻿ in Bull. Acad. Int. Geogr. Bot. 24: 237. 1914 syn. sec. Bernal et al. (1990) ≡ Dianthusseguierisubsp.cadevallii (Sennen & Pau) O.Bolòs & Vigo﻿ in Butl. Inst. Catalana Hist. Nat., Secc. Bot. 38(1): 88. 1974 syn. sec. Bernal et al. (1990)

= Dianthus×paui Sennen﻿ in Bull. Acad. Int. Geogr. Bot. 24: 236. 1914 syn. sec. Bernal et al. (1990)

= *Dianthusqueraltii* Sennen﻿ in Bol. Soc. Ibér. Ci. Nat. 25: 210. 1926 [“1927”] syn. sec. WFO 2018 ≡ Dianthusseguierivar.queraltii (Sennen) O.Bolòs & Vigo﻿ in Butl. Inst. Catalana Hist. Nat., Secc. Bot. 38(1): 88. 1974 syn. sec. WFO 2018

**Dianthusseguierisubsp.seguieri**﻿

***Dianthusseidlitzii* Boiss.﻿, Fl. Orient. 1: 506. 1867.** Sec. Rechinger (1988)

= *Dianthuspusillus* Freyn & Sint.﻿ in Bull. Herb. Boiss. 3: 75. 1895 syn. sec. Rechinger (1988)

***Dianthussemenovii* (Regel & Herder) Vierh.﻿ in Sitzungsber. Kaiserl. Akad. Wiss., Math.-Naturwiss. Cl., Abt. 1, 107: 1147. 1898.** Sec. Dequan & Turland (2001)

≡ Dianthusalpinusvar.semenovii Regel & Herder﻿ in Bull. Soc. Imp. Naturalistes Moscou 39(1): 531. 1866 syn. sec. Dequan & Turland (2001)

***Dianthusseravschanicus* Schischk.﻿, Fl. URSS 6: 898. 1936.** Sec. Czerepanov (1995)

***Dianthusserotinus* Waldst. & Kit.﻿ in Descr. Icon. Pl. Hung. 2: 188. 1804.** Sec. Czerepanov (1995)

***Dianthusserpentinus* Hamzaoğlu﻿ in Nordic J. Bot. 33: 59. 2014.** Sec. Hamzaoğlu et al. (2015)

***Dianthusserratifolius* Sm.﻿, Fl. Graec. Prodr. 1(2): 287. 1809.** Sec. Dimopoulos et al. (2013)

= *Dianthusnazaraeus* E.D.Clarke﻿, Trav. Var. Eur. 2: 420. 1812 syn. sec. WFO 2018

**Dianthusserratifoliussubsp.abbreviatus (Heldr. ex Halácsy) Strid﻿, Mount. Fl. Greece 1: 179. 1986.** Sec. Dimopoulos et al. (2013)

≡ Dianthusserratifoliusvar.abbreviatus Heldr. ex Halácsy﻿ syn. sec. Dimopoulos et al. (2013)

**Dianthusserratifoliussubsp.serratifolius**﻿

***Dianthusserrulatus* Desf.﻿, Fl. Atlant. 1: 346. 1798.** Sec. Marhold (2011)

= Dianthusserrulatusvar.strictus Maire﻿ in Bull. Soc. His. Nat. Afrique N. 23: 169. 1932 syn. sec. Kew WCVP (2019)

= Dianthusserrulatusvar.subsimplex F.N.Williams ex Maire﻿ in Bull. Soc. His. Nat. Afrique N. 23: 169. 1932 syn. sec. Kew WCVP (2019)

= *Dianthustaygeteus* Quézel & Contandr.﻿ in Taxon 16: 239. 1967 syn. sec. Marhold (2011)

**Dianthusserrulatussubsp.cyrenaicus (Pamp.) Maire﻿, Fl. Afr. Nord 10: 303. 1963.** Sec. Marhold (2011)

≡ Dianthusserrulatusvar.cyrenaicus Pamp.﻿ in Arch. Bot. (Forlì) 12: 24. 1936 syn. sec. Marhold (2011)

**Dianthusserrulatussubsp.macranthus Maire﻿.** Sec. Marhold (2011)

= Dianthusserrulatusvar.broteri Batt.﻿, Fl. Algérie Tunisie: 61. 1905 syn. sec. Kew WCVP (2019)

= *Dianthusmesanidum* Litard. & Maire﻿, Mém. Soc. Sci. Nat. Maroc 6: 7. 1924 syn. sec. Marhold (2011) ≡ Dianthusserrulatusvar.mesanidum (Litard. & Maire) Maire﻿, Fl. Afrique N. 10: 303. 1963 syn. sec. Kew WCVP (2019)

**Dianthusserrulatussubsp.serrulatus**﻿

***Dianthussessiliflorus* Boiss.﻿, Fl. Orient. Suppl.: 78. 1888.** Sec. Marhold (2011)

***Dianthussetisquameus* Hausskn. & Bornm.﻿ in Mitt. Geogr. Ges. (Thüringen) Jena 9: 15. 1891.** Sec. Marhold (2011)

***Dianthusshinanensis* (Yatabe) Makino﻿ in Bot. Mag. (Tokyo) 17: 58. 1903.** Sec. Zoku (1965)

≡ Dianthusbarbatusvar.shinanensis Yatabe﻿ in Bot. Mag. (Tokyo) 6: 132. 1892 syn. sec. Zoku (1965)

= *Dianthustakenakae* Honda﻿ in Bot. Mag. (Tokyo) 44: 670. 1930 syn. sec. Zoku (1965)

= Dianthusshinanensisf.alpinus Hid.Takah. ex T.Shimizu﻿, Fl. Nagano Pref.: 1505. 1997 syn. sec. WFO 2018

***Dianthussiculus* C.Presl﻿, Delic. Prag.: 59. 1822.** Sec. Bacchetta et al. (2010)

≡ Dianthuscaryophyllussubsp.siculus (C.Presl) Arcang.﻿, Comp. Fl. Ital. ed. 2: 306. 1894 syn. sec. Bacchetta et al. (2010) ≡ Dianthuscaryophyllusvar.siculus (C.Presl) Fiori﻿, Fl. Italia 1: 379. 1898 syn. sec. Bacchetta et al. (2010) ≡ Dianthussylvestrissubsp.siculus (C.Presl) Tutin﻿ in Feddes Repert. Spec. Nov. Regni Veg. 68: 190. 1963 syn. sec. Bacchetta et al. (2010)

= *Dianthuskremeri* Boiss. & Reut.﻿, Pugill. Pl. Afr. Bor. Hispan.: 21. 1852 syn. sec. African Plant Database (version 3.4.0)

= Dianthussiculusvar.lanceolatus Pau﻿ in Trab. Mus. Ci. Nat., Ser. Bot., 11: 22. 1917 syn. sec. African Plant Database (version 3.4.0) ≡ Dianthuscaryophyllusvar.lanceolatus (Pau) Maire﻿, Cat. Pl. Maroc 2: 238. 1932 syn. sec. African Plant Database (version 3.4.0)

= Dianthuscaryophyllusvar.transiens Maire﻿ in Bull. Soc. His. Nat. Afrique N. 20: 16. 1929 syn. sec. African Plant Database (version 3.4.0) ≡ Dianthuscaryophyllusf.transiens (Maire) Maire﻿, Fl. Afrique N. 10: 319. 1963 syn. sec. this publication

= Dianthuscaryophyllusvar.puberulus Faure & Maire﻿ in Bull. Soc. His. Nat. Afrique N. 22: 280. 1931 syn. sec. African Plant Database (version 3.4.0)

= *Dianthusmauritii* Sennen﻿, Diagn. Nouv.: 295. 1936 syn. sec. African Plant Database (version 3.4.0)

= Dianthuskremerivar.trichodontus Faure & Maire﻿ in Bull. Soc. His. Nat. Afrique N. 30: 333. 1939 syn. sec. African Plant Database (version 3.4.0)

***Dianthussimulans* Stoj. & Stef. ex Stef. & Jordanov﻿ in Magyar Bot. Lapok 32: 1. 1933.** Sec. Dimopoulos et al. (2013)

≡ Dianthusgracilissubsp.simulans (Stoj. & Stef.) Stoj. & Acht.﻿ in Sborn. Bălg. Akad. Nauk. 29(2): 71. 1935 syn. sec. Dimopoulos et al. (2013)

***Dianthussinaicus* Boiss.﻿, Diagn. Pl. Orient. ser. 1 1: 23. 1843.** Sec. Marhold (2011)

= *Dianthusmultisquamatus* Hochst. ex Boiss.﻿, Fl. Orient. 1: 497. 1867 syn. sec. WFO 2018

***Dianthussiphonocalyx* Blakelock﻿ in Kew Bull. 3: 397. 1948 [“1949”].** Sec. Rechinger (1988)

***Dianthussomanus* Oskay﻿ in Phytotaxa 347: 264. 2018.** Sec. Oskay (2018)

***Dianthussphacioticus* Boiss. & Heldr.﻿ in Boissier, Diagn. Pl. Orient., ser. 1, 8: 70. 1849.** Sec. Dimopoulos et al. (2013)

= *Dianthusleucophaeus* Sieber﻿, Reise Kreta 2: 320. 1823, nom. illeg. syn. sec. WFO 2018 [non *Dianthusleucophaeus* Sm.﻿]

***Dianthusspiculifolius* Schur﻿, Enum. Pl. Transsilv.: 98. 1866.** Sec. Czerepanov (1995)

***Dianthussquarrosus* M.Bieb.﻿, Fl. Taur.-Caucas. 1: 331. 1808.** Sec. Czerepanov (1995)

= *Dianthusarenarius* Pall.﻿, Reise Russ. Reich. 3: 600. 1776 syn. sec. Czerepanov (1995) [non *Dianthusarenarius* L.﻿]

= *Dianthusmussini* Hornem.﻿ in Hort. Bot. Hafn. 1: 408. 1813 syn. sec. Czerepanov (1995)

= *Dianthusrecurvus* Fisch. ex Ledeb.﻿, Fl. Ross. 1: 284. 1842 syn. sec. Czerepanov (1995)

= *Dianthussabulosus* Willd. ex Ledeb.﻿, Fl. Ross. 1: 284. 1842 syn. sec. Czerepanov (1995)

***Dianthusstamatiadae* Rech.f.﻿ in Bot. Not. 124: 77. 1971.** Sec. Dimopoulos et al. (2013)

***Dianthusstapfii* Lemperg﻿ in Repert. Spec. Nov. Regni Veg. 50: 261. 1941.** Sec. Rechinger (1988)

***Dianthusstellaris* Camarda﻿ in Parlatorea 6: 87. 2003.** Sec. Camarda (2003)

≡ Dianthussiculussubsp.stellaris (Camarda) Arrigoni﻿ in Parlatorea 7: 20. 2005 syn. sec. WFO 2018

***Dianthusstenocephalus* Boiss.﻿, Diagn. Pl. Orient. ser. 1 1: 19. 1843.** Sec. Rechinger (1988)

= *Dianthusmacrolepis* Fenzl ex Boiss.﻿, Diagn. Pl. Orient., ser. 1, 8: 64. 1849 syn. sec. WFO 2018

***Dianthusstenopetalus* Griseb.﻿, Spic. Fl. Rumel. 1: 187. 1843.** Sec. Dimopoulos et al. (2013)

= *Dianthusgeticus* Kulcz.﻿ in Rozpr. Wydz. Mat.-Przyr Polsk Akad. Umiejetn., Dzial A/B, Nauki Mat-Fiz. Biol. 59: 37. 1923 syn. sec. WFO 2018

***Dianthusstepanovae* Barkalov & Prob.﻿, Fl. Ross. Dalnego Vostoka: 444. 2006.** Sec. Barkalov & Probatova (2006)

***Dianthussternbergii* Sieber ex Capelli﻿, Cat. Stirp.: 24. 1821.** Sec. Marhold (2011)

≡ Dianthusmonspessulanusvar.sternbergii (Sieber ex Capelli) Tanfani﻿, Fl. Ital. 9: 276. 1892 syn. sec. Kew WCVP (2019) ≡ Dianthushyssopifoliussubsp.sternbergii (Sieber ex Capelli) Graebn. & P.Graebn.﻿ in Ascherson & Graebn., Syn. Mitteleur. Fl. 5(2): 436. 1922 syn. sec. Marhold (2011)

= *Dianthuswaldsteinii* Sternb.﻿ in Flora 9(1 Beibl.): 73. 1826 syn. sec. Marhold (2011)

= Dianthusmonspessulanussubsp.sternbergii Hegi﻿ syn. sec. Marhold (2011)

***Dianthusstramineus* Boiss. & Heldr.﻿ in Boissier, Diagn. Pl. Orient., ser. 1, 8: 70. 1849.** Sec. Marhold (2011)

***Dianthusstribrnyi* Velen.﻿ in Sitzungsber. Königl. Böhm. Ges. Wiss., Math.-Naturwiss. Cl. 1892: 15. 1893.** Sec. Marhold (2011)

≡ Dianthusmoesiacussubsp.stribrnyi (Velen.) Stoj. & Acht.﻿ in Sborn. Bălg. Akad. Nauk. 29(2): 53. 1935 syn. sec. Marhold (2011)

***Dianthusstrictus* Banks & Sol.﻿, Nat. Hist. Aleppo ed. 2, 2: 252. 1794.** Sec. Rechinger (1988)

= *Dianthuspolycladus* Boiss.﻿, Diagn. Pl. Orient., ser. 1, 8: 65. 1849 syn. sec. Rechinger (1988)

= *Dianthusquadrilobus* Boiss.﻿, Asie Min., Bot. 1: 222. 1860 syn. sec. Rechinger (1988)

= *Dianthussulcatus* Boiss.﻿, Fl. Orient. 1: 483. 1867 syn. sec. Rechinger (1988)

= *Dianthushalepensis* Bornm.﻿ in Repert. Spec. Nov. Regni Veg. Beih. 89: 91. 1936 syn. sec. Rechinger (1988)

**Dianthusstrictussubsp.multipunctatus (Ser.) Mouterde ex Greuter & Burdet﻿ in Willdenowia 12: 187. 1982.** Sec. Rechinger (1988)

≡ *Dianthusmultipunctatus* Ser.﻿, Prodr. 1: 362. 1824 syn. sec. Rechinger (1988)

= *Dianthuslineolatus* Bové ex Delile﻿ in Ann. Sci. Nat., Bot. II, 7: 286. 1837 syn. sec. Rechinger (1988)

**Dianthusstrictussubsp.strictus**﻿

**Dianthusstrictussubsp.sublaevisD.F.Chamb.﻿ in Edinburgh J. Bot. 51: 56. 1994.** Sec. Chamberlain et al. (1994)

**Dianthusstrictussubsp.troodi (Post) B.F.Osoriol & Seraphim ex Greuter & Burdet﻿ in Willdenowia 12: 187. 1982.** Sec. Rechinger (1988)

≡ Dianthusmultipunctatusvar.troodi Post﻿ in Mém. Herb. Boissier 1(18): 91. 1900 syn. sec. Rechinger (1988) ≡ Dianthusstrictusvar.troodi (Post) S.S.Hooper﻿, Fl. Cyprus 1: 806. 1977 syn. sec. Rechinger (1988)

**Dianthusstrictussubsp.velutinus (Boiss.) Mouterde ex Greuter & Burdet﻿ in Willdenowia 12: 187. 1982.** Sec. Rechinger (1988)

≡ Dianthusmultipunctatusvar.velutinus Boiss.﻿, Diagn. Pl. Orient., ser. 1, 8: 65. 1849 syn. sec. Rechinger (1988)

**Dianthusstrictusvar.axilliflorus (Fenzl) Reeve﻿ in Notes Roy. Bot. Gard. Edinburgh 28: 19. 1967.** Sec. Rechinger (1988)

≡ *Dianthusaxilliflorus* Fenzl﻿, Pug. Pl. Nov. Syr.: 10. 1842 syn. sec. Rechinger (1988) ≡ Dianthusmultipunctatusvar.axilliflorus (Fenzl) Boiss.﻿, Fl. Orient. 1: 483. 1867 syn. sec. Rechinger (1988)

= *Dianthusbitlisianus* Kotschy ex Boiss.﻿, Fl. Orient. 1: 483. 1867 syn. sec. Rechinger (1988)

**Dianthusstrictusvar.gracilior (Boiss.) Reeve﻿ in Notes Roy. Bot. Gard. Edinburgh 28: 19. 1967.** Sec. Rechinger (1988)

≡ Dianthusmultipunctatusvar.gracilior Boiss.﻿, Fl. Orient. 1: 483. 1867 syn. sec. Rechinger (1988)

= *Dianthusstriatellus* Fenzl﻿, Pug. Pl. Nov. Syr.: 10. 1842 syn. sec. Rechinger (1988)

= *Dianthuspaniculatus* Pau﻿ in Trab. Mus. Ci. Nat., Ser. Bot., 14: 9. 1918 syn. sec. Rechinger (1988)

**Dianthusstrictusvar.subenervis (Boiss.) Reeve﻿ in Notes Roy. Bot. Gard. Edinburgh 28: 19. 1967.** Sec. Rechinger (1988)

≡ Dianthusmultipunctatusvar.subenervis Boiss.﻿, Fl. Orient. 1: 483. 1867 syn. sec. Rechinger (1988)

***Dianthusstrymonis* Rech.f.﻿ in Bot. Jahrb. Syst. 69: 450. 1939.** Sec. Dimopoulos et al. (2013)

***Dianthussubacaulis* Vill.﻿, Hist. Pl. Dauphiné 3: 597. 1789.** Sec. Marhold (2011)

≡ Dianthusvirgineusvar.subacaulis (Vill.) Ser.﻿, Prodr. 1: 361. 1824 syn. sec. this publication

***Dianthussubaphyllus* (Lemperg) Rech.f.﻿, Fl. Iran. 163: 170. 1988.** Sec. Rechinger (1988)

≡ Dianthustabrisianusvar.subaphyllus Lemperg﻿ in Repert. Spec. Nov. Regni Veg. 50: 259. 1941 syn. sec. Rechinger (1988)

**Dianthus×subfissus Rouy & Foucaud﻿, Fl. France 3: 184. 1896.** Sec. Bernal et al. (1990)

***Dianthussubscabridus* Lincz.﻿, Fl. Uzbekist. 2: 525. 1953.** Sec. Czerepanov (1995)

***Dianthussubulosus* Conrath & Freyn in Bull. Herb. Boiss. 3: 76. 1895.** Sec. Nersesyan (2011)

***Dianthussuperbus* L.﻿, Amoen. Acad. 4: 272. 1759.** Sec. Dimopoulos et al. (2013)

≡ *Caryophyllussuperbus* (L.) Moench﻿, Methodus: 59. 1794 syn. sec. Kew WCVP (2019); ≡ *Silenesuperba* (L.) E.H.L.Krause﻿, Deutschl. Fl. Abbild., ed. 2, 5: 116. 1901 syn. sec. POWO. Plants of the World Online. Facilitated by the Royal Botanic Gardens, Kew.; ≡ Dianthussuperbusvar.superbus﻿ syn. sec. WFO 2018 – *Plumariasuperba* (L.) Opiz﻿, Seznam: 75. 1852, nom. inval. syn. sec. Kew WCVP (2019);

= *Dianthusfimbriatus* Lam.﻿, Fl. Franç. 2: 538. 1779 syn. sec. WFO 2018 ≡ *Cylichnanthusfimbriatus* (Lam.) Dulac﻿, Fl. Hautes-Pyrénées: 262. 1867 syn. sec. POWO. Plants of the World Online. Facilitated by the Royal Botanic Gardens, Kew.

= *Dianthusmultifidus* Gilib.﻿, Fl. Lit. Inch. 2: 162. 1782 syn. sec. WFO 2018

= *Dianthuscontortus* Sm.﻿, Cycl.: 11. 1808 syn. sec. WFO 2018

= Dianthussuperbusvar.rubicundus Ser.﻿, Prodr. 1: 365. 1824 syn. sec. Kew WCVP (2019)

= *Dianthusplumarius* Gunnerus ex Spreng.﻿, Syst. Veg., ed. 16, 2: 379. 1825 syn. sec. WFO 2018

= *Dianthusschizopetalus* Wallr.﻿ in Linnaea 14: 570. 1840 syn. sec. WFO 2018

= *Dianthuswimmeri* Wich.﻿ in Jahresber. Schles. Ges. Vaterl. Cult. 1854: 75. 1854 syn. sec. WFO 2018

= Dianthussuperbusvar.subobtusus Regel & Herder﻿ in Bull. Soc. Imp. Naturalistes Moscou 39(1): 532. 1866 syn. sec. Kew WCVP (2019)

= Dianthussuperbussubsp.silvestris Čelak.﻿, Prodr. Fl. Böhmen 3: 508. 1875 syn. sec. Kew WCVP (2019)

= *Dianthusszechuensis* F.N.Williams﻿ in J. Linn. Soc., Bot. 34: 428. 1899 syn. sec. WFO 2018

= Dianthussuperbusf.albiflorus Iljinski﻿ in Trudy Bot. Muz. Imp. Akad. Nauk 14: 25. 1915 syn. sec. WFO 2018

= Dianthussuperbusf.albiflorus Honda﻿ in Bot. Mag. (Tokyo) 52: 140. 1938, nom. illeg. syn. sec. Dimopoulos et al. (2013)

= Dianthussuperbusf.albiflorus Tatew.﻿, Veg. Shikotan Is.: 32. 1940, nom. illeg. syn. sec. Dimopoulos et al. (2013)

= Dianthussuperbusf.albus Popov﻿ in Konspekt Fl. Pober. Baikal: 213. 1966 syn. sec. Kew WCVP (2019)

= Dianthussuperbusf.leucanthus T.Shimizu﻿ in J. Phytogeogr. Taxon. 37: 120. 1989 syn. sec. WFO 2018

= Dianthussuperbussubsp.norvegicus M.Kuzmina, Fl. Vostoch. Evropy 11: 294. 2004 syn. sec. WFO 2018

= *Dianthusrevolutus* Tausch﻿ in Flora 13: 245. 1830 syn. sec. WFO 2018 – *Plumariarevoluta* (Tausch) Opiz﻿, Seznam: 75. 1852, nom. inval. syn. sec. Kew WCVP (2019)

**Dianthussuperbussubsp.alpestris Kablík. ex Čelak.﻿, Prodr. Fl. Böhmen: 508. 1875.** Sec. Marhold (2011)

= Dianthussuperbusvar.speciosus Rchb.﻿, Fl. Germ. Excurs.: 808. 1832 syn. sec. WFO 2018 ≡ *Dianthusspeciosus* (Rchb.) Rchb.﻿ in Icon. Fl. Germ. Helv. 6: 46. 1844 syn. sec. WFO 2018 ≡ Dianthussuperbussubsp.speciosus (Rchb.) Hayek﻿, Sched. Fl. Stiriac. 11–12: 9. 1907 syn. sec. Marhold (2011) ≡ Dianthussuperbusf.speciosus (Rchb.) Bolzon﻿ in Nuovo Giorn. Bot. Ital., n.s., 21: 180. 1914 syn. sec. Kew WCVP (2019)

= Dianthussuperbusvar.monticola Makino﻿ in Bot. Mag. (Tokyo) 17: 59. 1903 syn. sec. WFO 2018

= Dianthussuperbusvar.bibracteolata Koidz.﻿ in Icon. Pl. Koisikav. 3(4): t. 183. 1916 syn. sec. WFO 2018 ≡ Dianthussuperbusf.bibracteolata (Koidz.) Tatew.﻿ in J. Sapporo Soc. Agric. 121: 256. 1934 syn. sec. WFO 2018

= Dianthussuperbusf.chionanthus Okuyama﻿ in J. Jap. Bot. 30: 42. 1955 syn. sec. WFO 2018

**Dianthussuperbussubsp.autumnalis Oberd.﻿, Pfl. Exkurs. Fl., ed. 4: 359. 1979.** Sec. Marhold (2011)

**Dianthussuperbussubsp.stenocalyx (Trautv. ex Juz.) Kleopow﻿ in Izv. Kievsk. Bot. Sada 14: 137. 1932.** Sec. Marhold (2011)

≡ *Dianthusstenocalyx* Trautv. ex Juz.﻿ in Mem. Inst. Agron. For. Belarus 4: 212. 1925 syn. sec. Marhold (2011)

**Dianthussuperbussubsp.superbus**﻿

**Dianthussuperbusvar.amoena Nakai﻿ in Bot. Mag. (Tokyo) 44: 520. 1930.** Sec. Kew WCVP (2019)

≡ Dianthussuperbusvar.alpestris Nakai﻿ in Bot. Mag. (Tokyo) 36: 63. 1922, nom. illeg. syn. sec. Kew WCVP (2019)

***Dianthussylvaticus* Hoppe ex Willd.﻿, Enum. Pl.: 467. 1809.** Sec. Danihelka et al. (2012)

≡ Dianthusseguierivar.sylvaticus (Hoppe ex Willd.) W.D.J.Koch﻿, Syn. Fl. Germ. Helv. 1: 96. 1835 syn. sec. this publication ≡ Dianthusseguierisubsp.sylvaticus (Hoppe ex Willd.) Arcang.﻿, Comp. Fl. Ital.: 84. 1882 syn. sec. this publication

***Dianthussylvestris* Wulfen﻿ in Collectanea 1: 237. 1786.** Sec. Dimopoulos et al. (2013)

≡ Dianthuscaryophyllussubsp.sylvestris (Wulfen) Rouy & Foucaud﻿, Fl. France 3: 193. 1896 syn. sec. Kew WCVP (2019) ≡ *Silenesylvestris* (Wulfen) E.H.L.Krause﻿, Deutschl. Fl. Abbild., ed. 2, 5: 112. 1901, nom. illeg. syn. sec. Kew WCVP (2019);

= ?Dianthuscaryophyllusvar.inodorus L.﻿, Sp. Pl. 1: 410. 1753 syn. sec. Domina et al. (2021) ≡ ?*Dianthusinodorus* (L.) Gaertn.﻿, Fruct. Sem. Pl. 2: 227. 1791 syn. sec. Domina et al. (2021)

= *Dianthusrupestris* L.f.﻿, Suppl. Pl.: 240. 1782 syn. sec. WFO 2018

= *Dianthuscaryophylloides* Schult.﻿, Observ. Bot.: 78. 1809 syn. sec. WFO 2018

= *Dianthuswulfenii* F.Dietr.﻿, Nachtr. Vollst. Lex. Gärtn. 2: 669. 1816 syn. sec. WFO 2018

= *Dianthusfrigidus* Zucc.﻿ in Flora 7: 283. 1824 syn. sec. WFO 2018

= Dianthussylvestrisvar.imbricatus Gaudin﻿, Fl. Helv. 3: 152. 1828 syn. sec. Kew WCVP (2019)

= Dianthussylvestrisvar.uniflorus Gaudin﻿, Fl. Helv. 3: 152. 1828 syn. sec. Kew WCVP (2019)

= Dianthussylvestrisvar.humilior W.D.J.Koch﻿, Syn. Fl. Germ. Helv. 1: 97. 1835 syn. sec. Kew WCVP (2019)

= Dianthussylvestrisvar.subacaulis W.D.J.Koch﻿, Syn. Fl. Germ. Helv. 1: 97. 1835 syn. sec. Kew WCVP (2019)

= *Dianthussaxicola* Jord.﻿, Mém. Acad. Sci. Lyon, Sect. Sci. 1: 241. 1851 syn. sec. WFO 2018

= *Dianthusaggericola* Jord.﻿, Annot. Fl. France Allemagne: 48. 1855 syn. sec. Kew WCVP (2019)

= *Dianthusaggericolus* Jord.﻿, Annot. Fl. France Allemange: 48. 1855 syn. sec. WFO 2018

= *Dianthusconsimilis* Jord.﻿, Annot. Fl. France Allemange: 47. 1855 syn. sec. WFO 2018

= *Dianthusguyetanii* Jord.﻿, Annot. Fl. France Allemange: 46. 1855 syn. sec. WFO 2018

= *Dianthusorophilus* Jord.﻿, Annot. Fl. France Allemange: 43. 1855 syn. sec. WFO 2018

= *Dianthusreuteri* Jord.﻿, Annot. Fl. France Allemange: 49. 1855 syn. sec. WFO 2018

= *Dianthuspapillosus* Vis. & Pančić﻿ in Mem. Reale Ist. Veneto Sci. 10: 434. 1861 syn. sec. Marhold (2011) ≡ Dianthussylvestrisf.papillosus (Vis. & Pančić) Beck﻿ in Glasn. Zemaljsk. Muz. Bosni Hercegovini 21: 175. 1909 syn. sec. this publication

= *Dianthusbrevicalyx* Beck﻿ in Ann. K. K. Naturhist. Hofmus. 2: 63. 1887 syn. sec. Marhold (2011)

= *Dianthusjuratensis* Jord.﻿, Icon. Fl. Eur. 3: 32. 1903 syn. sec. WFO 2018

= Dianthussylvestrisf.albiflorus Micevski﻿ in Prilozi Oddel. Biol. Med. Nauki Makedonska Akad. Nauk. Umet. 8: 44. 1987 [“1990”] syn. sec. WFO 2018

= Dianthussylvestrisvar.alpestris Micevski﻿ in Prilozi Oddel. Biol. Med. Nauki Makedonska Akad. Nauk. Umet. 8: 42. 1987 [“1990”] syn. sec. WFO 2018

**Dianthussylvestrissubsp.alboroseus F.K.Mey.﻿ in Haussknechtia, Beih. 15: 53. 2011.** Sec. Meyer (2011)

**Dianthussylvestrissubsp.aristidis (Batt.) Greuter & Burdet﻿ in Willdenowia 12: 187. 1982.** Sec. Marhold (2011)

≡ *Dianthusaristidis* Batt.﻿, Fl. Algérie Dicot.(App. 2): v. 1888 syn. sec. Marhold (2011) ≡ Dianthuscaryophyllussubsp.aristidis (Batt.) Maire﻿, Fl. Afrique N. 10: 320. 1963 syn. sec. Kew WCVP (2019)

**Dianthussylvestrissubsp.bertisceus Rech.f.﻿ in Repert. Spec. Nov. Regni Veg. 38: 150. 1935.** Sec. Marhold (2011)

≡ *Dianthusbertisceus* (Rech.f.) E.Mayer & Trpin﻿ in Biol. Vestn. 13: 57. 1965 syn. sec. Marhold (2011)

**Dianthussylvestrissubsp.kozjakensis Micevski﻿ in Prilozi Oddel. Biol. Med. Nauki Makedonska Akad. Nauk. Umet. 8: 43. 1987 [“1990”].** Sec. Marhold (2011)

**Dianthussylvestrissubsp.longibracteatus (Maire) Greuter & Burdet in Willdenowia 12: 187. 1982.** Sec. Marhold (2011)

≡ Dianthuscaryophyllussubsp.longibracteatus Maire﻿ in Bull. Soc. His. Nat. Afrique N. 19: 33. 1928 syn. sec. Marhold (2011)

= Dianthuscaryophyllusvar.mogadorensis Maire﻿ in Bull. Soc. His. Nat. Afrique N. 20: 16. 1929 syn. sec. African Plant Database (version 3.4.0)

= Dianthuscaryophyllusvar.volubilitanus Maire﻿, Cat. Pl. Maroc 2: 238. 1932 syn. sec. WFO 2018 ≡ Dianthuscaryophyllusf.mogadorensis (Maire) Maire﻿, Fl. Afr. Nord 10: 319. 1963 syn. sec. WFO 2018

**Dianthussylvestrissubsp.longicaulis (Ten.) Greuter & Burdet﻿ in Willdenowia 12: 187. 1982.** Sec. Dimopoulos et al. (2013)

≡ *Dianthuslongicaulis* Ten.﻿, Cat. Hort. Neapol. 1813 App. 2: 77. 1819 syn. sec. Dimopoulos et al. (2013) ≡ Dianthuscaryophyllusvar.longicaulis (Ten.) Trevir.﻿, Index Seminum (WROCL, Wratislaviensi) 1821: 1. 1821 syn. sec. this publication ≡ Dianthuscaryophyllussubsp.longicaulis (Ten.) Arcang.﻿, Comp. Fl. Ital. ed. 2: 306. 1894 syn. sec. Dimopoulos et al. (2013)

= *Dianthusvirgineus* Gren. & Godr.﻿ in Bot. Mag. 42: t. 1740. 1815 syn. sec. Dimopoulos et al. (2013)

= Dianthuscaryophyllusvar.tenuifolius Moris﻿, Fl. Sardoa 1: 231. 1837 syn. sec. Dimopoulos et al. (2013) ≡ Dianthussiculussubsp.tenuifolius (Moris) Arrigoni﻿ in Parlatorea 7: 20. 2005 syn. sec. Dimopoulos et al. (2013)

= *Dianthusscheuchzeri* Jord.﻿, Mém. Acad. Sci. Lyon, Sect. Sci. 1: 241. 1851 syn. sec. Dimopoulos et al. (2013)

= *Dianthuscollivagus* Jord.﻿, Annot. Fl. France Allemange: 46. 1855 syn. sec. Dimopoulos et al. (2013)

= *Dianthusgodronianus* Jord.﻿, Annot. Fl. France Allemange: 45. 1855 syn. sec. Dimopoulos et al. (2013) ≡ Dianthuscaryophyllussubsp.godronianus (Jord.) P.Martin﻿ in Bull. Soc. Échange Pl. Vasc. Eur. Occid. Bassin Médit. 19: 93. 1984 syn. sec. Dimopoulos et al. (2013) ≡ Dianthussylvestrisvar.godronianus (Jord.) Kerguélen﻿ in Lejeunia 120: 81. 1987 syn. sec. Dimopoulos et al. (2013)

**Dianthussylvestrissubsp.nodosus (Tausch) Hayek﻿ in Repert. Spec. Nov. Regni Veg. Beih. 30(1): 247. 1924.** Sec. Marhold (2011)

≡ *Dianthusnodosus* Tausch﻿, Syll. Pl. Nov. 2: 243. 1828 syn. sec. Marhold (2011)

**Dianthussylvestrissubsp.sylvestris**﻿

**Dianthussylvestrissubsp.tergestinus (Rchb.) Hayek﻿ in Repert. Spec. Nov. Regni Veg. Beih. 30(1): 247. 1924.** Sec. Marhold (2011)

≡ Dianthusvirgineusvar.tergestinus Rchb.﻿, Icon. Fl. Germ. Helv. 6: 47, t. 5049b. 1844 syn. sec. Marhold (2011) ≡ *Dianthustergestinus* (Rchb.) A.Kern.﻿, Sched. Fl. Exs. Austro-Hung.: no. 545. 1882 syn. sec. Marhold (2011) ≡ Dianthuscaryophyllusvar.tergestinus (Rchb.) Tanfani﻿, Fl. Ital. 9: 283. 1892 syn. sec. this publication

***Dianthusszowitisianus* Boiss.﻿, Fl. Orient. 1: 503. 1867.** Sec. Rechinger (1988)

= Dianthuspallensvar.oxylepis Boiss.﻿, Fl. Orient. 1: 485. 1867 syn. sec. Rechinger (1988)

***Dianthustabrisianus* Bien. ex Boiss.﻿, Fl. Orient. 1: 496. 1867.** Sec. Rechinger (1988)

= *Dianthuspachypetalus* Stapf﻿ in Denkschr. Kaiserl. Akad. Wiss., Wien. Math.-Naturwiss. Kl. 51: 278. 1886 syn. sec. Rechinger (1988)

**Dianthustabrisianusvar.coloratus (Bornm.) Rech.f.﻿, Fl. Iran. 163: 147. 1988.** Sec. Rechinger (1988)

≡ Dianthuspachypetalusvar.coloratus Bornm.﻿ in Verh. K. K. Zool.-Bot. Ges. Wien 60: 80. 1910 syn. sec. Rechinger (1988) ≡ *Dianthuscoloratus* (Bornm.) Hand.-Mazz.﻿, Ann. K. K. Naturhist. Hofmus. 26: 153. 1912 syn. sec. Rechinger (1988)

**Dianthustabrisianusvar.tabrisianus**﻿

***Dianthustakhtajanii* Nersesian﻿ in Takhtajania 1: 47. 2011.** Sec. Kuzmina & Nersesyan (2012)

***Dianthustalyschensis* Boiss. & Buhse﻿ in Nouv. Mém. Soc. Imp. Naturalistes Moscou 12: 34. 1860.** Sec. Kuzmina & Nersesyan (2012)

***Dianthustarentinus* Lacaita﻿ in Nuovo Giorn. Bot. Ital. n.s., 18: 511. 1911.** Sec. Bacchetta et al. (2010)

= Dianthussylvestrisvar.garganicus Ten.﻿, Fl. Napol. 2: 208. 1830 syn. sec. Bacchetta et al. (2010) ≡ Dianthuscaryophyllussubsp.garganicus (Ten.) Grande﻿ in Boll. Soc. Bot. Ital. 1912: 178. 1912 syn. sec. Bacchetta et al. (2010) ≡ Dianthuscaryophyllusvar.garganicus (Ten.) Fiori﻿, Nuov. Fl. Italia 1: 512. 1924 syn. sec. Bacchetta et al. (2010) ≡ Dianthussylvestrissubsp.garganicus (Ten.) Pignatti﻿ in Giorn. Bot. Ital. 107: 211. 1973 syn. sec. Bacchetta et al. (2010) ≡ *Dianthusgarganicus* (Ten.) Brullo﻿ in Braun-Blanquetia 2: 31. 1988 syn. sec. Bacchetta et al. (2010)

***Dianthustenuiflorus* Griseb.﻿, Spic. Fl. Rumel. 1: 189. 1843.** Sec. Dimopoulos et al. (2013)

≡ Dianthuscorymbosussubsp.tenuiflorus (Griseb.) Trinajstić﻿ in Suppl. Fl. Anal. Jugosl. 5: 746. 1979 syn. sec. Dimopoulos et al. (2013)

***Dianthusthunbergii* S.S.Hooper﻿ in Hookers Icon. Pl. 37: 38. 1959.** Sec. African Plant Database (version 3.4.0)

= *Dianthushirtus* Vill.﻿, Syst. Pl. Eur. 1: 41. 1785 syn. sec. WFO 2018

= *Dianthusscaber* Chaix﻿, Hist. Pl. Dauphiné 1: 331. 1786 syn. sec. WFO 2018

= *Dianthusscaber* Thunb.﻿, Prodr. Pl. Cap. 1: 81. 1794 syn. sec. African Plant Database (version 3.4.0)

= *Dianthusprostratus* Eckl. & Zeyh.﻿, Enum. Pl. Afric. Austral. 1: 32. 1835 syn. sec. WFO 2018

= *Dianthusramentaceus* Fenzl ex Sond.﻿, Fl. Cap. 1: 122. 1860 syn. sec. WFO 2018

**Dianthusthunbergiif.maritimus S.S.Hooper﻿, Hooker’s Icon. Pl. 7 [1]: 38–40. 1959.** Sec. African Plant Database (version 3.4.0)

**Dianthusthunbergiif.thunbergii**﻿

***Dianthustianschanicus* Schischk.﻿, Fl. URSS 6: 897. 1936.** Sec. Czerepanov (1995)

***Dianthustlaratensis* Husseinov﻿ in Bot. Zhurn. (Moscow & Leningrad) 75: 234. 1990.** Sec. Kuzmina & Nersesyan (2012)

***Dianthustoletanus* Boiss. & Reut.﻿, Diagn. Pl. Nov. Hisp.: 7. 1842.** Sec. Bernal et al. (1990)

≡ Dianthusscabersubsp.toletanus (Boiss. & Reut.) Tutin﻿ in Feddes Repert. Spec. Nov. Regni Veg. 68: 190. 1963 syn. sec. Bernal et al. (1990)

***Dianthustranscaucasicus* Schischk.﻿ in Izv. Tomsk. Gosud. Univ. 80: 452. 1929.** Sec. Nersesyan (2011)

***Dianthustransvaalensis* Burtt Davy﻿ in Bull. Misc. Inform. Kew 1922: 215. 1922.** Sec. African Plant Database (version 3.4.0)

= Dianthusscabervar.graminifolius Fenzl ex Szyszyl.﻿ syn. sec. African Plant Database (version 3.4.0)

***Dianthustrifasciculatus* Kit. ex Schult.﻿, Oestr. Fl. ed. 2, 1: 654. 1814.** Sec. Marhold (2011)

= *Dianthuslancifolius* Tausch﻿ in Flora 14: 215. 1831 syn. sec. WFO 2018

= *Dianthusdanubialis* Griseb. & Schenk﻿ in Arch. Naturgesch. 18(1): 301. 1852 syn. sec. WFO 2018

= *Dianthusheptaneurus* Griseb. & Schenk﻿ in Arch. Naturgesch. 18(1): 302. 1852 syn. sec. WFO 2018

= *Dianthusbiternatus* Schur﻿ in Verh. Mitth. Siebenbürg. Vereins Naturwiss. Hermannstadt 4: 11. 1853 syn. sec. WFO 2018

= *Dianthustranssylvanicus* Schur﻿ in Verh. Mitth. Siebenbürg. Vereins Naturwiss. Hermannstadt 5: 82. 1854 syn. sec. WFO 2018

= *Dianthuspruinosus*Janka﻿, Index Seminum (WU) 1858: 4. 1858 syn. sec. WFO 2018

= *Dianthusbanaticus* Kit.﻿ in Linnaea 32: 527. 1863 syn. sec. WFO 2018

**Dianthustrifasciculatussubsp.parviflorus Stoj. & Acht.﻿, Sborn. Blghar. Akad. Nauk 29: 40. 1935.** Sec. Marhold (2011)

= Dianthustrifasciculatusvar.deserti Prodán﻿ syn. sec. WFO 2018 ≡ *Dianthusdeserti* (Prodán) Prodán﻿, Fl. Reipubl. Popul. Roman. 2: 232. 1953 syn. sec. Marhold (2011) ≡ Dianthustrifasciculatussubsp.deserti (Prodán) Tutin﻿ in Feddes Repert. Spec. Nov. Regni Veg. 68: 190. 1963 syn. sec. Marhold (2011)

**Dianthustrifasciculatussubsp.pseudobarbatus (Schmalh.) Jalas﻿ in Ann. Bot. Fenn. 22: 219. 1985.** Sec. Marhold (2011)

≡ Dianthuschinensissubsp.pseudobarbatus Schmalh.﻿, Fl. Sredn. Yuzhn. Rossii 1: 125. 1895 syn. sec. Marhold (2011) ≡ *Dianthuspseudobarbatus* (Schmalh.) Besser ex Klok.﻿, Fl. S.S.S.R. 4: 609. 1952 syn. sec. Marhold (2011)

= *Dianthuseuponticus* Zapał.﻿, Consp. Fl. Gallic. Crit. 3: 141. 1911 syn. sec. Marhold (2011)

**Dianthustrifasciculatussubsp.trifasciculatus**﻿

***Dianthustripunctatus* Sm.﻿, Fl. Graec. Prodr. 1(2): 286. 1809.** Sec. Dimopoulos et al. (2013)

= *Dianthusbarati* Duval-Jouve﻿ in Bull. Soc. Bot. France 2: 350. 1855 syn. sec. WFO 2018

***Dianthustunicoides* Madhani & Heubl﻿ in Taxon 67(1): 103. 2018.** Sec. Madhani et al. (2018)

≡ *Tunicasibthorpii* Boiss.﻿, Diagn. Pl. Orient., ser. 1, 8: 61. 1849, nom. illeg. syn. sec. Madhani et al. (2018) ≡ *Gypsophilaarmerioides* Ser.﻿ ex DC.﻿, Prodr. 1: 353. 1824 syn. sec. Madhani et al. (2018) ≡ *Tunicaarmerioides* (Ser. ex DC.) Halácsy﻿, Consp. Fl. Graec. 1: 194. 1900 syn. sec. Madhani et al. (2018) ≡ *Petrorhagiaarmerioides* (Ser. ex DC.) P.W.Ball & Heywood﻿ in Bull. Brit. Mus. (Nat. Hist.), Bot. 3: 139. 1964 syn. sec. Madhani et al. (2018) ≡ *Fiedleriaarmerioides* (Ser. ex DC.) Ovcz.﻿, Fl. Tadzhikskoi S.S.R. 3: 608. 1968 syn. sec. Madhani et al. (2018).

***Dianthusturkestanicus* Preobr.﻿ in Izv. Imp. Bot. Sada Petra Velikago 15: 366. 1915.** Sec. Czerepanov (1995)

≡ Dianthusversicolorsubsp.turkestanicus (Preobr.) Kozhevn.﻿ in Novosti Sist. Vyssh. Rast. 22: 113. 1985 syn. sec. Czerepanov (1995)

= *Dianthusalatavicus* Popov﻿ in Byull. Moskovsk. Obshch. Isp. Prir., Otd. Biol. n.s., 47: 86. 1938 syn. sec. Czerepanov (1995)

***Dianthustymphresteus* (Boiss. & Spruner) Heldr. & Sart. ex Boiss.﻿, Diagn. Pl. Orient. ser. 2, 6: 27. 1859.** Sec. Dimopoulos et al. (2013)

≡ Dianthusviscidusvar.tymphresteus Boiss. & Spruner﻿ in Boissier, Diagn. Pl. Orient., ser. 1, 8: 64. 1849 syn. sec. WFO 2018

***Dianthusucarii* Hamzaoğlu & Koç﻿ in Turkish J. Bot. 41: 487. 2017.** Sec. Hamzaoğlu et al. (2017)

***Dianthusugamicus* Vved.﻿ in Bot. Mater. Gerb. Bot. Inst. Uzbekistansk. Fil. Akad. Nauk S.S.S.R. 3: 10. 1941.** Sec. Czerepanov (1995)

***Dianthusuniflorus* Forssk.﻿, Fl. Aegypt.-Arab.: cxi. 1775.** Sec. WFO 2018

***Dianthusuralensis* Korsh.﻿ in Trudy Glavn. Bot. Sada 43: 310. 1930.** Sec. Czerepanov (1995)

***Dianthusurumoffii* Stoj. & Acht.﻿, Fl. Bulg. ed. 2: 364. 1933.** Sec. WFO 2018

***Dianthusuzbekistanicus* Lincz.﻿, Fl. Uzbekist. 2: 526. 1953.** Sec. Czerepanov (1995)

***Dianthusvanensis* Behçet & Ilçim﻿ in Turkish J. Bot. 37: 219. 2013.** Sec. İlçim et al. (2013)

***Dianthusvarankii* Hamzaoğlu & Koç﻿ in KSÜ Tarim Doga Derg. 21: 546. 2018.** Sec. Hamzaoğlu et al. (2018)

**Dianthus×varians Rouy & Foucaud﻿, Fl. France 3: 186. 1896.** Sec. Bernal et al. (1990)

≡ Dianthus×saxatilis nothosubsp. *varians* (Rouy & Foucaud) M.Bernal, Laínz & Muñoz Garm.﻿ in Anales Jard. Bot. Madrid 45: 367. 1988 syn. sec. Bernal et al. (1990)

***Dianthusvigoi* M.Laínz﻿ in Anales Jard. Bot. Madrid 42: 551. 1985 [“1985”].** Sec. Bernal et al. (1990)

≡ Dianthusseguierisubsp.vigoi (M.Laínz) O.Bolòs﻿, Fl. Països Catalans 2: 757. 1990 syn. sec. Marhold (2011)

***Dianthusvirgatus* Pasq.﻿ in Ann. Accad. Aspir. Naturalisti III, 1: 28. 1861.** Sec. Bacchetta et al. (2010)

≡ Dianthuscaryophyllussubsp.virgatus (Pasq.) Arcang.﻿, Comp. Fl. Ital. ed. 2: 306. 1894 syn. sec. Bacchetta et al. (2010)

***Dianthusvirgineus* L., Sp. Pl.: 412. 1753.** Sec. Domina et al. (2021)

≡ *Tunicavirginea* (L.) Scop.﻿, Fl. Carniol., ed. 2, 1: 302. 1771 syn. sec. Domina et al. (2021); ≡ Dianthuscaryophyllusvar.virgineus (L.) Fiori﻿, Fl. Italia 1(2): 379. 1898 syn. sec. Domina et al. (2021) ≡ *Dianthusrupestris* Lam.﻿, Fl. Franç. 2: 536. 1779, nom. illeg. syn. sec. Domina et al. (2021) ≡ *Dianthusscheuchzeri* Rchb.﻿, Fl. Germ. Excurs.: 811. 1832 syn. sec. Domina et al. (2021)

***Dianthusviridescens* Clementi﻿ in Atti Riunione Sci. Ital. 3: 520. 1841.** Sec. Marhold (2011)

***Dianthusviscidus* Bory & Chaub.﻿, Nouv. Fl. Pélop.: 26. 1838.** Sec. Dimopoulos et al. (2013)

= *Dianthusolympicus* Boiss.﻿, Diagn. Pl. Orient. 1: 19. 1843 syn. sec. Kew WCVP (2019)

= *Dianthusgrisebachii* Boiss.﻿, Diagn. Pl. Orient., ser. 2, 1: 62. 1854 syn. sec. WFO 2018

= *Dianthusparnassicus* Boiss. & Heldr.﻿ in Boissier, Diagn. Pl. Orient., ser. 2, 1: 64. 1854 syn. sec. WFO 2018

= *Dianthusheldreichii* Bornm.﻿ in Bot. Jahrb. Syst. 59: 393. 1924 syn. sec. WFO 2018

= Dianthusviscidussubsp.elatior (Halácsy) Hayek﻿ in Repert. Spec. Nov. Regni Veg. Beih. 30(1): 227. 1924 syn. sec. Dimopoulos et al. (2013)

= Dianthusviscidussubsp.grisebachii (Boiss.) Hayek﻿ in Repert. Spec. Nov. Regni Veg. Beih. 1: 226. 1924 syn. sec. Dimopoulos et al. (2013)

= Dianthusviscidusvar.glandulosus Micevski﻿ in Prilozi Oddel. Biol. Med. Nauki Makedonska Akad. Nauk. Umet. 8: 44. 1987 [“1990”] syn. sec. WFO 2018

***Dianthusvladimirii* Galushko﻿ in Novosti Sist. Vyssh. Rast. 11: 298. 1974.** Sec. Kuzmina & Nersesyan (2012)

***Dianthusvolgicus* Juz.﻿ in Bot. Mater. Gerb. Bot. Inst. Komarova Akad. Nauk S.S.S.R. 13: 73. 1950.** Sec. Czerepanov (1995)

***Dianthusvulturius* Guss. & Ten.﻿, Index Seminum (NAP) 1837: 3. 1837.** Sec. Marhold (2011)

≡ Dianthuscarthusianorumvar.vulturius (Guss. & Ten.) Tanfani﻿, Fl. Ital. 9: 253. 1892 syn. sec. Kew WCVP (2019) ≡ Dianthusbalbisiisubsp.vulturius (Guss. & Ten.) Maire﻿ in Bull. Soc. His. Nat. Afrique N. 23: 169. 1932 syn. sec. Marhold (2011) ≡ Dianthusferrugineussubsp.vulturius (Guss. & Ten.) Tutin﻿ in Feddes Repert. Spec. Nov. Regni Veg. 68: 191. 1963 syn. sec. Marhold (2011)

**Dianthusvulturiussubsp.aspromontanus Brullo, Scelsi & Spamp.﻿ in Portugaliae Acta Biol., Sér. B, Sist. 19: 310. 2000.** Sec. Brullo et al. (2000)

**Dianthusvulturiussubsp.vulturius**﻿

**Dianthus×warionii Timb.-Lagr. in Bull. Soc. Agric. Sci. Litt. Pyrén.-Orient. 25: 20. 1881.** Sec. Bernal et al. (1990)

≡ *Dianthusrichteri* nothovar. *warionii* (Timb.-Lagr.) Rouy & Foucaud﻿, Fl. France 3: 183. 1896 syn. sec. Bernal et al. (1990)

= Dianthus×richteri Rouy & Foucaud﻿, Fl. France 3: 182. 1896 syn. sec. Bernal et al. (1990)

***Dianthusworoschilovii* Barkalov & Prob.﻿, Fl. Ross. Dalnego Vostoka: 76. 2006.** Sec. Barkalov & Probatova (2006)

≡ Dianthuschinensissubsp.reflexus Vorosch.﻿ in Byull. Moskovsk. Obshch. Isp. Prir., Otd. Biol. n.s., 83(5): 116. 1978 syn. sec. Barkalov & Probatova (2006)

***Dianthusxylorrhizus* Boiss. & Heldr.﻿ in Boissier, Diagn. Pl. Orient., ser. 1, 8: 67. 1849.** Sec. Dimopoulos et al. (2013)

***Dianthuszangezuricus* Nersesian﻿ in Novosti Sist. Vyssh. Rast. 42: 115. 2011.** Sec. Kuzmina & Nersesyan (2012)

***Dianthuszederbaueri* Vierh.﻿ in Ann. K. K. Naturhist. Hofmus. 20: 391. 1905.** Sec. Marhold (2011)

***Dianthuszeyheri* Sond.﻿, Fl. Cap. 1: 124. 1860.** Sec. African Plant Database (version 3.4.0)

= *Dianthusalbens* Turcz.﻿ in Bull. Soc. Imp. Naturalistes Moscou 27(2): 369. 1854 syn. sec. WFO 2018

= *Dianthusserpae* Ficalho & Hiern﻿ in Trans. Linn. Soc. London, Bot. 2: 17. 1881 syn. sec. African Plant Database (version 3.4.0)

= *Dianthusmecistocalyx* F.N.Williams﻿ in J. Bot. 27: 199. 1889 syn. sec. African Plant Database (version 3.4.0)

**Dianthuszeyherisubsp.natalensis S.S.Hooper﻿ in Hookers Icon. Pl. 37: 48. 1959.** Sec. African Plant Database (version 3.4.0)

= *Dianthuscolensoi* F.N.Williams﻿ in J. Bot. 23: 344. 1885 syn. sec. African Plant Database (version 3.4.0)

**Dianthuszeyherisubsp.zeyheri**﻿

***Dianthuszonatus* Fenzl﻿, Pug. Pl. Nov. Syr.: 11. 1842.** Sec. Dimopoulos et al. (2013)

= *Dianthushypochlorus* Boiss. & Heldr.﻿ in Boissier, Diagn. Pl. Orient., ser. 1, 8: 67. 1849 syn. sec. WFO 2018

### ﻿Hybrids

**Dianthus×ambrozy-migazzianus Boros﻿ in Magyar Bot. Lapok 23: 33. 1924 [“1925”].** Sec. WFO 2018

**Dianthus×arvernensis Rouy & Foucaud﻿, Fl. France 3: 185. 1896.** Sec. WFO 2018

**Dianthus×brivatensis Blanch.﻿, Jardin 6: 174. 1892.** Sec. WFO 2018

**Dianthus×burciae Péterfi & Gürtler﻿ in Magyar Bot. Lapok 15: 20. 1916.** Sec. WFO 2018

**Dianthus×calalpinus Farrer﻿ in Engl. Rock Gard. 1: 283. 1919**

**Dianthus×callizonoides Sünd.﻿ in Allg. Bot. Z. Syst. 12: 91. 1906.** Sec. Kew WCVP (2019)

**Dianthus×courtoisii Rchb.﻿, Fl. Germ. Excurs.: 806. 1832.** Sec. WFO 2018

= Dianthus×wolfii Vetter﻿ in Bull. Murith. Soc. Valais. Sci. Nat. 11: 132. 1883 syn. sec. Kew WCVP (2019)

**Dianthus×digeneus Rouy & Foucaud﻿, Fl. France 3: 184. 1896.** Sec. WFO 2018

**Dianthus×ebneri Heimerl﻿, Fl. Brixen: 105. 1911.** Sec. WFO 2018

**Dianthus×gabrielae Domin﻿ in Vestn. Král. Ceské Spolecn. Nauk. Tr. Mat.-Prír. 13: 4. 1927 [“1926”].** Sec. WFO 2018

**Dianthus×genersichii Györffy﻿ in Magyar Bot. Lapok 23: 70. 1924 [“1925”].** Sec. WFO 2018

**Dianthus×josephinae Font Quer﻿ in Cavanillesia 1: 34. 1928.** Sec. WFO 2018

**Dianthus×julii-wolfii Marton﻿ in Magyar Bot. Lapok 15: 19. 1916.** Sec. WFO 2018

**Dianthus×krasanii A.Kern. ex Dalla Torre & Sarnth.﻿, Fl. Tirol 6(2): 209. 1909.** Sec. WFO 2018

**Dianthus×lacinulatus Zapał.﻿, Consp. Fl. Gallic. Crit. 3: 161. 1911.** Sec. WFO 2018

**Dianthus×lamyi Rouy & Foucaud﻿, Fl. France 3: 176. 1896.** Sec. WFO 2018

**Dianthus×laucheanus Bolle﻿ ex Bolle & Asch.﻿ in Verh. Bot. Vereins Prov. Brandenburg 33: 102. 1892.** Sec. WFO 2018

**Dianthus×loretii Rouy & Foucaud﻿, Fl. France 3: 176. 1896.** Sec. WFO 2018

**Dianthus×mammingiorum Murr﻿ in Allg. Bot. Z. Syst. 13: 23. 1907.** Sec. WFO 2018

**Dianthus×nigritus Hirahata & Kitam.﻿ in Acta Phytotax. Geobot. 20: 205. 1962.** Sec. WFO 2018

**Dianthus×paradoxus Keller ex Schröt.﻿ in Ber. Schweiz. Bot. Ges. 14: 117. 1905.** Sec. POWO. Plants of the World Online. Facilitated by the Royal Botanic Gardens, Kew.

**Dianthus×ponsi Rouy & Foucaud﻿, Fl. France 3: 184. 1896.** Sec. WFO 2018

**Dianthus×pritchardii Farrer, Engl. Rock Gard. 1: 300. 1919**

**Dianthus×saxatilis F.W.Schmidt﻿ in Neuere Abh. Königl. Böhm. Ges. Wiss. 1: 28. 1790.** Sec. WFO 2018

**Dianthus×zarencznianus Zapał.﻿, Consp. Fl. Gallic. Crit. 3: 149. 1911.** Sec. WFO 2018

### ﻿Unresolved names

***Dianthusacuminatus* F.W.Williams﻿ in J. Bot. 23: 347. 1885.** Sec. WFO 2018

***Dianthusadulterinus* Sweet﻿, Hort. Brit. ed. 2: 50. 1830.** Sec. WFO 2018

***Dianthusambiguus* Pančić﻿, Fl. Serbiae: 178. 1874.** Sec. WFO 2018

***Dianthusambiguus* Pančić﻿ ex F.N.Williams﻿ in J. Bot. 23: 342. 1885.** Sec. WFO 2018

***Dianthusandersonii* F.N.Williams﻿ in J. Bot. 23: 346. 1885.** Sec. WFO 2018

***Dianthusaragonensis* Timb.-Lagr.﻿, Mém. Acad. Sci. Toulouse VI, 5: 242. 1867.** Sec. WFO 2018

**Dianthusarenariusvar.gigas Novák﻿.** Sec. WFO 2018

≡ Dianthusarenariussubsp.gigas (Novák) Soó﻿ in Feddes Repert. 83: 161. 1972 syn. sec. WFO 2018

***Dianthusarmerioides* Raf.﻿ in J. Bot. Agric. 4: 269. 1814.** Sec. WFO 2018

***Dianthusattenuatus* proles *maritimus* Rouy﻿.** Sec. Kew WCVP (2019)

***Dianthusbanaticiformis* Prodán﻿ in Bul. Soc. Sti. Cluj 10: 152. 1948.** Sec. WFO 2018

***Dianthusbasalticus* (Domin) Fritsch﻿, Exkursionsfl. Oesterreich ed. 2: 215. 1909.** Sec. WFO 2018

≡ Dianthuscarthusianorumvar.basalticus Domin﻿ syn. sec. this publication

***Dianthusbehriorum* Bornm.﻿ in Repert. Spec. Nov. Regni Veg. 41: 188. 1936.** Sec. WFO 2018

***Dianthusbolfae* Sennen﻿ in Bol. Soc. Ibér. Ci. Nat. 25: 149. 1926.** Sec. WFO 2018

***Dianthusbornmuelleri* Hausskn.﻿ in Mitt. Geogr. Ges. (Thüringen) Jena 9: 16. 1891.** Sec. WFO 2018

***Dianthusborzaeanus* Prodán﻿ in Bul. Inform. Grad. Bot. Univ. Cluj 5: 41. 1925.** Sec. WFO 2018

***Dianthusbottemeri* Bouchard﻿ in Bull. Soc. Bot. France 97: 220. 1951.** Sec. WFO 2018

***Dianthusbrachyanthus* Gren. & Godr.﻿, Fl. France 1: 235. 1847.** Sec. WFO 2018

***Dianthusbrachycarpus* Velen.﻿ in Abh. Königl. Böhm. Ges. Wiss. 7(Folge 1): 9. 1886.** Sec. WFO 2018

***Dianthusbrevilimbis* Boiss. ex Trautv.﻿ in Trudy Imp. S.-Peterburgsk. Bot. Sada 2: 506. 1873.** Sec. WFO 2018

***Dianthusbrevior* Gand.﻿, Contr. Fl. Terr. Slav. Merid. 1: 5. 1883.** Sec. WFO 2018

***Dianthusbrevistylus* Timb.-Lagr. & Jeanb.﻿ in Bull. Soc. Agric. Sci. Litt. Pyrén.-Orient. 25: 24. 1881.** Sec. WFO 2018

***Dianthusbucovinensis* Prodán & Morariu﻿ in Bul. Sti. Acad. Republ. Populare Romîne, Sect. Biol. Sti. Agric. Ser. Bot., 9: 301. 1957.** Sec. WFO 2018

***Dianthusbuergeri* Miq.﻿ in Ann. Mus. Bot. Lugduno-Batavi 2: 77. 1865.** Sec. WFO 2018

***Dianthuscallichrous* Jord.﻿, Icon. Fl. Eur. 3: 30. 1903.** Sec. WFO 2018

***Dianthuscallizonoides* Sünd.﻿ in Allg. Bot. Z. Syst. 12: 91. 1906.** Sec. WFO 2018

***Dianthuscalverti* Boiss.﻿, Asie Min., Bot. 1: 223. 1860.** Sec. WFO 2018

***Dianthuscamboi* Sennen﻿, Diagn. Nouv.: 42. 1936.** Sec. WFO 2018

***Dianthuscamusii* Sennen﻿, Diagn. Nouv.: 9. 1936.** Sec. WFO 2018

***Dianthuscarneus* Jord.﻿, Icon. Fl. Eur. 3: 25. 1903.** Sec. WFO 2018

***Dianthuscarpaticus* Woloszack﻿.** [publication] Sec. WFO 2018

***Dianthuscaucasicus* Spreng.﻿, Adumbr. Pl. Hort. Hal.: 23. 1806.** Sec. WFO 2018

***Dianthuschlorostomus* Jord.﻿, Icon. Fl. Eur. 3: 31. 1903.** Sec. WFO 2018

***Dianthuscollincola* Spreng.﻿ in Bot. Gart. Halle Nachtr. 1: 20. 1801.** Sec. WFO 2018

***Dianthuscompanyoi* Sennen﻿ in Bol. Soc. Ibér. Ci. Nat. 25: 208. 1926 [“1927”].** Sec. WFO 2018

***Dianthuscorymbosus* Andrz.﻿ in Trudy Komiss. Vys. Uchr., Kiev 1: 18. 1860.** Sec. WFO 2018

***Dianthuscostei* Sennen﻿ in Bull. Acad. Int. Geogr. Bot. 24: 237. 1914.** Sec. WFO 2018

***Dianthuscruentus* Fisch. ex Planch.﻿, Fl. Serres Jard. Eur. 5: t. 488. 1849.** Sec. WFO 2018

***Dianthuscurticeps* Borbás﻿ in Oesterr. Bot. Z. 40: 97. 1890.** Sec. WFO 2018

***Dianthusdecrescens* Borbás﻿ in Oesterr. Bot. Z. 27: 378. 1877.** Sec. WFO 2018

***Dianthusdeserti* Post﻿, Pl. Postianae 2: 6. 1891.** Sec. WFO 2018

***Dianthusdeserti* Kotschy﻿ in Sitzungsber. Kaiserl. Akad. Wiss., Math.-Naturwiss. Cl., Abt. 1, 52(1): 262. 1866.** Sec. WFO 2018

***Dianthusdissimilis* (Burnat) Landolt﻿, Fl. Indicativa: 268. 2010.** Sec. WFO 2018

***Dianthusdivaricatus* d’Urv.﻿, Mém. Soc. Linn. Paris 1: 302. 1822.** Sec. WFO 2018

***Dianthusdostalii* Novák﻿ in Repert. Spec. Nov. Regni Veg. 38: 304. 1935.** Sec. WFO 2018

***Dianthusegregius* Jord.﻿, Icon. Fl. Eur. 3: 23. 1903.** Sec. WFO 2018

***Dianthuselegans* M.Bieb. ex Rupr.﻿, Fl. Caucasi: 174. 1870.** Sec. WFO 2018

***Dianthuselongatus* C.A.Mey.﻿, Verz. Caucasus Pfl.: 211. 1849.** Sec. WFO 2018

***Dianthuserubescens* Roth﻿, Enum. Pl. Phaen. Germ. 1(2): 279. 1827.** Sec. WFO 2018

***Dianthusexilis* Posp.﻿, Fl. Oesterr. Küstenl. 1: 452. 1897.** Sec. WFO 2018

***Dianthusfallacinus* Rouy & Foucaud﻿, Fl. France 3: 190. 1896.** Sec. WFO 2018

***Dianthusfaurei* Arv.-Touv.﻿ in Bull. Soc. Dauphin. Échange Pl. 7: 263. 1880.** Sec. WFO 2018

***Dianthusfauriei* H.Lév. & Vaniot﻿ in Repert. Spec. Nov. Regni Veg. 7: 200. 1909.** Sec. WFO 2018

***Dianthusflabellatus* Jord.﻿, Icon. Fl. Eur. 3: 29. 1903.** Sec. WFO 2018

***Dianthusflahaultii* Braun-Blanq.﻿ in Annuaire Conserv. Jard. Bot. Genève 21: 32. 1919.** Sec. WFO 2018

***Dianthusflexicaulis* Jord.﻿, Icon. Fl. Eur. 3: 26. 1903.** Sec. WFO 2018

***Dianthusfloribundus* Sennen﻿ in Bull. Soc. Bot. France 73: 645. 1926 [“1927”].** Sec. WFO 2018

***Dianthusfoliosus* Turcz.﻿ in Bull. Soc. Imp. Naturalistes Moscou 5: 184. 1832.** Sec. WFO 2018

***Dianthusgalicicae* Micevski﻿ in Prilozi Oddel. Biol. Med. Nauki Makedonska Akad. Nauk. Umet. 8: 40. 1987 [“1990”].** Sec. WFO 2018

***Dianthusgelcenii* Gelcenis ex Sennen﻿ in Bull. Soc. Bot. France 73: 646. 1926 [“1927”].** Sec. WFO 2018

***Dianthusgizellae* Borbás﻿ in Oesterr. Bot. Z. 27: 378. 1877.** Sec. WFO 2018

***Dianthusglaucophyllus* Colla﻿ in Mem. Reale Accad. Sci. Torino 35: 187. 1831.** Sec. WFO 2018

***Dianthusgrandiflorus* Poir.﻿, Encycl. 4: 515. 1798.** Sec. WFO 2018

***Dianthusgremblichii* Asch.﻿ in Sitzungsber. Bot. Vereins Prov. Brandenburg 19: 28. 1877.** Sec. WFO 2018

***Dianthusgustavii* Sennen﻿, Diagn. Nouv.: 9. 1936.** Sec. WFO 2018

***Dianthushandelii* Hayek﻿ in Ann. K. K. Naturhist. Hofmus. 28: 157. 1914.** Sec. WFO 2018

***Dianthushanryi* Burnat﻿, Fl. Alpes Marit. 1: 223. 1892.** Sec. WFO 2018

***Dianthushazaricus* F.N.Williams﻿ in Bull. Herb. Boissier, sér. 2, 1: 892. 1901.** Sec. WFO 2018

***Dianthushelwigii* Asch.**﻿ [publication] Sec. WFO 2018

***Dianthushendersonianus* Paxton﻿ in Fl. Mag. (London) 14: 126. 1848.** Sec. WFO 2018

***Dianthusheterophyllus* Rouy & Foucaud﻿, Fl. France 3: 172. 1896.** Sec. WFO 2018

***Dianthushornemannii* Ser.﻿, Prodr. 1: 262. 1824.** Sec. WFO 2018

= *Dianthusgeminatus* Kit. ex Schult.﻿, Oestr. Fl. ed. 2, 1: 656. 1814 syn. sec. WFO 2018

***Dianthushuebneri* Seehaus﻿ in Verh. Bot. Vereins Prov. Brandenburg 33: 95. 1892.** Sec. WFO 2018

***Dianthushypoleucus* Jord.﻿, Icon. Fl. Eur. 3: 31. 1903.** Sec. WFO 2018

***Dianthusimpunctatus* Jord.﻿, Icon. Fl. Eur. 3: 29. 1903.** Sec. WFO 2018

***Dianthusinoxianus* Gallego﻿ in Lagascalia 14: 71. 1986.** Sec. WFO 2018

***Dianthusistriacus* Gand.﻿, Contr. Fl. Terr. Slav. Merid. 1: 5. 1883.** Sec. WFO 2018

***Dianthusitalicus* Jan ex Pasq.﻿, Index Seminum (NAP): 1866. 1866.** Sec. WFO 2018

***Dianthusjablanicensis* Micevski﻿ in Prilozi Oddel. Biol. Med. Nauki Makedonska Akad. Nauk. Umet. 8: 31. 1987 [“1990”].** Sec. WFO 2018

***Dianthusjacupicensis* (Košanin) Micevski﻿ in Prilozi Oddel. Biol. Med. Nauki Makedonska Akad. Nauk. Umet. 8: 41. 1987 [“1990”].** Sec. WFO 2018

***Dianthusjapigicum* Bianco & Brullo﻿.** Sec. WFO 2018

***Dianthusjugoslavicus* Micevski﻿ in Prilozi Oddel. Biol. Med. Nauki Makedonska Akad. Nauk. Umet. 8: 33. 1987 [“1990”].** Sec. WFO 2018

**Dianthus×laucheanus Bolle﻿ in Gartenflora 53: 393. 1904.** Sec. WFO 2018

***Dianthuslaxus* Tausch﻿, Hort. Canal. 2: t. 17. 1825.** Sec. WFO 2018

***Dianthuslereschii* (Burnat) Landolt﻿, Fl. Indicativa: 268. 2010.** Sec. WFO 2018

***Dianthuslevieri* Borbás﻿ in Oesterr. Bot. Z. 27: 231. 1877.** Sec. WFO 2018

***Dianthuslisae* Burnat﻿, Fl. Alpes Marit. 1: 233. 1892.** Sec. WFO 2018

***Dianthuslongicaulis* St.-Lag.﻿ in Ann. Soc. Bot. Lyon 7: 144. 1880.** Sec. WFO 2018

***Dianthuslongifolius* Jord.﻿, Icon. Fl. Eur. 3: 32. 1903.** Sec. WFO 2018

***Dianthusmacedonicus* Micevski﻿ in Prilozi Oddel. Biol. Med. Nauki Makedonska Akad. Nauk. Umet. 8: 35. 1987 [“1990”].** Sec. WFO 2018

***Dianthusmacrophyllus* Jord.﻿, Icon. Fl. Eur. 3: 30. 1903.** Sec. WFO 2018

***Dianthusmagyari* Dorsch﻿, Kert. Szemle 5: 241. 1933.** Sec. WFO 2018

***Dianthusmarginatus* Poir.﻿, Encycl. Suppl. 4: 131. 1816.** Sec. WFO 2018

***Dianthusmaris* Willd. ex B.S.Williams﻿ in Bot. J. Linn. Soc. 29(203): 415. 1893.** Sec. WFO 2018

***Dianthusmarisensis* Simonk.﻿ in Term. Füz. 9: 37. 1885.** Sec. WFO 2018

***Dianthusmaritimus* Jord.﻿, Icon. Fl. Eur. 3: 25. 1903.** Sec. WFO 2018

***Dianthusmassiliensis* Jord.﻿, Icon. Fl. Eur. 3: 25. 1903.** Sec. WFO 2018

***Dianthusmayeri* C.Presl﻿ in Abh. Königl. Böhm. Ges. Wiss. V, 3: 448. 1845.** Sec. WFO 2018

***Dianthusmedius* Besser ex Nyman﻿, Consp. Fl. Eur. 1: 103. 1878.** Sec. WFO 2018

***Dianthusmicrocheilus* B.S.Williams﻿ in Oesterr. Bot. Z. 41: 176. 1891.** Sec. WFO 2018

***Dianthusmikii* Reichardt﻿ in Verh. K. K. Zool.-Bot. Ges. Wien 17: 331. 1867.** Sec. WFO 2018

***Dianthusminimus* Javeiol﻿ in Curr. Sci. 42: 692. 1973.** Sec. WFO 2018

***Dianthusminor* Porta﻿ in Nuovo Giorn. Bot. Ital. 11: 278. 1879.** Sec. WFO 2018

***Dianthusmoldavicus* Grinț.﻿ in Bul. Inform. Grad. Bot. Univ. Cluj 4: 57. 1924.** Sec. WFO 2018

***Dianthusmonteuilii* Burnat﻿, Fl. Alpes Marit. 1: 221. 1891.** Sec. WFO 2018

***Dianthusmorrisii* Hance﻿ in London J. Bot. 7: 472. 1848.** Sec. WFO 2018

≡ *Tunicamorrisii* (Hance) Walp.﻿ in Ann. Bot. Syst. 2: 101. 1851 syn. sec. Kew WCVP (2019);

***Dianthusnevadensis* Sennen﻿, Diagn. Nouv.: 210. 1936.** Sec. WFO 2018

***Dianthusnicaeensis* Jord.﻿, Icon. Fl. Eur. 3: 30. 1903.** Sec. WFO 2018

***Dianthusnobilis* Anon.﻿ in Gartenflora 81: 94. 1932.** Sec. WFO 2018

***Dianthusnovakii* J.Sojak﻿, Čas. Nár. Mus., Odd. Přír. 148: 77. 1979 [“1980”].** Sec. WFO 2018

***Dianthusobtusifolius* Scheele﻿ in Flora 26: 428. 1843.** Sec. WFO 2018

***Dianthusoculatus* Boiss.﻿ in Ann. Sci. Nat., Bot. IV, 2: 245. 1854.** Sec. WFO 2018

**Dianthus×oenipontanus A.Kern.﻿ in Oesterr. Bot. Z. 15: 209. 1865.** Sec. WFO 2018

***Dianthusohridanus* Micevski﻿ in Prilozi Oddel. Biol. Med. Nauki Makedonska Akad. Nauk. Umet. 8: 34. 1987 [“1990”].** Sec. WFO 2018

***Dianthuspancicii* F.N.Williams﻿.** [publication] Sec. WFO 2018

***Dianthusparciflorus* Jord.﻿, Icon. Fl. Eur. 3: 28. 1903.** Sec. WFO 2018

***Dianthuspedemontanus* Rouy﻿, Fl. France 3: 188. 1896.** Sec. WFO 2018

***Dianthuspenrynae* Sweet﻿, Hort. Brit. ed. 2: 51. 1830.** Sec. WFO 2018

***Dianthuspetteri* Boiss.﻿, Diagn. Pl. Orient., ser. 2, 1: 66. 1854.** Sec. WFO 2018

***Dianthusplatyphyllus* Turcz.﻿ in Bull. Soc. Imp. Naturalistes Moscou 27(2): 368. 1854.** Sec. WFO 2018

***Dianthuspomeridianus* L.﻿, Sp. Pl., ed. 2 2: 1673. 1763.** Sec. WFO 2018

= *Dianthustricolor* Adams ex Ser.﻿, Prodr. 1: 360. 1824 syn. sec. WFO 2018

***Dianthusponticola* Bornm.﻿ in Bot. Jahrb. Syst. 59: 398. 1924.** Sec. POWO. Plants of the World Online. Facilitated by the Royal Botanic Gardens, Kew.

***Dianthusponticolus* Bornm.﻿ in Bot. Jahrb. Syst. 59: 398. 1924.** Sec. WFO 2018

***Dianthusportae* A.Kern. ex Huter﻿ in Oesterr. Bot. Z. 54: 339. 1904.** Sec. WFO 2018

***Dianthuspraevertens* B.S.Williams﻿ in Bot. J. Linn. Soc. 29(203): 408. 1893.** Sec. WFO 2018

***Dianthusprilepensis* Micevski﻿ in Prilozi Oddel. Biol. Med. Nauki Makedonska Akad. Nauk. Umet. 8: 39. 1987 [“1990”].** Sec. WFO 2018

***Dianthuspuberulus* F.N.Williams﻿ in J. Bot. 23: 344. 1885.** Sec. WFO 2018

***Dianthuspuberulus* Jord.﻿, Icon. Fl. Eur. 3: 31. 1903.** Sec. WFO 2018

***Dianthuspulchellus* Pers.﻿, Syn. Pl. 1: 495. 1805.** Sec. WFO 2018

***Dianthuspulverulentus* Wettst.﻿ in Sitzungsber. Kaiserl. Akad. Wiss., Math.-Naturwiss. Cl., Abt. 1, 98: 382. 1889 [“1896”].** Sec. WFO 2018

***Dianthuspumilus* Vahl﻿, Symb. Bot. 1: 32. 1790.** Sec. WFO 2018

***Dianthuspunctatus* Spreng.﻿, Neue Entdeck. Pflanzenk. 2: 169. 1821.** Sec. WFO 2018

***Dianthuspungens* Poir.﻿, Encycl. 4: 526. 1798.** Sec. WFO 2018

***Dianthuspurpureus* F.N.Williams﻿ in J. Bot. 23: 343. 1885.** Sec. WFO 2018

***Dianthuspusillus* F.W.Schmidt﻿ in Neuere Abh. Königl. Böhm. Ges. Wiss. 1: 29. 1791.** Sec. WFO 2018

***Dianthusquetierii* Carrière ex Herincq﻿, Hort. Franç. 1866: 43. 1866.** Sec. WFO 2018

***Dianthusramiger* Jord.﻿, Icon. Fl. Eur. 3: 28. 1903.** Sec. WFO 2018

***Dianthusrigidulus* Jord.﻿, Icon. Fl. Eur. 3: 27. 1903.** Sec. WFO 2018

***Dianthusrubicundus* Jord.﻿, Icon. Fl. Eur. 3: 24. 1903.** Sec. WFO 2018

***Dianthusruscinonensis* Foucaud & Gaut.﻿, Cat. Fl. Pyrénées-orientales: 108. 1898.** Sec. WFO 2018

***Dianthussallentii* Sennen﻿ in Bull. Acad. Int. Geogr. Bot. 24: 236. 1914.** Sec. WFO 2018

***Dianthusschlosseri* F.N.Williams﻿ in J. Bot. 23: 342. 1885.** Sec. WFO 2018

**Dianthus×seehausianus Asch. ex Seehaus﻿ in Verh. Bot. Vereins Prov. Brandenburg 34: 12. 1893.** Sec. WFO 2018

***Dianthusseguieri* Costa ex Willk. & Lange﻿, Prodr. Fl. Hispan. 3: 681. 1878.** Sec. WFO 2018

***Dianthusseisuimontanus* Masam.﻿ in Trans. Nat. Hist. Soc. Taiwan 31: 343. 1941.** Sec. WFO 2018

***Dianthusserrulatus* Schloss. & Vuk.﻿, Fl. Croat.: 321. 1869.** Sec. WFO 2018

***Dianthussilenoides* Poir.﻿, Encycl. 4: 514. 1798.** Sec. WFO 2018

***Dianthussinguliflorus* Jord.﻿, Icon. Fl. Eur. 3: 26. 1903.** Sec. WFO 2018

***Dianthussintenisii* Freyn﻿ in Oesterr. Bot. Z. 43: 376. 1893.** Sec. WFO 2018

***Dianthusskopjensis* Micevski﻿ in Prilozi Oddel. Biol. Med. Nauki Makedonska Akad. Nauk. Umet. 8: 36. 1987 [“1990”].** Sec. WFO 2018

***Dianthusslavonicus* B.S.Williams﻿ in Bot. J. Linn. Soc. 29(203): 380. 1893.** Sec. WFO 2018

***Dianthussouliei* Sennen﻿ in Bull. Soc. Bot. France 73: 646. 1926 [“1927”].** Sec. WFO 2018

***Dianthusspurius* A.Kern. ex Borbás﻿ in Rad Jugoslav. Akad. Znan. 36: 173. 1876.** Sec. WFO 2018

***Dianthussuavis* Willd.﻿, Enum. Pl., Suppl.: 24. 1814.** Sec. WFO 2018

***Dianthussubalbidus* Jord.﻿, Icon. Fl. Eur. 3: 24. 1903.** Sec. WFO 2018

***Dianthussubbarbatus* Schur﻿, Enum. Pl. Transsilv.: 92. 1866.** Sec. WFO 2018

***Dianthussubulatus* Timb.-Lagr.﻿ in Bull. Soc. Agric. Sci. Litt. Pyrén.-Orient. 25: 13. 1881.** Sec. WFO 2018

***Dianthussyriacus* Steud.﻿, Nomencl. Bot., ed. 2, 1: 501. 1840.** Sec. WFO 2018

***Dianthustaniacensis* Gueldenst. ex Ledeb.﻿, Fl. Ross. 1: 287. 1842.** Sec. WFO 2018

***Dianthustatrae* Borbás﻿ in Term. Füz. 12: 221. 1889.** Sec. WFO 2018

***Dianthustenuidens* Jord.﻿, Icon. Fl. Eur. 3: 28. 1903.** Sec. WFO 2018

***Dianthustenuis* F.N.Williams﻿ in Bull. Misc. Inform. Kew 1914: 205. 1914.** Sec. WFO 2018

= *Dianthustenorei* F.N.Williams﻿ in Bull. Misc. Inform. Kew 1914: 206. 1914 syn. sec. WFO 2018

***Dianthustergestinus* Simonk.﻿, Magyar Növényt. Lapok 12: 21. 1888.** Sec. WFO 2018

***Dianthusthessalus* Orph.﻿ in Bull. Congr. Bot. Petersb. 1969: 123. 1870.** Sec. WFO 2018

***Dianthustzopae* Prodán﻿, Fl. Reipubl. Popul. Roman. 2: 671. 1953.** Sec. WFO 2018

***Dianthusvarciorovensis* Prodán﻿, Fl. Reipubl. Popul. Roman. 2: 671. 1953.** Sec. WFO 2018

***Dianthusvariegatus* Jord.﻿, Icon. Fl. Eur. 3: 27. 1903.** Sec. WFO 2018

***Dianthusvelenovskyi* Borbás﻿ in Term. Füz. 14: 46. 1893.** Sec. WFO 2018

***Dianthusverschaffeltii* Lem.﻿ in Ill. Hort. 6: t. 220. 1859.** Sec. WFO 2018

***Dianthusvesulanus* Jord.﻿, Icon. Fl. Eur. 3: 27. 1903.** Sec. WFO 2018

***Dianthusvodnensis* Micevski﻿ in Prilozi Oddel. Biol. Med. Nauki Makedonska Akad. Nauk. Umet. 8: 38. 1987 [“1990”].** Sec. WFO 2018

***Dianthusvukotinovicii* Borbás﻿ in Rad Jugoslav. Akad. Znan. 36: 172. 1876.** Sec. WFO 2018

***Dianthuswettsteinii* Bornm.﻿ in Bull. Herb. Boissier, sér. 2, 5: 60. 1905.** Sec. WFO 2018

***Dianthuswinterlianus* Priszter﻿ in Bot. Közlem. 54: 153. 1967.** Sec. WFO 2018

***Dianthuswolfii* Vetter﻿ in Bull. Murith. Soc. Valais. Sci. Nat. 11: 132. 1883.** Sec. WFO 2018

***Kohlrauschiasibthorpii* Kunth﻿, Fl. Berol., ed. 2, 1: 109. 1838**

= *Kohlrauschiasibthorpii* Kunth﻿, Fl. Berol., ed. 2, 1: 109. 1838 syn. sec. Kew WCVP (2019);

***Tunicaargentii* Meikle﻿ in Kew Bull. 9: 106. 1954**

***Veleziaarabica* Hochst. & Steud. ex Steud.﻿, Nomencl. Bot., ed. 2, 2(2–5): 746. 1841**

### ﻿Excluded names

**Dianthusalpinusvar.glacialis Trautv.﻿ in Trudy Imp. S.-Peterburgsk. Bot. Sada 2: 505. 1873.** Sec. Kuzmina & Nersesyan (2012)

Wrongly attributed to Trautvetter l.c., who had cited Dianthusalpinusvar.glacialis (Haenke) Regel﻿ in Bull. Soc. Imp. Naturalistes Moscou 34(II): 533. 1862

***Dianthusargentii* (Meikle) Govaerts﻿.** Sec. POWO. Plants of the World Online. Facilitated by the Royal Botanic Gardens, Kew.

Listed as ined. in Plants of the World Online, with a remark that the name has been published in Skvortsovia, but no such name was found in this journal.

**Dianthushypoleucusvar.karami Boiss.﻿, Fl. Orient., Suppl.: 80. 1888.** Sec. TPL (2013)

Erroneous TPL record for Dianthushypochlorusvar.karami Boiss.

## Supplementary Material

XML Treatment for Dianthus antalyensis

XML Treatment for Dianthus hispidus

XML Treatment for Dianthus illyricus

XML Treatment for Dianthus illyricus subsp. angustifolius

XML Treatment for Dianthus illyricus subsp. haynaldianus

XML Treatment for Dianthus illyricus subsp. taygeteus

XML Treatment for Dianthus fasciculatus

XML Treatment for Dianthus pachygonus

XML Treatment for Dianthus pseudorigidus

XML Treatment for Dianthus quadridentatus

## Supplementary materials

Supplementary material 1 Appendix 1 Data type Xlsx file. Explanation note Voucher information and ENA accession numbers. https://binary.pensoft.net/file/683690This dataset is made available under the Open Database License (http://opendatacommons.org/licenses/odbl/1.0/). The Open Database License (ODbL) is a license agreement intended to allow users to freely share, modify, and use this Dataset while maintaining this same freedom for others, provided that the original source and author(s) are credited.Georgia Fassou, Nadja Korotkova, Anush Nersesyan, Marcus A. Koch, Panayotis Dimopoulos, Thomas Borsch
